# Peptide Arginases from Cryptic Pathways Install Ornithine Residues in Uncharacterized Members of Orphan RiPP Families

**DOI:** 10.1002/cbic.202500658

**Published:** 2025-11-07

**Authors:** Isabel P.‐M. Pfeiffer, Maria‐Paula Schröder, Panagiota‐Hanna Koutsandrea, Giovanni A. Vitale, Daniela Herrera‐Rosero, Christian Geibel, Daniel Petras, Jörn Piel, Anna L. Vagstad, Silja Mordhorst

**Affiliations:** ^1^ Department of Pharmaceutical Biology University of Tübingen Auf der Morgenstelle 8 72076 Tübingen Germany; ^2^ Functional Metabolomics Lab University of Tübingen Auf der Morgenstelle 24 72076 Tübingen Germany; ^3^ Department of Biology ETH Zürich Institute of Microbiology Vladimir‐Prelog‐Weg 1‐5/10 8093 Zürich Switzerland; ^4^ Present address: University of California Riverside 169 Aberdeen Drive Riverside 92507 CA USA

**Keywords:** enzyme catalysis, new precursor family, ornithine formation, peptide arginases, RiPP biosynthesis

## Abstract

Ribosomally synthesized and post‐translationally modified peptides (RiPPs) are remarkable natural products with interesting chemical structures and potent bioactivities. RiPP pathways are abundant in all domains of life and harbor a large biosynthetic potential in the form of post‐translationally acting enzymes. A relatively small number of RiPP biosynthetic gene clusters encode peptide arginases, a recently discovered maturase family capable of hydrolyzing arginine residues of RiPP core peptides to ornithines. In this study, members of the peptide arginase family (FlmR and OhkR), which are associated with uncharacterized precursors from orphan RiPP families, are identified. In vivo and in vitro activity of FlmR and OhkR with the five associated precursor peptides (FlmA1‐3 and OhkA1‐2) is demonstrated and kinetic studies to biochemically characterize the enzymes are performed. Furthermore, in silico structural analysis with AlphaFold 3 is used to predict precursor–arginase complexes, providing insights into how peptide arginases could bind their precursor substrates. In the case of OhkA–OhkR complexes, this analysis also allows a hypothesis as to which of the arginine residues of the core peptide is modified first, which is confirmed experimentally. This detailed biochemical and structural enzyme characterization is a prerequisite for the application of peptide arginases in peptide‐based drug discovery platforms.

## Graphical abstract

The incorporation of the non‐proteinogenic amino acid ornithine into antibiotic peptides has shown potential in influencing antibacterial efficacy. Novel peptide arginases from cryptic ribosomally synthesized and post‐translationally modified peptide (RiPP) pathways are identified and found to  incorporate ornithine residues into various peptide sequences, complementing the existing peptide arginases and offering new opportunities for drug discovery.

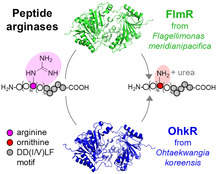

## 1 Introduction

Ribosomally synthesized and post‐translationally modified peptides (RiPPs) are a diverse class of natural products united by their biosynthetic logic. In RiPP biosynthesis, a precursor peptide is synthesized by the ribosome with proteinogenic amino acids as building blocks. Usually, the precursor is comprised of an N‐terminal leader region and a C‐terminal core region. In some cases, a follower region is located at the C‐terminus. Any modifications, such as the formation of non‐canonical amino acids, cyclization, dehydration, and others, are introduced in the core region by various enzymes after translation.^[^ ^1^ ^,^ ^2^ ^]^ Ultimately, the core is released by protease(s), resulting in the formation of the mature RiPP. The post‐translationally acting enzymes are termed maturases, and their function is mostly modulated by the leader region of the precursor peptide. RiPP maturases tend to be very permissive with respect to modifications within the core region,^[^ ^3^, ^4^ ^–^ ^5^ ^]^ as exemplified by the thioether‐forming radical SAM enzyme PapB,^[^ ^6^ ^]^ the prenyltransferase LimF,^[^ ^7^ ^]^ and the class II lanthionine synthetases ProcM^[^ ^8^ ^]^ and SyncM.^[^ ^9^ ^,^ ^10^ ^]^

**Figure 1:**
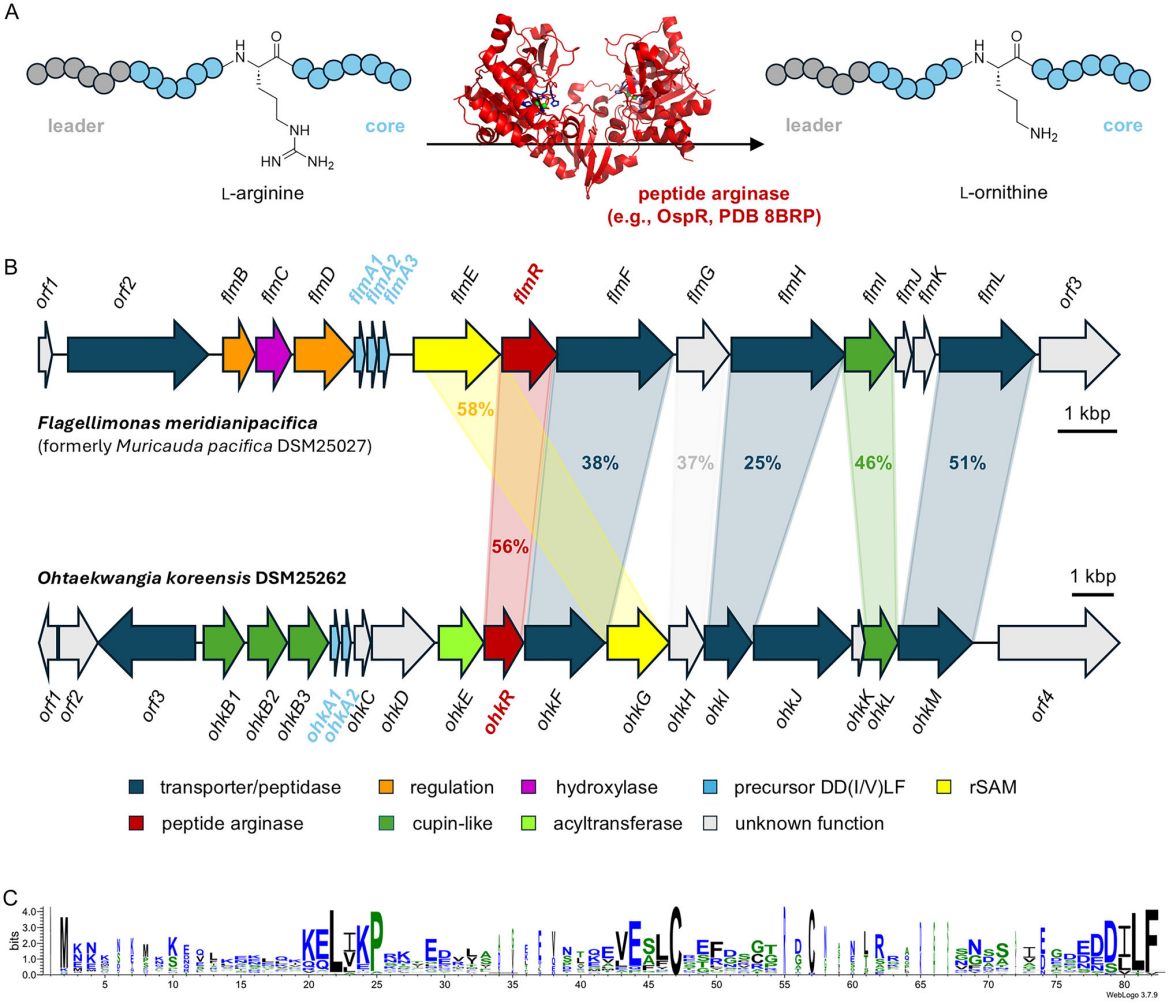
The RiPP family of peptide arginases. A) Post‐translational reaction catalyzed by peptide arginases (e.g., OspR, PDB ID 8BRP). B) RiPP pathways encoding peptide arginases associated with the uncharacterized precursor type "DD(I/V)LF". For detailed gene predictions, see Table S2–S4, Supporting Information. Homologies are expressed as % protein sequence similarity. C) Sequence logo of "DD(I/V)LF"‐type peptide arginase substrates (accession numbers of sequences used for the sequence logo are listed in Table S5, Supporting Information). The sequence logo was built with WebLogo.^[^ ^37^ ^,^ ^38^ ^]^

Peptide arginases (pfam12640) are a novel maturase family; the first representative (OspR, OSCI_RS22075) was identified in 2020.^[^ ^11^ ^]^ OspR is involved in the biosynthesis of landornamide A in the cyanobacterium *Kamptonema* sp. PCC 6506 (formerly *Oscillatoria*). Peptide arginases catalyze the unusual post‐translational transformation of arginine into the non‐canonical amino acid ornithine (**Figure** **1**A), releasing urea as a byproduct.^[^ ^12^ ^]^ Bioinformatic analyses revealed that they are associated with different types of precursor peptides, including the well‐known NHLP (nitrile hydratase leader peptide, which is characteristic of the RiPP family of proteusins^[^ ^13^ ^]^) and N11P (Nif11 nitrogen‐fixing protein^[^ ^13^ ^]^), but also with less common ones, such as SCIFF (Six Cysteines in Forty‐Five residues),^[^ ^14^ ^]^ and completely uncharacterized precursor types, which are provisionally named based on conserved sequence motifs they contain: “SCCRRSCN”, “DD(I/V)LF”, and “MGXRRSSE”.^[^ ^15^ ^]^ So far, seven peptide arginases acting on four different precursor types have been experimentally shown to be active. The most promiscuous of these, OspR, has been reported to be highly tolerant of different positions and numbers of arginine residues within the precursor substrates, and to even accept other precursor types altogether.^[^ ^15^ ^]^ Furthermore, OspR has been utilized in peptide engineering to generate lacticin 481 analogs with antimicrobial activity against *Bacillus subtilis* ^[^ ^16^ ^]^ and to produce optimized semaglutide variants as GLP‐1 receptor agonists for the treatment of Type 2 diabetes and obesity.^[^ ^17^ ^]^ This shows that peptide arginases are promising synthetic tools that can provide a variety of ornithine‐containing peptides as hit compounds for peptide‐based drug discovery.

Ornithine‐containing peptides are interesting drug candidates because it is hypothesized that the incorporation of ornithine has the potential to modulate antibacterial efficacy by affecting interactions with bacterial cell membranes.^[^ ^16^ ^]^ Ornithine moieties are common in products of nonribosomal peptide synthetases (NRPSs), including siderophores (e.g., pyoverdine, erythrochelin) and antibiotics (e.g., bacitracin A, gramicidin S).^[^ ^18^ ^]^ They have also been identified in several RiPP natural products; one example is the antiviral compound landornamide A, which contains two unmodified ornithine residues.^[^ ^11^ ^]^ Such ornithine residues have been shown to serve as building blocks for further modifications, including acetylation, acylation, methylation, and/or hydroxylation. The RiPP family of selidamides^[^ ^19^ ^]^ contains ornithine residues with different fatty acyl moieties attached to the side‐chain *δ*‐nitrogen: phaeornamide A contains a 3‐hydroxy‐decanoic acid on one ornithine side chain, whereas kamptornamide A has three unmodified ornithine residues and one ornithine residue carrying a dodecanoic acid.^[^ ^19^ ^]^ An example of a methylated ornithine residue is found in enteropeptins.^[^ ^20^ ^]^ Enteropeptin A–C are RiPP natural products from the gut microbe *Enterococcus cecorum* with narrow‐spectrum bacteriostatic activity against the producer strain.^[^ ^20^ ^]^ In enteropeptins, ornithine is introduced by KgrD, a Mn^2+^‐dependent arginase belonging to a different protein family (pfam00491) than the peptide arginases described above. The biosynthesis of enteropeptins is initiated by the formation of a thiomorpholine structure from a cysteine residue, involving the C_*α*_ of the adjacent arginine residue. Subsequently, KgrD hydrolyzes the modified arginine to ornithine. Finally, the ornithine is *N*‐methylated by an Fe‐S‐dependent methyltransferase. KgrD shows little sequence similarity to pfam12640 members (15.8% identity with OspR), and its substrate scope seems to be more restricted, since it requires a thiomorpholine to be present in the precursor substrate for activity.^[^ ^20^ ^]^ Another example of a specific peptide arginase is the recently described enzyme PbsE (pfam12640) from *Peribacillus simplex* BE23; this arginase selectively acts on a bis‐hydroxylated aromatic substrate during the biosynthesis of biphenomycins‐like natural products.^[^ ^21^ ^]^

Peptide arginases are metalloenzymes belonging to the ureohydrolase superfamily. Pfam12640 members contain three conserved sequence motifs: DXHXD, EEH(N/H)EAF, and LDIDLDYFSC. The six underlined histidine and aspartate residues coordinate the two manganese metal ions at the active site. In 2023, the first crystal structure of such a peptide arginase was solved (PDB ID 8BRP^[^ ^22^ ^]^), revealing the quaternary structure and active site details.^[^ ^12^ ^]^ The structure of OspR is a three‐layered *α*–*β* *–α* sandwich fold common to all arginases. Interestingly, peptide arginases form homodimers, whereas conventional arginases acting on l‐arginine monomers (EC 3.5.3.1) form trimers (arginases of eukaryotic origin)^[^ ^23^ ^,^ ^24^ ^]^ or hexamers (arginases of bacterial origin).^[^ ^25^ ^]^ This unusual oligomerisation may allow binding of the large peptidyl substrate. It has been hypothesized that the narrow cleft at the center of the homodimer may act as a binding site for the leader part of the precursor peptide. This architecture allows direct access to both active sites (of monomer A and B, Figure 1A). However, the molecular basis of the protein–protein interactions required for substrate recognition is still unclear.

In this study, we sought to expand the scope of peptide arginases. So far, only peptide arginases acting on the precursor types NHLP, N11P, SCIFF, and “SCCRRSCN” have been described.^[^ ^15^ ^]^ Here, we report two novel representatives of this maturase family, FlmR and OhkR, both of which are associated with the previously uncharacterized precursor‐type “DD(I/V)LF” (Figure 1B and C), comprising a putative new RiPP family. We focused on the characterization of the two enzymes by establishing in vivo and in vitro activity for FlmR and OhkR, as well as investigations regarding substrate scope, kinetics, and substrate binding.

## 2 Results and Discussion

### 2.1 Sequence Analysis and Cloning of Peptide Arginases and Precursor Peptides from *flm* and *ohk* Gene Clusters

In a previous bioinformatic analysis of RiPP biosynthetic gene clusters, nine peptide arginase homologs associated with 17 “DD(I/V)LF”‐type precursors were identified.^[^ ^15^ ^]^ These precursors contain a highly conserved sequence motif (“DD(I/V)LF”) at their C‐termini and several arginine residues. The final natural products from these peptide arginase‐containing pathways are still unknown. A further bioinformatic search for “DD(I/V)LF”‐type precursors found in close proximity to peptide arginases was conducted with CluSeek,^[^ ^26^ ^]^ and another 21 biosynthetic gene clusters harboring a total of 40 “DD(I/V)LF”‐type precursor peptides were discovered (Table S5, Supporting Information). The considerable quantity of identified “DD(I/V)LF”‐type proteins emphasizes that these constitute a novel family of orphan RiPPs. Out of the 30 homologous arginases associated with the 57 “DD(I/V)LF”‐type precursors, we chose FlmR (WP_106143370) from *Flagellimonas meridianipacifica* (formerly *Muricauda pacifica* DSM 25027; Tax ID 1080225^[^ ^27^ ^]^) and OhkR (WP_159453773) from *Ohtaekwangia koreensis* DSM 25262 for coexpression experiments in *Escherichia coli*. Both enzymes contain the three conserved sequence motifs DXHXD, EEH(N/H)EAF, and LDIDLDYFSC, which are characteristic of peptide arginases (Figure S1, Supporting Information). The peptide arginase FlmR is associated with three highly similar precursor peptides (FlmA1, FlmA2, and FlmA3), and the *ohk* gene cluster encodes two conserved precursor sequences (OhkA1 and OhkA2, Figure 1B and S2, Supporting Information). Since the mature RiPPs from “DD(I/V)LF”‐type precursors have not yet been characterized, the core peptide sequence and protease cleavage sites remain unknown. Common cleavage site prediction tools such as NLPPrecursor (DeepRiPP)^[^ ^28^ ^]^ and RiPPMiner^[^ ^29^ ^]^ were unable to predict the core peptide sequence. All five precursor sequences contain two arginine residues as potential substrates. The precursor sequences of FlmA1, FlmA3, and OhkA2 contain a third arginine residue, which is hypothesized to be part of the leader peptide and not modified since it is located in the N‐terminal part.

The two strains, *F. meridianipacifica* DSM 25027 and *O. koreensis* DSM 25262, were procured from the German Collection of Microorganisms and Cell Cultures GmbH (DSMZ) and cultivated. Genomic DNA was isolated as a template for PCR reactions. Precursor peptides were cloned as N‐terminal His_6_‐fusion proteins (for details, see Supporting Information). Peptide arginases were cloned with and without His_6_‐tags for in vitro and in vivo experiments, respectively. Low protein yields and instability issues of the first characterized peptide arginase OspR had been resolved by expressing a solubility‐tag fusion with maltose‐binding protein (MBP).^[^ ^12^ ^]^ Therefore, N‐terminal His_6_‐tagged MBP‐fusions of FlmR and OhkR with a tobacco etch virus protease (TEVp) cleavage site between the MBP‐tag and the peptide arginase were also constructed.

### 2.2 In Vivo Coexpression Experiments in *E. coli*

**Figure 2:**
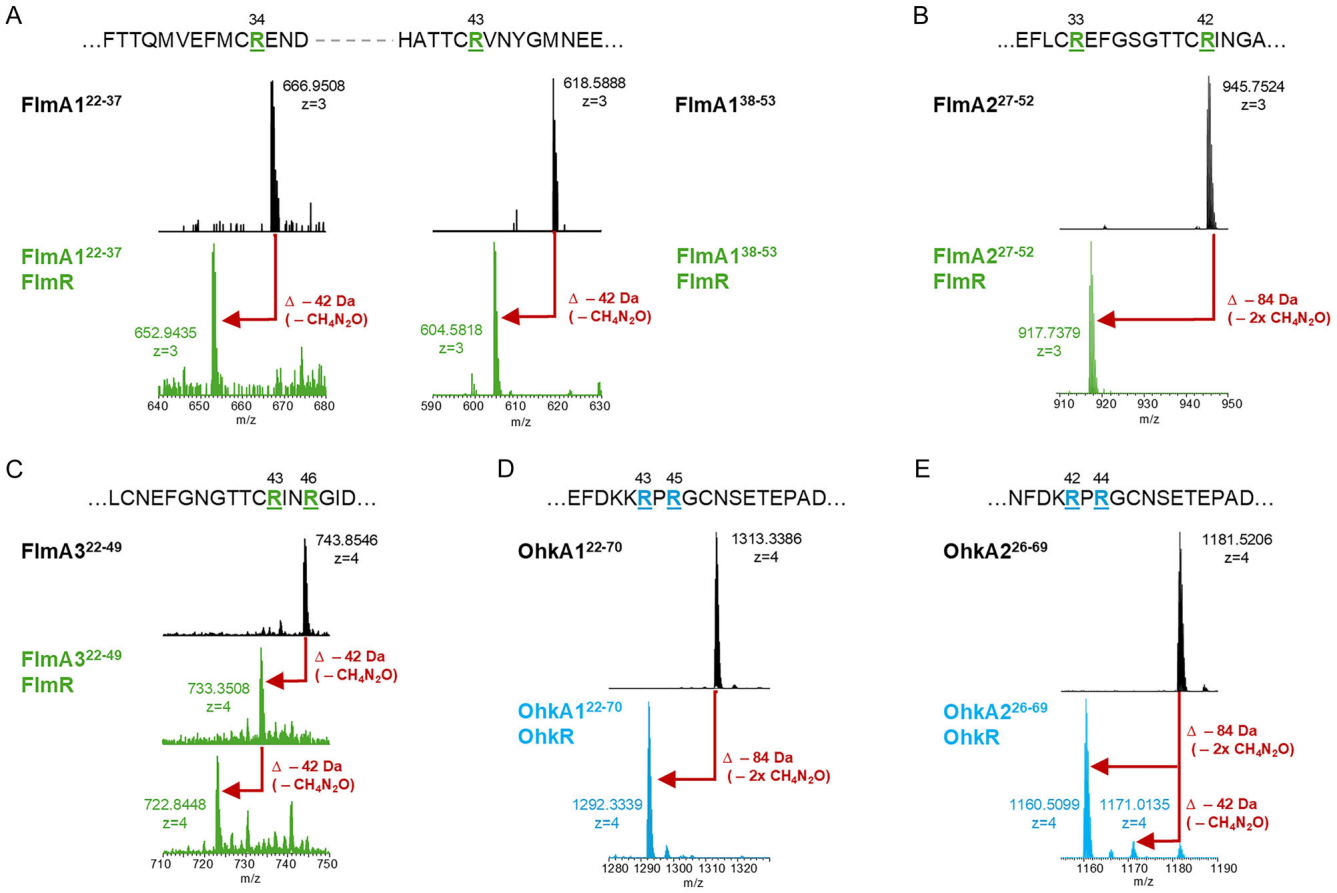
In vivo coexpression experiments with FlmR or OhkR. HPLC‐MS analysis of modified precursor peptides compared to the control (black traces) of A) FlmA1, B) FlmA2, or C) FlmA3 coexpressed with FlmR (green traces) and D) OhkA1 or E) OhkA2 coexpressed with OhkR (blue traces). Full scan spectra of MS1 data are depicted. A mass loss of 42.02 Da per arginine hydrolyzed to ornithine and urea is observed.

Peptide arginases and N‐terminally His_6_‐tagged precursor peptides were heterologously coexpressed in *E. coli* BL21(DE3). Expressions of the precursors alone served as negative controls. Modified and unmodified precursors were purified by Ni^2+^‐affinity chromatography, and precursor production and purity were analyzed by sodium dodecyl sulfate polyacrylamide gel electrophoresis (SDS‐PAGE, Figure S3, Supporting Information). Purified precursor peptides were digested to obtain shorter peptide fragments and, subsequently, the peptide fragments were submitted to reversed‐phase high‐performance liquid chromatography coupled to mass spectrometry (HPLC‐MS/MS). Coexpression of FlmR with FlmA1 produced a fully modified peptide (FlmA1‐R34O‐R43O, O = ornithine) showing a mass loss of 84 Da compared to the unmodified precursor peptide, corresponding to the hydrolysis of two arginine residues (**Figure** **2**A, S4 and S5, Supporting Information). Since the cleavage site between the leader and the core peptide remains unidentified, the residue numbering starts at the beginning of the precursor peptides, rather than at the core peptides, which would be the conventional approach for RiPPs.^[^ ^2^ ^]^ As with FlmA1, coexpression of FlmR and FlmA2 resulted in the complete modification of FlmA2, yielding FlmA2‐R33O‐R42O (Figure 2B and S6, Supporting Information). Coexpression of FlmR and FlmA3 resulted in almost complete conversion to FlmA3‐R43O‐R46O (Figure 2C and S7, Supporting Information). MS/MS analysis confirmed that the mass loss occurred at the arginine residues (Figure S4‐S7, Supporting Information). As anticipated, Arg4^FlmA1^ and Arg5^FlmA3^ were not hydrolyzed to ornithine, thus validating the hypothesis that these residues are part of the leader peptide (data not shown). For FlmA1, the directionality of arginine to ornithine conversions could not be resolved, as unmodified and modified species are observed for both peptide fragments FlmA1^22−37^ and FlmA1^38−53^ (Figure S8, Supporting Information). For FlmA2 and FlmA3, minor singly modified intermediates containing one arginine, and one ornithine residue could be detected. The presence of FlmA2‐R33O and the absence of FlmA2‐R42O indicate that FlmR acts on the substrate FlmA2 from the N‐terminus toward the C‐terminus (Figure S9, Supporting Information), as has been demonstrated for the peptide arginases OspR, BlhR, and PacR.^[^ ^15^ ^]^ In contrast, the substrate FlmA3 appears to be processed in the reverse direction (from the C‐terminus to the N‐terminus), since the intermediate FlmA3‐R46O is detected, while the potential intermediate FlmA3‐R43O is absent (Figure S10, Supporting Information). This C‐to‐N directionality has not been previously observed in the context of peptide arginases.

For the second peptide arginase analyzed here, OhkR, full conversion of the precursor peptide OhkA1 was observed (Figure 2D and S11, Supporting Information), while a small amount of unmodified and intermediate (containing one arginine and one ornithine residue) was observed in the coexpression of OhkR and OhkA2 (Figure 2E and S12,Supporting Information). As expected, Arg17^OhkA2^ was not converted to ornithine, again supporting the hypothesis that this residue belongs to the leader peptide (data not shown). In coexpressions with the MBP‐tagged OhkR, a relatively large amount of singly modified precursors allowed us to determine the directionality of the arginine‐to‐ornithine conversions (Figure S13 and S14, Supporting Information). In OhkA1, Arg45 is modified before Arg43, since only the intermediate OhkA1‐R45O is present (Figure S15, Supporting Information). Similarly, only the intermediate OhkA2‐R44O was observed, indicating that Arg44 is modified before Arg42 in OhkA2 (Figure S16, Supporting Information). Consequently, OhkR seems to proceed from the C‐terminus to the N‐terminus as FlmR does when converting FlmA3.

We next tested the cross‐reactivity between the two arginases and coexpressed OhkR with FlmA1−3 and FlmR with OhkA1−2. OhkR also accepts FlmA1−3 as substrates and converts the core peptide arginine residues to ornithine residues (Figure S17−19, Supporting Information). The conversion is not complete, and unmodified and singly modified peptide species are observed. Similarly, FlmR exhibited partial conversion of OhkA1 and OhkA2; minor amounts of singly modified OhkA1 were detected (Figure S20, Supporting Information), and a small amount of 1‐ornithine‐containing OhkA2, along with trace amounts of fully modified OhkA2, could be observed in the coexpression of FlmR and OhkA2 (Figure S21, Supporting Information).

In order to investigate the role of the conserved “DD(I/V)LF” motif, two precursor variants were constructed. In the A1 precursors, the conserved motif was replaced with alanine residues to create the peptide variants FlmA1‐D53A‐I54A‐L55A‐F56A and OhkA1‐D66A‐D67A‐V68A‐L69A‐F70A. These constructs were then coexpressed with their native maturases and analyzed by HPLC‐MS*.* Replacing the conserved motif with alanine residues had no effect on FlmR catalysis, with full conversion of the FlmA1‐D53A‐I54A‐L55A‐F56A precursor observed (Figure S22, Supporting Information). In the case of OhkR, however, replacing the motif with alanine residues yielded a mixture of fully modified, singly modified, and unmodified peptide species (Figure S22, Supporting Information). Consequently, the precursor variant is still accepted as a substrate, but conversion is less efficient.

### 2.3 Kinetic Characterization of FlmR and OhkR In Vitro

His_6_‐FlmR was heterologously produced in *E. coli* in sufficient amounts for in vitro assays (Figure S3, Supporting Information). In contrast, His_6_‐OhkR could not be produced and purified and was produced as the His_6_‐MBP‐OhkR solubility tag fusion. Attempts to cleave the MBP‐tag with TEVp after protein purification by Ni^2+^‐affinity column chromatography were not successful despite using an optimized TEVp cleavage site (for details, see Supporting Information). Due to the high sequence similarity exhibited by the FlmA and OhkA precursors (Figure S2, Table S6, Supporting Information), a single precursor (A1) was chosen for the reaction with each peptide arginase with the aim of determining Michaelis–Menten kinetic rate constants. The remaining precursors were then subjected to a comparative analysis in a relative rate experiment. The His_6_‐tagged precursor peptides FlmA1, FlmA2, FlmA3, OhkA1, and OhkA2 were heterologously produced in *E. coli* and purified by Ni^2+^‐affinity chromatography (Figure S3, Supporting Information).

**Figure 3:**
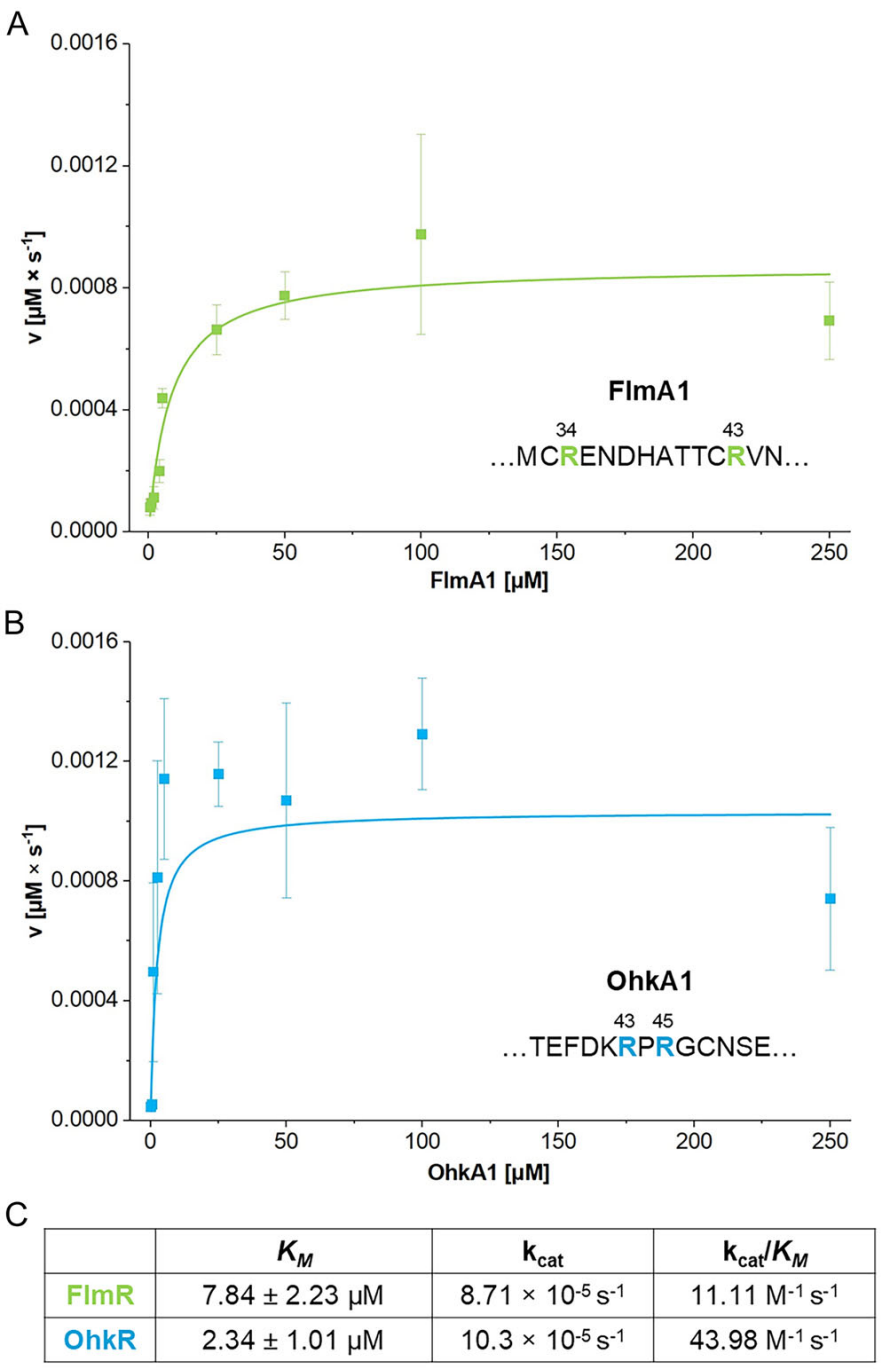
In vitro catalysis of FlmR and OhkR. A) Michaelis–Menten kinetics of FlmR with FlmA1. The substrate arginine residues are highlighted in green. B) Michaelis–Menten kinetics of OhkR with OhkA1. The substrate arginine residues are highlighted in blue. C) The table shows an overview of the Michaelis–Menten constant (*K*_*M*_), the catalytic constant/turnover number (k_cat_), and the catalytic efficiency (k_cat_/*K*_*M*_) for both peptide arginases. Mean values ± standard deviation are depicted.

For Michaelis–Menten kinetic analyses, the same (optimal) conditions that had previously been identified for OspR^[^ ^12^ ^]^ were applied (50 mM TRIS‐HCl pH 8.5, 1 mM MnCl_2_, 25 °C) to allow comparison of the kinetic data. The urea coproduct of arginine hydrolysis was detected by a commercially available end‐point spectrophotometric assay at a wavelength of 430 nm.^[^ ^30^ ^]^ Multiple substrate concentrations ranging from 0.5 µM to 250 µM (FlmA1) or from 0.1 µM to 250 µM (OhkA1) were tested with their cognate arginase and the reactions were stopped by addition of the ethylenediaminetetraacetic acid (EDTA) at selected time points. All reactions were performed in triplicate. The *K*_M_ value for FlmR toward its substrate FlmA1 was calculated to be 7.8 µM, and the k_cat_ was found to be 8.7 × 10^−5^ s^−1^ (**Figure** **3**A, Table S12, Supporting Information). For OhkR, the *K*_*M*_ value is 2.3 µM (using OhkA1 as a substrate), and the k_cat_ 10.3 × 10^−5^ s^−1^ (Figure 3B, Table S12, Supporting Information). The catalytic constants (k_cat_ values) are quite low; this slow conversion is typical for enzymes involved in specialized metabolism compared to enzymes from primary metabolism.^[^ ^31^ ^]^ The peptide arginase OspR showed similar results (k_cat_ 5 × 10^–3^ s^–1^, *K*_M_ 9 µM for unmodified OspA)^[^ ^12^ ^]^; while conventional arginases, such as human arginase‐1, have a significantly higher turnover (k_cat_ 300 s^–1^) but a lower substrate affinity (*K*_M_ 2.3 mM for l‐arginine).^[^ ^32^ ^]^

Relative rates of FlmR and OhkR toward the five different precursor peptides were determined to compare their substrate conversions. The reactions were performed in triplicate and stopped at six selected time points. The relative rates were calculated for the initial time points in the linear range of ornithine and urea formation (Figure S23 and S24, Table S13, Supporting Information). Interestingly, FlmR prefers FlmA2 and FlmA3 (100% and 97%, respectively) over FlmA1 (41%), while OhkR shows rather similar conversion rates for OhkA1 and OhkA2 (82% and 91%, respectively). In the precursor swap experiments, FlmR shows conversion rates of 17% and 26% for OhkA1 and OhkA2, respectively. Surprisingly, OhkR showed the fastest conversion toward the non‐native FlmA1 precursor, while FlmA3 conversion was similar to the native precursors and FlmA2 turnover was significantly lower (47%). In addition, we tested whether FlmR and OhkR could accept free l‐arginine as a substrate. For the first representative of peptide arginases, OspR, it has been shown that neither l‐arginine nor the peptidyl‐arginine mimetic *N‐*acetylarginamide was converted at a substrate concentration of 250 µM.^[^ ^12^ ^]^ Since the *K*_*M*_ of human arginase‐1 toward its native substrate l‐arginine is 2.3 mM, it was decided to test a higher substrate concentration. Indeed, a small amount of urea formation was detected in FlmR and OhkR assays at a concentration of 1 mM l‐arginine (Figure S25, Supporting Information). This indicates that peptide arginases prefer peptide chains for substrate recognition but are also able to convert free arginine in small amounts.

### 2.4 Structural Models of FlmR and OhkR

AlphaFold 3 (AF3)^[^ ^33^ ^]^ was employed for structure prediction of the two novel peptide arginases. To evaluate the accuracy of AF3 predictions, the structure of OspR was modeled and compared to the reported crystal structure of OspR (PDB 8BRP). The structural alignment of the two OspR proteins (model vs. X‐ray structure) revealed a root mean square deviation (RMSD) of 0.510 (240 to 240 C_*α*_ atoms) for the overall protein and a RMSD of 0.161 (6 to 6 C_*α*_ atoms) for the six metal‐coordinating residues of the active site. This demonstrates that the AF3 model is highly similar to the experimentally determined crystal structure of OspR.

**Figure 4:**
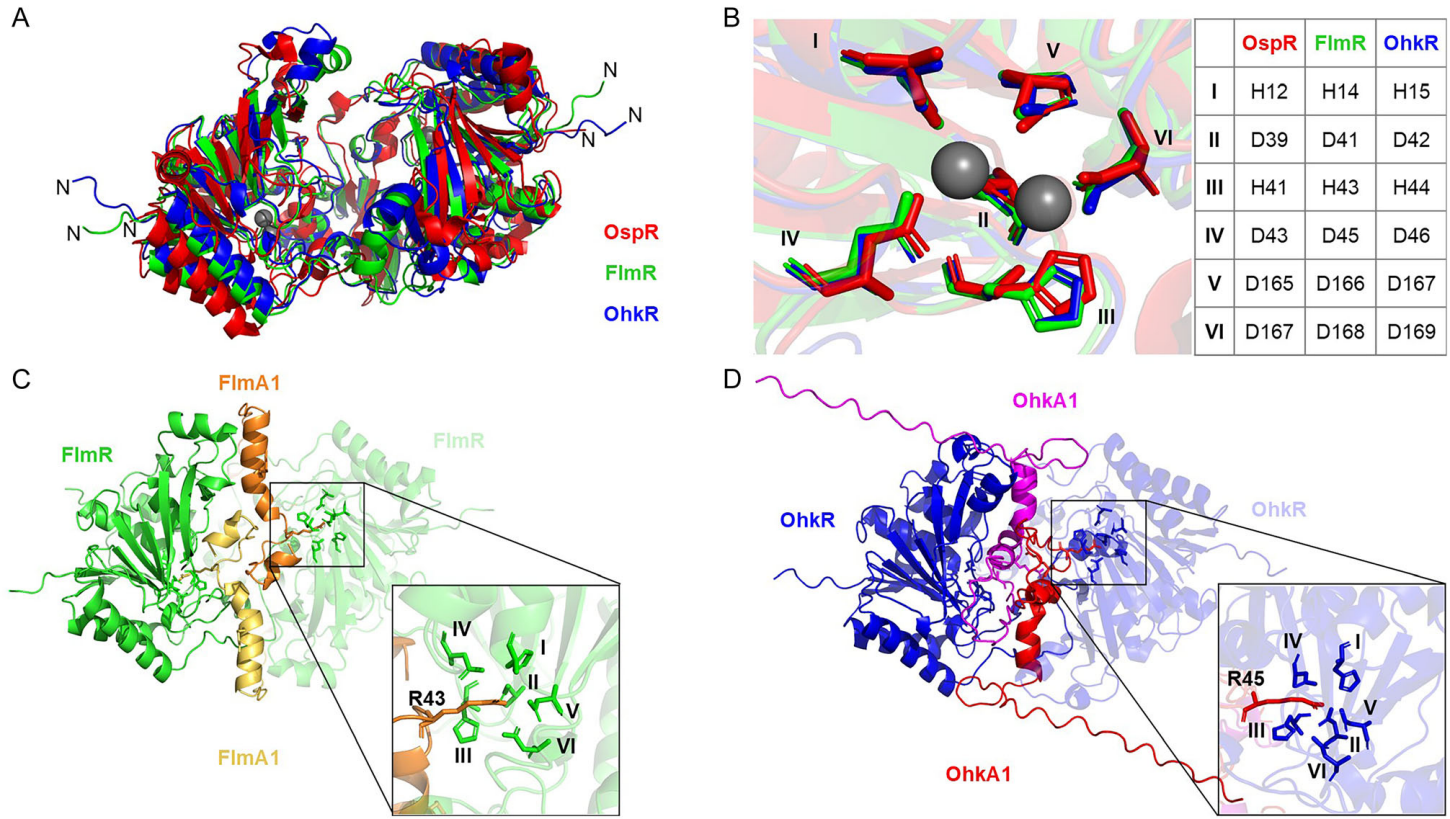
Structural analysis of OhkR and FlmR with AlphaFold 3 (AF3). A) Structural alignment of the OspR (red) crystal structure with the AF3 models of FlmR (green) and OhkR (blue). B) Structural alignment of the active site residues and coordinated Mn^2+^ ions (gray spheres) of OspR, FlmR, and OhkR. C) AF3 model of the FlmR dimer with two FlmA1 monomers (yellow and orange). R43^FlmA1^ is coordinated by the active site residues (zoomed‐in box). D) AF3 model of the OhkR dimer with two OhkA1 monomers (red and magenta). R45^OhkA1^ is coordinated by the active site residues (zoomed‐in box).

Since peptide arginases have been described as homodimers,^[^ ^12^ ^]^ two entities of the protein sequence were utilized for the prediction (Table S14, Supporting Information). The two structures of FlmR and OhkR were modeled with a high level of confidence (average pLLDT value > 90).^[^ ^34^ ^]^ As anticipated, a structural alignment of FlmR (AF3 model) or OhkR (AF3 model) with OspR (PDB 8BRP) demonstrated a high similarity for the overall protein structure (C_*α*_‐RMSD: 4.385 and 4.881) and a very high similarity for the active site residues (C_*α*_‐RMSD: 0.222 and 0.217). An even higher structural similarity was shown for the two modeled structures of FlmR and OhkR (C_*α*_‐RMSD: 0.723) with an almost identical alignment of the active site residues (C_*α*_‐RMSD: 0.093, **Figure** **4**A,B).

### 2.5 In Silico Substrate Binding Studies

To analyze substrate binding of peptide arginases, AF3 was utilized to predict precursor–arginase complexes. Since peptide arginases form homodimers, two arginase entities and one or two precursor entities were employed (Figure 4C, D, S26 and S27, Supporting Information). Models with only one precursor (and two arginase entities) were not reproducible and yielded inconclusive results. In contrast, the models containing two arginases and two precursors produced highly similar complexes in five independent predictions. This suggested that a catalytic complex is formed by two arginase monomers and two precursor monomers, as was also proposed for the NHLP‐type arginase OspR.

Peptide arginases are metalloenzymes that contain a binuclear manganese cluster; therefore, four Mn^2+^ ions (two ions for each arginase monomer) were included in the comodeling of FlmR and FlmA. However, Mn^2+^ addition led to a different prediction, where the precursor residues are no longer modeled in the active site, which is now occupied by the manganese ions (data not shown). We hypothesize that the maturase–precursor interaction primarily depends on the leader peptide or the residues surrounding the peptidyl arginine substrate. Although Mn^2+^ ions are necessary for coordinating a hydroxide ion and initiating catalytic turnover, they are probably not required for the initial binding of the substrate. Therefore, further comodeling experiments were performed without manganese. In the FlmR–FlmA1 model, R43^FlmA1^ is coordinated by the active site residues H14^FlmR^, D41^FlmR^, D45^FlmR^, D166^FlmR^, and D168^FlmR^. Likewise, R42^FlmA2^ is coordinated by these five residues in the FlmR–FlmA2 model. For FlmR–FlmA3, R46^FlmA3^ contact was predicted to the active site residues H43^FlmR^ and D45^FlmR^. In all three complexes, the arginine residue located closer to the C‐terminus of the precursor peptide is modeled in the active site. For FlmA3, experimental data showed that the C‐terminal arginine residue (R46^FlmA3^) is modified first, but for FlmA2, experimental data support the N‐to‐C‐terminal directionality. For the OhkR–OhkA1 model, R45^OhkA1^ is coordinated by the active site residues H15^OhkR^, D42^OhkR^, H44^OhkR^, D46^OhkR^, D167^OhkR^, and D169^OhkR^. Similarly, for OhkR–OhkA2, R44^OhkA2^ is modeled adjacent to the active site residues of OhkR. AlphaFold predictions for OhkR agree with the in vivo data, where OhkR appears to have a C‐to‐N‐terminal directionality.

In order to simulate the binding of a singly modified precursor, one arginine was substituted with a lysine residue to mimic ornithine. Since the AlphaFold server accepts only the 20 proteinogenic amino acids, lysine was selected as the replacement for ornithine based on their high structural similarity. The two precursor variants FlmA1‐R34K and FlmA1‐R43K were modeled in complex with FlmR. As expected, R43^FlmA1−R34K^ was still predicted in the FlmR active site, since it is the only arginine residue in the core region. Intriguingly, R34^FlmA1−R43K^ was now predicted in the active site in the FlmA1‐R43K model, perfectly mimicking the catalytic activity of FlmR, which first hydrolyzes R43 and subsequently R34. In the FlmA2‐R33K model, again, R42^FlmA2−R33K^ was predicted in the active site; for FlmA2‐R42K, neither R33^FlmA2−R42K^ nor any other residue of FlmA2 was reproducibly predicted in the active site. In the model of FlmA3‐R43K, no precursor residue was located close to the FlmR active site, while R43^FlmA3−R46K^ was predicted in the active site for the FlmA3‐R46K model. Whether there is a defined order of arginine modification that applies to all three FlmA precursors remains unclear based on the AlphaFold models. In singly modified precursor models of OhkR–OhkA1, two precursor variants were modeled: OhkA1‐R43K and OhkA1‐R45K. As expected, R45^OhkA1−R43K^ is modeled in coordination with the active site residues for OhkA1‐R43K. The OhkA1‐R45K model shows R43^OhkA1−R45K^ pointing toward the active site, and these results are in accordance with the in vivo data. For OhkR–OhkA2, the variants OhkA2‐R42K and OhkA2‐R44K were modeled. As expected, the OhkA‐R42K prediction showed R44^OhkA2−R42K^ coordinated by the active site residues. Interestingly, the OhkA2‐R44K model still showed K44^OhkA2−R44K^ in the active site, with R42^OhkA2−R44K^ pointing in the opposite direction. Additionally, OhkA2‐R42K‐R44K was modeled; in this case, K44^OhkA2−R42K−R44K^ was still predicted to be coordinated by the active residues.

All five of the predicted precursors contain both *α*‐helical and unstructured parts. The *α*‐helix extends from T26^FlmA1^ to ≈T40^FlmA1^, S24^FlmA2^ to ≈S37^FlmA2^, T22^FlmA3^ to ≈G39^FlmA3^, L30^OhkA1^ to ≈K42^OhkA1^, and L29^OhkA2^ to ≈K41^OhkA2^. Based on the predictions, it is not possible to draw any conclusions about where the core peptide begins. For FlmA1 and FlmA2, parts of the core peptide are involved in the *α*‐helical fold, since this region encompasses R34^FlmA1^ and R33^FlmA2^. For FlmA3, OhkA1, and OhkA2, the core peptide arginine residues are located in the unstructured regions.

The protein–substrate interfaces were analyzed with the PDBePISA (PISA = Proteins, Interfaces, Structures, and Assemblies) tool (Table S15, Supporting Information).^[^ ^35^ ^]^ The average interface area of homodimer subunits for FlmR and OhkR models is ≈ 2400 Å^2^, which is higher than the average interface area of the OspR crystal structure (1864 Å^2^). The dimerization domain found in OspR is highly similar to the domains identified in FlmR and OhkR, and thus, the dimer contact region is also similar to that of OspR (Ser170–Ser234), spanning from Pro176–Tyr229 in FlmR and from Pro177–Pro233 in OhkR. *Δ* ^i^G *p*‐values below 0.5 imply that the interface area is interaction‐specific. For the FlmR homodimer, *p*‐value is 0.565, so slightly above the threshold, but since it is a structure prediction and not an experimental crystal structure, we still assume the interaction to be specific. For OhkR, the homodimer *p*‐value is 0.112. The average precursor–arginase interface is 1645 Å^2^ for FlmA and 1511 Å^2^ for OhkA, corresponding to 26.2% and 20.3% of precursor total surface area, respectively. For FlmR–FlmA models, *p*‐values between 0.175 and 0.317 were calculated, which indicates that the interaction is specific, while for OhkR–OhkA models the *p‐*values range between 0.532 and 0.542, which is slightly above the threshold and could be due to the AF3 prediction. In FlmR–FlmA models, the majority of the precursor residues interact with the arginase; 40 (of 56 residues in total), 38 (of 55), and 34 (of 56) residues have at least 10% buried surface area in FlmA1, FlmA2, and FlmA3, respectively. These residues are distributed along the entire length of the precursor peptide, indicating that the interaction extends beyond the leader peptide to encompass the core peptide. The conserved "DD(I/V)LF" motif is predicted to interact with FlmR, and in FlmA1 and FlmA3, all residues of this conserved motif show at least 10% buried surface area, while in FlmA2, the first residue, D52^FlmA2^, does not make contact with FlmR, but I53^FlmA2^, L54^FlmA2^, and F55^FlmA2^ do. In the case of the OhkR–OhkA models, ≈50% of the precursor residues show at least 10% buried surface area; it is predicted that 36 of the 70 residues in OhkA1 and 33 of the 69 residues in OhkA2 interact with the arginase. Similar to the FlmR–FlmA models, the "DD(I/V)LF" motif is involved in the interaction between the precursor and the maturase. While all motif residues in OhkA1 have a buried surface area of at least 10%, D65^OhkA2^ has none. However, the rest of the motif (D66^OhkA2^, V67^OhkA2^, L68^OhkA2^, and F69^OhkA2^) makes contact with OhkR. In OhkR–OhkA models, the first 15–17 N‐terminal residues of OhkA1/2 do not interact with OhkR, suggesting that this region may not be necessary for substrate recognition. To test this hypothesis, the N‐terminus of the two precursors was deleted to create the variants OhkA1^16−70^ and OhkA2^18−69^. These precursor variants were then coexpressed with OhkR. HPLC‐MS analysis of the digested precursor peptides confirmed that the N‐terminal region is not required for substrate recognition, since the conversion of the precursor variants OhkA1^16−70^ and OhkA2^18−69^ was similar to that of the native precursors (Figure S28, Supporting Information).

## 3 Conclusion

In this study, we established in vivo and in vitro activity for two new representatives of the RiPP maturase family of peptide arginases. FlmR and OhkR act on an unprecedented RiPP precursor type from an orphan RiPP family, which displays a highly conserved “DD(I/V)LF” sequence motif at the C‐terminus. The present study does not resolve the question of whether this sequence motif is post‐translationally modified by other maturases encoded in the *flm* and *ohk* biosynthetic gene clusters and part of the core peptide, or whether it is a follower region, which is required for substrate recognition and finally cleaved to release the final natural product. These questions will be pursued in a follow‐up project, while we focused on the characterization of two novel peptide arginases here. Besides the kinetic characterization, we studied the precursor–arginase catalytic complex using AlphaFold 3. The AF3 predictions allowed us to postulate the directionality of arginine‐to‐ornithine conversions that could experimentally be confirmed for the FlmA3, OhkA1, and OhkA2 precursor peptides.

The enzymes FlmR and OhkR complement the characterized members of the peptide arginase family, thereby expanding the range of enzymes capable of installing ornithine residues into diverse peptide sequences. This is a fascinating area of research, given the established fact that positively charged amino acids, such as arginine, lysine, and ornithine, have the capacity to influence the antibacterial activity of peptide drugs. For instance, polycationic peptides have been fused to daptomycin to overcome antibiotic resistance.^[^ ^36^ ^]^

## Conflict of Interest

The authors declare no conflict of interest.

## Author Contributions

**Isabel Pia‐Marie Pfeiffer**: data curation (equal); visualization (equal); writing—original draft (supporting). **Maria‐Paula Schröder**: data curation (equal); visualization (equal); (writing)—original draft (supporting). **Panagiota‐Hanna Koutsandrea**: data curation (equal); visualization (equal); writing—original draft (supporting); writing—review & editing (supporting). **Giovanni Andrea Vitale**: data curation (supporting). **Daniela Herrera‐Rosero**: data curation (supporting). **Christian Geibel**: data curation (supporting). **Daniel Petras**: funding acquisition (supporting); supervision (supporting). **Jörn Piel**: funding acquisition (supporting); supervision (supporting). **Anna Lisa Vagstad**: conceptualization (supporting); supervision (supporting); writing—review & editing (supporting). **Silja Mordhorst**: conceptualization (lead); data curation (supporting); funding acquisition (lead); project administration (lead); supervision (lead); visualization (supporting); writing—original draft (lead); writing—review & editing (lead). **Isabel P.‐M.** **Pfeiffer**, **Maria‐Paula** **Schröder**, and **Panagiota‐Hanna Koutsandrea** contributed equally to this work.

## Supporting information

Supplementary Material

## Data Availability

The data that support the findings of this study are available in the supplementary material of this article.
