# Supplementary material for: Peptide Arginases from Cryptic Pathways Install Ornithine Residues in Uncharacterized Members of Orphan RiPP Families
Source: Chembiochem. 2025 Nov 7;26(24):e202500658. doi: 10.1002/cbic.202500658 (PMC12703449; doi:10.1002/cbic.202500658)
Supplement: Supplementary file 1 — Supplementary Material [file CBIC-26-e202500658-s001.pdf]

Supporting Information

Peptide Arginases from Cryptic Pathways Install Ornithine Residues in Uncharacterised Members of Orphan RiPP Families

Isabel P.-M. Pfeiffer, Maria-Paula Schröder, Panagiota-Hanna Koutsandrea, Giovanni A. Vitale, Daniela Herrera-Rosero, Christian Geibel, Daniel Petras, Jörn Piel, Anna L. Vagstad, and Silja Mordhorst*

Abstract: Ribosomally synthesised and post-translationally modified peptides (RiPPs) are remarkable natural products with interesting chemical structures and potent bioactivities. RiPP pathways are abundant in all domains of life and harbour a large biosynthetic potential in the form of post-translationally acting enzymes. A relatively small number of RiPP biosynthetic gene clusters encode peptide arginases – a recently discovered maturase family capable of hydrolysing arginine residues of RiPP core peptides to ornithines. In this study, we identified members of the peptide arginase family (FlmR and OhkR), which are associated with uncharacterised precursors from orphan RiPP families. We demonstrate *in vivo* and *in vitro* activity of FlmR and OhkR with the five associated precursor peptides (FlmA1-3 and OhkA1-2) and performed kinetic studies to biochemically characterise the enzymes. Furthermore, *in silico* structural analysis with AlphaFold 3 was used to predict precursor–arginase complexes providing insights into how peptide arginases could bind their precursor substrates. In case of the OhkA–OhkR complexes, this analysis also allowed a hypothesis as to which of the arginine residues of the core peptide is modified first, which was confirmed experimentally. Our detailed biochemical and structural enzyme characterisation is a prerequisite for the application of peptide arginases in peptide-based drug discovery platforms.

Table of Contents

Table of Contents 2
Experimental Procedures 3
Materials 3
Cloning of Constructs for Heterologous (Co-)Expression 3
DNA Sequences 5
Protein Sequences 6
(Co-)Expression, Precursor and Arginase Purification, and Peptide Digests 8
HPLC-MS/MS Analysis 8
Enzyme Activity Assays/Kinetic Analyses 8
Statistical Analysis 8
Results and Discussion 9
Bioinformatic Analysis of Genetic Region Surrounding the *flm* and *ohk* Peptide Arginases 9
Sequence Analysis of Peptide Arginases 12
Sequence Analysis of ‘DD(I/V)LF’-type Precursors 13
Protein Expression Yield 15
SDS-PAGE Analysis of (Modified) Precursor Peptides and Peptide Arginases 15
Mass Spectrometry Data 16
Activity of FlmR on its Native Substrates FlmA1, FlmA2, and FlmA3 (*in vivo*) 19
Directionality of arginine-to-ornithine conversion for FlmR 23
Activity of OhkR on its Native Substrates OhkA1 and OhkA2 (*in vivo*) 25
Directionality of arginine-to-ornithine conversion for OhkR 29
Precursor Swapping: Activity of OhkR on the FlmR Substrates FlmA1, FlmA2, and FlmA3 (*in vivo*) 30
Precursor Swapping: Activity of FlmR on the OhkR Substrates OhkA1 and OhkA2 (*in vivo*) 32
Alanine replacement of the conserved ‘DD(I/V)LF’ motif of FlmA1 and OhkA1 precursors 33
Michaelis-Menten kinetics for FlmR and OhkR 34
In silico Structural Analysis of Precursors, Arginases, and Precursor-Arginase Binding 35
Analysis of precursor variants: Deletion of OhkA1 and OhkA2 N-termini 39
References 40

Experimental Procedures

Materials

Chemicals and media components were purchased from Carl Roth GmbH & Co. KG, Merck KGaA, and Thermo Fisher Scientific Inc. in the highest purity available. PCR custom oligonucleotides were ordered from Eurofins Genomics GmbH and Microsynth AG (Switzerland). Restriction enzymes, T4 DNA Ligase, Gibson Assembly Master Mix, KLD Mix, the endoproteinases LysC and GluC, as well as GeneRuler 1kb DNA Ladder were from New England Biolabs GmbH. PageRuler^{TM} Plus Prestained protein ladder came from Thermo Fisher Scientific Inc. Kanamycin sulphate was from Carl Roth GmbH & Co. KG and streptomycin sulphate from Merck KGaA. Formic acid for the proteolytic digest was from Carl Roth GmbH & Co. KG. The Urea Assay Kit QuantiChrom^{TM} (DIUR-100) was purchased from BioAssay Systems.

Cloning of Constructs for Heterologous (Co-)Expression

<u>Restriction enzyme-based cloning of peptide arginase and precursor genes from *Flagellimonas meridianipacifica* (formerly *Muricauda pacifica*) and *Ohtaekwangia koreensis*</u>

The *E. coli* DH5α strain (Invitrogen) was employed for the purpose of plasmid amplification, maintenance, and cloning. The genes *flmR*, *flmA1*, *flmA2*, *flmA3*, *ohkR*, *ohkA1*, and *ohkA2* were amplified from genomic DNA by PCR using the primer pairs listed in Table S1. DNA was visualised by gel electrophoresis on 1% (w/v) agarose gels supplemented with ethidium bromide in TAE buffer. The gel-purified gene fragments were subsequently digested. The precursor genes (*flmA1*, *flmA2*, *flmA3*, *ohkA1*, and *ohkA2*) were digested with NdeI and HindIII and ligated with pET28a using T4 DNA ligase yielding the N-terminally His_6-tagged fusion constructs pET28a-*flmA1* (Kan^R), pET28a-*flmA2* (Kan^R), pET28a-*flmA3* (Kan^R), pET28a-*ohkA1* (Kan^R), and pET28a-*ohkA2* (Kan^R). The peptide arginase genes were digested with XhoI and FseI (*flmR*) or NdeI (*ohkR*), and ligated into the MCSII of pCDFDuet-I, yielding the untagged arginase constructs pCDFDuet-I-*flmR* (Sm^R) and pCDFDuet-I-*ohkR* (Sm^R). Subsequently, electrocompetent cells were transformed with the ligation mixtures and plated on LB agar with the appropriate antibiotics. Resulting colonies were then miniprepped and sequenced to verify the insertion of the gene fragment. The plasmids constructed have undergone verification by sequencing (Microsynth AG, Switzerland).

<u>Cloning of tagged peptide arginases</u>

For *in vitro* activity studies, the peptide arginases need to be purified. Since the (prototype) peptide arginase OspR was unstable *in vitro* without a solubility tag,[1] N-terminal maltose-binding protein tags (MBP-tags) were fused to OhkR and FlmR to generate His_6-MBP-arginase constructs. The cloning procedure of the template plasmid pCDF-MBP-*ospR* (Sm^R) was previously reported.[1] A tobacco etch virus protease (TEVp) cleavage site (EGLYFQ) between the MBP-tag and the arginase should theoretically allow the tag to be removed after purification. However, attempts to remove the MBP-tag of OspR by TEVp digestion resulted in only incomplete cleavage products.[1] For more efficient tag removal, the most efficient motif for TEV cleavage, E<u>**N**</u>LYFQ,[2] should be installed. Therefore, the template plasmid pCDF-MBP-*ospR* (Sm^R) was treated with KLD enzyme mix (New England Biolabs) subsequent to PCR amplification with the primer pair P_MBP_Mutag_FW and P_MBP_Mutag_RV (Table S1). Thereby, glycine was exchanged with asparagine within the TEV recognition sequence. Electrocompetent *E. coli* XL1-Blue cells were transformed with the KLD mixture, and the resulting cell suspension was plated on LB agar containing the appropriate antibiotic. Plasmid isolation of the obtained colonies was conducted by alkaline lysis and the resulting plasmid pCDF-MBPopt-*ospR* (Sm^R) was verified by enzymatic digest and sequencing (Eurofins Genomics, Germany).

The above-described plasmid pCDFDuet-I-*ohkR* (Sm^R) and the newly constructed pCDF-MBPopt-*ospR* (Sm^R) were digested with NdeI/XhoI and purified by preparative agarose gel electrophoresis, yielding the insert *ohkR* and the vector backbone pCDF-MBPopt (Sm^R), respectively. The purified DNA fragments were ligated using T4 DNA ligase. Electrocompetent *E. coli* XL1-Blue cells were transformed with the ligation mixture and the constructed plasmid pCDF-MBPopt-*ohkR* (Sm^R) was isolated and verified as described above.

Preparation of the vector pCDF-MBPopt (Sm^R) for cloning of *flmR* was accomplished as described for the cloning of *ohkR*. Contrastingly, the insert was amplified by PCR with the primer pair P_FlmR_FW and P_FlmR_RV (Table S1) utilising pCDFDuet-I-*flmR* (Sm^R) as the template. The purified PCR product was digested with NdeI/XhoI and ligated into pCDF-MBPopt (Sm^R) using T4 DNA ligase. Transformation, plasmid isolation, and verification of pCDF-MBPopt-*flmR* (Sm^R) were conducted as described above.

Even with the optimised TEV cleavage site E<u>**N**</u>LYFQ, the removal of the MBP-tag was not successful. We therefore wanted to test whether the arginases FlmR and OhkR would be soluble (and stable) without the MBP-tag. His_6-tagged peptide arginase constructs were cloned by deleting the MBP-tag from the newly cloned pCDF-MBPopt-*ohkR* and pCDF-MBPopt-*flmR* and religating the plasmid. This was achieved by PCR amplification of each plasmid with P_delMBP_f and P_delMBP_r (Table S1), treatment of the PCR product with KLD enzyme mix (New England Biolabs) and transformation of electro- or chemo-competent *E. coli* DH5α cells with the mix. The resulting cell suspension was plated on LB agar plates containing streptomycin. Plasmid isolation of the obtained colonies was conducted with Promega Wizard® Plus SV Minipreps DNA Purification System, the resulting plasmids pCDF-His-*ohkR* (Sm^R) and pCDF-His-*flmR* (Sm^R) were verified by enzymatic digest and sequencing (Eurofins Genomics, Germany).

Cloning of precursor variants

To investigate the role of the ‘DD(I/V)LF’ motif, the precursor variants FlmA1-D53A-I54A-L55A-F56A and OhkA1-D66A-D67A-V68A-L69A-F70A were constructed. For this purpose, the respective template plasmids pET28a-*flmA1* (Kan^R) or pET28a-*ohkA1* (Kan^R) were amplified by PCR with the primer pairs P_FlmA1_D53A-I54A-L55A-F56A_f / P_FlmA1_D53A-I54A-L55A-F56A_r and P_OhkA1_D66A-D67A-V68A-L69A-F70A-f / P_OhkA1_D66A-D67A-V68A-L69A-F70A-r, respectively (Table S1). Treatment with KLD enzyme mix (New England Biolabs), transformation of *E. coli* XL1-Blue cells with the resulting mixture, and verification of the final plasmids were carried out as described above. Similarly, the precursor variants OhkA1$^{16-70}$ and OhkA2$^{18-69}$ were constructed by amplifying pET28a-*ohkA1* (Kan^R) and pET28a-*ohkA2* (Kan^R) with primer pairs P_OhkA1_16-70_f / P_OhkA1_16-70_r and P_OhkA2_18-69_f / P_OhkA2_18-69_r (Table S1), respectively, and by then following the same procedure.

Table S1. Primer list.

Name	Sequence 5' → 3'
P_FlmR_FseI_f	TATATAGGCCGGCCATGAAAAAAACTTCAATTCAAACC
P_FlmR_XhoI_r	CGCGCGCTCGAGCTAAGCAATTTCATTTTGAAAAAAATTG
P_FlmA1_NdeI_f	CGCGCGCATATGGGAAAAAGAAAAAAAATTAAAGAG
P_FlmA1_HindIII_r	CGCGCGAAGCTTAAAATAATATGTCATTTTCTTC
P_FlmA2_NdeI_f	CGCGCGCATATGAAAAACAAGAAATTGAAAGC
P_FlmA2_HindIII_r	GCGCGCAAGCTTAAAATAAAATATCACTTTCCTCATC
P_FlmA3_NdeI_f	CGCGCGCATATGAAAAAAGAGAGACAAATTCC
P_FlmA3_HindIII_r	CGCGCGAAGCTTAAAAAAGAATATCATCTGCTTC
P_FlmA1_D53A-I54A-L55A-F56A_f	GCCGCGTAAGCTTGCGGCCGCACTC
P_FlmA1_D53A-I54A-L55A-F56A_r	TGCCGCATTTTCTTCATTCATACCATAGTTAACAC
P_OhkR_NdeI_f	TATACGCATATGATGCGCGATAAAACTATTCC
P_OhkR_XhoI_r	CGCGCGCTCGAGTTAATTATTATATATACCACCCACG
P_OhkA1_NdeI_f	TACGCGCATATGAAAAACAAAGAAGCCTCC
P_OhkA1_HindIII_r	TACGCGAAGCTTAGAAGAGGACGTCATCATTG
P_OhkA2_NdeI_f	CGCGCGCATATGGAAAAGAATGAAAAATTTCCC
P_OhkA2_HindIII_r	CGCGCGAAGCTTAAAATAAAACGTCGTCGTTG
P_OhkA1_D66A-D67A-V68A-L69A-F70A-f	AGCCGCGTAAGCTTGCGGCCGCACTC
P_OhkA1_D66A-D67A-V68A-L69A-F70A-r	GCGGCCGCATTGTCCCCTGTGGAGCTGAAG
P_OhkA1_16-70_f	TCGAACAAAGCCCCCAAAAC
P_OhkA1_16-70_r	ATGGCCCTGAAAATAAAGATTCTC
P_OhkA2_18-69_f	AGCAAAAAGACCCTGGAGGG
P_OhkA2_18-69_r	ATGGCCCTGAAAATAAAGATTCTC
P_MBP_Mutag_FW	ATCGAGAACCTGTACTTCC
P_MBP_Mutag_RV	CCCGAGGTTGTTGTTATTG
P_FlmR_FW	TATATACATATGAAAAAAACTTCAATTCAAAC
P_FlmR_RV	AAAAAACTCGAGCTAAGCAATTTC
P_delMBP_f	GAGAACCTGTACTTCCAGTC
P_delMBP_r	GTGGTGATGATGGTGATGG

DNA Sequences

Start and stop codons are underlined, His-tags are shown in grey.

>FlmR (WP_106143370)

ATGGGCAGCAGCCATCACCATCATCACCACAGCCAGGATCCGAAAATCGAAGAAGGTAAACTGGTAATCTGGATTAACGGCGATAAAGGCTATAACGG
TCTCGCTGAAGTCGGTAAGAAATTCGAGAAAGATACCGGAATTAAAGTCACCGTTGAGCATCCGGATAAACTGGAAGAGAAATTCCCACAGGTTGCGG
CAACTGGCGATGGCCCTGACATTATCTTCTGGGCACACGACCGCTTTGGTGGCTACGCTCAATCTGGCCTGTTGGCTGAAATCACCCCGGACAAAGCG
TTCCAGGACAAGCTGTATCCGTTTACCTGGGATGCCGTACGTTACAACGGCAAGCTGATTGCTTACCCGATCGCTGTTGAAGCGTTATCGCTGATTTA
TAACAAAGATCTGCTGCCGAACCCGCCAAAAACCTGGGAAGAGATCCCGGCGCTGGATAAAGAACTGAAAGCGAAAGGTAAGAGCGCGCTGATGTTCA
ACCTGCAAGAACCGTACTTCACCTGGCCGCTGATTGCTGCTGACGGGGGTTATGCGTTCAAGTATGAAAACGGCAAGTACGACATTAAAGACGTGGGC
GTGGATAACGCTGGCGCGAAAGCGGGTCTGACCTTCCTGGTTGACCTGATTAAAAACAAACACATGAATGCAGACACCGATTACTCCATCGCAGAAGC
TGCCTTTAATAAAGGCGAAACAGCGATGACCATCAACGGCCCGTGGGCATGGTCCAACATCGACACCAGCAAAGTGAATTATGGTGTAACGGTACTGC
CGACCTTCAAGGGTCAACCATCCAAACCGTTCGTTGGCGTGCTGAGCGCAGGTATTAACGCCGCCAGTCCGAACAAAGAGCTGGCAAAAGAGTTCCTC
GAAAACTATCTGCTGACTGATGAAGGTCTGGAAGCGGTTAATAAAGACAAACCGCTGGGTGCCGTAGCGCTGAAGTCTTACGAGGAAGAGTTGGCGAA
AGATCCACGTATTGCCGCCACTATGGAAAACGCCCAGAAAGGTGAAATCATGCCGAACATCCCGCAGATGTCCGCTTTCTGGTATGCCGTGCGTACTG
CGGTGATCAACGCCGCCAGCGGTCGTCAGACTGTCGATGAAGCCCTGAAAGACGCGCAGACTAATTCGAGCTCGAACAACAACAACAATAACAATAAC
AACAACCTCGGGATCGAGAACCTGTACTTCCAGTCACATATGAAAAAAACTTCAATTCAAACCTTTGTTATAGAAGAACATAATGAAGCGTTTTTCGT
ATGGCAATATGCCCTAGCTAATGGACTTATTAAACAAAAGCAAAATTGCTTAATTCATGTTGATGAGCATTCAGACATGGGAACTCCAAGGTTTAATA
TTTCTATAGACAATTTAGGTTCAGATTTATCCGATATTCATGATTTTGTATTTAAGGAGCTAAACATTGCCTCTTTTATTATACCCTCAATTTATAAA
GGTTTATTCCGAAGTATTTATTGGGTTAAACAAAAACATAGAAAATCTTCAATCACTTCAAATAAAATGTATGTCAAATCCTTAAATGATTCCGGTAA
GAAATTGATGTCTGGTAAGATTGAAGATATACCTAAGATTATCGAGGAGTACAGAAAGTCCAGTACAACATACAAAGAATTTGAGTATCACCTTGTTA
CCGAAGAAAACCTACCATCTAAAGTAAATCCGGTATTAGACATAGACTTGGACTATTTTTCCTGTACAGGAATGCCTAATCAAAACAAGGAAATAATA
ATTGAGATATCTGAAGATGAGTTTAATGAGTTTACAAATGACAAATACCACCGTTTGAATTATTTACCATCTAGGGTAGAAGCTAGAAAAATTGATGG
AAAATATTTCTATCTCTTAAATTATTATAATGAGTTATATCCAAGTAGTCTTTTAAAAAACAAGAAGGAGATTGAAGAAAGAGTTAAAAAGTTTGTTA
ACACAATCTCCAAAAAAGTAGGTGAACCTCAAATAATAACTATCTGTAGATCACGATTTAGTGAATACACTCCTCCAGAGCAATGGGAATTCATTGAA
AAATATCTAATAAGTTGTCTATCAGAAATTTACAATTTAGATATCCACTACATAGACGAAATCAATTTTTTTCAAAATGAAATTGCTTAG

>OhkR (WP_159453773)

ATGGGCAGCAGCCATCACCATCATCACCACAGCCAGGATCCGAAAATCGAAGAAGGTAAACTGGTAATCTGGATTAACGGCGATAAAGGCTATAACGG
TCTCGCTGAAGTCGGTAAGAAATTCGAGAAAGATACCGGAATTAAAGTCACCGTTGAGCATCCGGATAAACTGGAAGAGAAATTCCCACAGGTTGCGG
CAACTGGCGATGGCCCTGACATTATCTTCTGGGCACACGACCGCTTTGGTGGCTACGCTCAATCTGGCCTGTTGGCTGAAATCACCCCGGACAAAGCG
TTCCAGGACAAGCTGTATCCGTTTACCTGGGATGCCGTACGTTACAACGGCAAGCTGATTGCTTACCCGATCGCTGTTGAAGCGTTATCGCTGATTTA
TAACAAAGATCTGCTGCCGAACCCGCCAAAAACCTGGGAAGAGATCCCGGCGCTGGATAAAGAACTGAAAGCGAAAGGTAAGAGCGCGCTGATGTTCA
ACCTGCAAGAACCGTACTTCACCTGGCCGCTGATTGCTGCTGACGGGGGTTATGCGTTCAAGTATGAAAACGGCAAGTACGACATTAAAGACGTGGGC
GTGGATAACGCTGGCGCGAAAGCGGGTCTGACCTTCCTGGTTGACCTGATTAAAAACAAACACATGAATGCAGACACCGATTACTCCATCGCAGAAGC
TGCCTTTAATAAAGGCGAAACAGCGATGACCATCAACGGCCCGTGGGCATGGTCCAACATCGACACCAGCAAAGTGAATTATGGTGTAACGGTACTGC
CGACCTTCAAGGGTCAACCATCCAAACCGTTCGTTGGCGTGCTGAGCGCAGGTATTAACGCCGCCAGTCCGAACAAAGAGCTGGCAAAAGAGTTCCTC
GAAAACTATCTGCTGACTGATGAAGGTCTGGAAGCGGTTAATAAAGACAAACCGCTGGGTGCCGTAGCGCTGAAGTCTTACGAGGAAGAGTTGGCGAA
AGATCCACGTATTGCCGCCACTATGGAAAACGCCCAGAAAGGTGAAATCATGCCGAACATCCCGCAGATGTCCGCTTTCTGGTATGCCGTGCGTACTG
CGGTGATCAACGCCGCCAGCGGTCGTCAGACTGTCGATGAAGCCCTGAAAGACGCGCAGACTAATTCGAGCTCGAACAACAACAACAATAACAATAAC
AACAACCTCGGGATCGAGAACCTGTACTTCCAGTCACATATGATGCGCGATAAAACTATTCCATTATTCACCGTTGAAGAACACCATGAAGCTTTCTT
TGTATGGAAGCATGCACTGCTTCATAAGCTGATTCGTGGGCGGGATCATGCGCTTCTCCACGTGGATGAGCACTCTGACATGGGCGCCCCAACGCTCA
ACAATTCCATCCACTTGTTAAACGGGAATCTTAAGCGGGTCCAACAATTCACCCATCAGGAACTCACCATCGCTAACTTTATTATCCCGGCCATTTAT
GAAGGCCTGTTTAAAAAGGTATACTGGGTTAAGCAAAGACATAACAAAACCAACAGCCGGGCGATGCATTTGTATGTACGTTCATCCAATGGAGAAGG
CAAGAAGCTGGTGACCGGCAAGATGTCTGTTCTGGAAGAACATGGTAAAGATCCCGAGGCGTTGGGAGATGGCGCTGTGATCTTTAAATTTTACAAAC
AACATGTTGAACAGCTTACGACAATCAAGGACGTTATGCTCGACATCGACCTGGACTATTTTTCGTGCATACAAAATCCTTTGAAACGTGAATTGAGA
ATTGAAATCACGCGTGAGGAGTATACCGCCTTTCTAAATACACCTTACCATCGACTGCGTTTCTTTGATTTCGGCCGCGTAGAAGCCCGCAAGATCGG
TCAACGTTATTATTATTATCTAAACAGCTTTAAAGAACAGTATGATTCTCCTTTGAAAGTATCCGAAGCCTTAATACAATCTCGTGTGGATGATTTCA
TCCAGGCACTTGCTGTAAATAAAATTAAACCGCTTCTGGTTACGGTGTGTCGTTCACGGTATAGCGGATATACCCCCGTGGATCAATGGAATTTTATA
GAGCAATCATTGCTGCGGGGCCTCCATAGCCTCTATGGTAAAATGTCGGTGCAACACGTGGGTGGTATATATAATAATTAA

>His_6-FlmA1 (WP_106143368)

ATGGGCAGCAGCCATCATCATCATCATCACAGCAGCGGCGAGAATCTTTATTTTCAGGGCCATATGGGAAAAAGAAAAAAATTAAAGAGTTTACCAAA
AGAACTAATTAAGCCAGAGAAAACAGATACATTTTTTACAACTCAAATGGTTGAGTTTATGTGCCGAGAAAATGATCACGCAACTACTTGCCGTGTTA
ACTATGGTATGAATGAAGAAAATGACATATTATTTTAA

>His_6-FlmA2

ATGGGCAGCAGCCATCATCATCATCATCACAGCAGCGGCGAGAATCTTTATTTTCAGGGCCATATGAAAAACAAGAAATTGAAAGCATTGCCTAAAGA
ATTGGTTAAACCAACAGAGATTGACCTCAACACTTCAGGAGATTCAGTAGAATTTTTATGTAGGGAGTTCGGTAGTGGAACCACTTGCAGAATCAACG
GAGCTATTGATGAGGAAAGTGATATTTTATTTTAA

>His$_6$-FlmA3
ATGGGCAGCAGCCATCATCATCATCATCACAGCAGCGGCGAGAATCTTTATTTTCAGGGCCATATGAAAAAAGAGAGACAAATTCCAAAGTTGCCAAA
GGAGTTAATTAAACCTTCAGAAATTGATACCGAGGCAAAAGGAGGCGCAGTTGAATTCCTTTGTAATGAGTTTGGTAACGGAACAACCTGCCGGATTA
ATCGGGGGATAGATGAAGCAGATGATATTCTTTTTTAG

>His$_6$-OhkA1 (WP_079688958)
ATGGGCAGCAGCCATCATCATCATCATCACAGCAGCGGCGAGAATCTTTATTTTCAGGGCCATATGAAAAACAAAGAAGCCTCCAAGACCAAGCAGCT
CTTGGAGCCCTCGAACAAAGCCCCCAAAACTATTGACGGGGTCGAAGTTTCCTTGGAGCAACTCGAATCACTGTGTACAGAGTTCGACAAGCGACCAC
GCGGGTGTAATTCCGAGACTGAACCGGCAGATGGAGCTGGCTTCAGCTCCACAGGGGACAATGATGACGTCCTCTTCTAA

>His$_6$-OhkA2 (WP_079688957)
ATGGGCAGCAGCCATCATCATCATCATCACAGCAGCGGCGAGAATCTTTATTTTCAGGGCCATATGGAAAAGAATGAAAAATTTCCCAAGCAAATCCT
AGAACCTTCAACACGAAGCAAAAAGACCCTGGAGGGAAAAGATGTTTCTCTGGAAGAGCTAGAATCACTCTGCAGTAATTTTGATAAACGTCCCAGAG
GTTGCAACTCTGAAACAGAGCCTGCTGATGGTGCCGGTTTCTCATCGACCGGTGACAACGACGACGTTTTATTTTAA

>His$_6$-FlmA1-D53A-I54A-L55A-F56A
ATGGGCAGCAGCCATCATCATCATCATCACAGCAGCGGCGAGAATCTTTATTTTCAGGGCCATATGGGAAAAAGAAAAAAATTAAAGAGTTTACCAAA
AGAACTAATTAAGCCAGAGAAAACAGATACATTTTTTACAACTCAAATGGTTGAGTTTATGTGCCGAGAAAATGATCACGCAACTACTTGCCGTGTTA
ACTATGGTATGAATGAAGAAAATGCGGCAGCCGCGTAA

>His$_6$-OhkA1-D66A-D67A-V68A-L69A-F70A
ATGGGCAGCAGCCATCATCATCATCATCACAGCAGCGGCGAGAATCTTTATTTTCAGGGCCATATGAAAAACAAAGAAGCCTCCAAGACCAAGCAGCT
CTTGGAGCCCTCGAACAAAGCCCCCAAAACTATTGACGGGGTCGAAGTTTCCTTGGAGCAACTCGAATCACTGTGTACAGAGTTCGACAAGCGACCAC
GCGGGTGTAATTCCGAGACTGAACCGGCAGATGGAGCTGGCTTCAGCTCCACAGGGGACAATGCGGCCGCAGCCGCGTAA

>His$_6$-OhkA1$^{16-70}$
ATGGGCAGCAGCCATCATCATCATCATCACAGCAGCGGCGAGAATCTTTATTTTCAGGGCCATTCGAACAAAGCCCCCAAAACTATTGACGGGGTCGA
AGTTTCCTTGGAGCAACTCGAATCACTGTGTACAGAGTTCGACAAGCGACCACGCGGGTGTAATTCCGAGACTGAACCGGCAGATGGAGCTGGCTTCA
GCTCCACAGGGGACAATGATGACGTCCTCTTCTAA

>His$_6$-OhkA2$^{18-69}$
ATGGGCAGCAGCCATCATCATCATCATCACAGCAGCGGCGAGAATCTTTATTTTCAGGGCCATAGCAAAAAGACCCTGGAGGGAAAAGATGTTTCTCT
GGAAGAGCTAGAATCACTCTGCAGTAATTTTGATAAACGTCCCAGAGGTTGCAACTCTGAAACAGAGCCTGCTGATGGTGCCGGTTTCTCATCGACCG
GTGACAACGACGACGTTTTATTTTAA

Protein Sequences
His$_6$-tags are underlined, MBP-tags are shown in grey.

>FlmR (WP_106143370)
MKKTSIQTFVIEEHNEAFFVWQYALANGLIKQKQNCLIHVDEHSDMGTPRFNISIDNLGSDLSDIHDFVFKELNIASFIIPSIYKGLFRSIYWVKQKH
RKSSITSNKMYVKSLNDSGKKLMSGKIEDIPKIIEEYRKSSTTYKEFEYHLVTEENLPSKVNPVLDIDLDYFSCTGMPNQNKEIIIEISEDEFNEFTN
DKYHRLNYLPSRVEARKIDGKYFYLLNYYNELYPSSLLKNKKEIEERVKKFVNTISKKVGEPQIITICRSRFSEYTPPEQWEFIEKYLISCLSEIYNL
DIHYIDEINFFQNEIA

>His$_6$-FlmR
MGSSHHHHHHENLYFQSHMKKTSIQTFVIEEHNEAFFVWQYALANGLIKQKQNCLIHVDEHSDMGTPRFNISIDNLGSDLSDIHDFVFKELNIASFII
PSIYKGLFRSIYWVKQKHRKSSITSNKMYVKSLNDSGKKLMSGKIEDIPKIIEEYRKSSTTYKEFEYHLVTEENLPSKVNPVLDIDLDYFSCTGMPNQ
NKEIIIEISEDEFNEFTNDKYHRLNYLPSRVEARKIDGKYFYLLNYYNELYPSSLLKNKKEIEERVKKFVNTISKKVGEPQIITICRSRFSEYTPPEQ
WEFIEKYLISCLSEIYNLDIHYIDEINFFQNEIA

>His$_6$-MBP-FlmR
MGSSHHHHHHSQDPKIEEGKLVIWINGDKGYNGLAEVGKKFEKDTGIKVTVEHPDKLEEKFPQVAATGDGPDIIFWAHDRFGGYAQSGLLAEITPDKA
FQDKLYPFTWDAVRYNGKLIAYPIAVEALSLIYNKDLLPNPPKTWEEIPALDKELKAKGKSALMFNLQEPYFTWPLIAADGGYAFKYENGKYDIKDVG
VDNAGAKAGLTFLVDLIKNKHMNADTDYSIAEAAFNKGETAMTINGPWAWSNIDTSKVNYGVTVLPTFKGQPSKPFVGVLSAGINAASPNKELAKEFL
ENYLLTDEGLEAVNKDKPLGAVALKSYEEELAKDPRIAATMENAQKGEIMPNIPQMSAFWYAVRTAVINAASGRQTVDEALKDAQTNSSSNNNNNNNN
NNLGIENLYFQSHMKKTSIQTFVIEEHNEAFFVWQYALANGLIKQKQNCLIHVDEHSDMGTPRFNISIDNLGSDLSDIHDFVFKELNIASFIIPSIYK
GLFRSIYWVKQKHRKSSITSNKMYVKSLNDSGKKLMSGKIEDIPKIIEEYRKSSTTYKEFEYHLVTEENLPSKVNPVLDIDLDYFSCTGMPNQNKEII
IEISEDEFNEFTNDKYHRLNYLPSRVEARKIDGKYFYLLNYYNELYPSSLLKNKKEIEERVKKFVNTISKKVGEPQIITICRSRFSEYTPPEQWEFIE
KYLISCLSEIYNLDIHYIDEINFFQNEIA

>OhkR (WP_159453773)

MMRDKTIPLFTVEEHHEAFFVWKHALLHKLIRGRDHALLHVDEHSDMGAPTLNNSIHLLNGNLKRVQQFTHQELTIANFIIPAIYEGLFKKVYWVKQR
HNKTNSRAMHLYVRSSNGEGKKLVTGKMSVLEEHGKDPEALGDGAVIFKFYKQHVEQLTTIKDVMLDIDLDYFSCIQNPLKRELRIEITREEYTAFLN
TPYHRLRFFDFGRVEARKIGQRYYYYLNSFKEQYDSPLKVSEALIQSRVDDFIQALAVNKIKPLLVTVCRSRYSGYTPVDQWNFIEQSLLRGLHSLYG
KMSVQHVGGIYNN

> His_6-OhkR

MGSSHHHHHHENLYFQSHMMRDKTIPLFTVEEHHEAFFVWKHALLHKLIRGRDHALLHVDEHSDMGAPTLNNSIHLLNGNLKRVQQFTHQELTIANFI
IPAIYEGLFKKVYWVKQRHNKTNSRAMHLYVRSSNGEGKKLVTGKMSVLEEHGKDPEALGDGAVIFKFYKQHVEQLTTIKDVMLDIDLDYFSCIQNPL
KRELRIEITREEYTAFLNTPYHRLRFFDFGRVEARKIGQRYYYYLNSFKEQYDSPLKVSEALIQSRVDDFIQALAVNKIKPLLVTVCRSRYSGYTPVD
QWNFIEQSLLRGLHSLYGKMSVQHVGGIYNN

> His_6-MBP-OhkR

MGSSHHHHHHSQDPKIEEGKLVIWINGDKGYNGLAEVGKKFEKDTGIKVTVEHPDKLEEKFPQVAATGDGPDIIFWAHDRFGGYAQSGLLAEITPDKA
FQDKLYPFTWDAVRYNGKLIAYPIAVEALSLIYNKDLLPNPPKTWEEIPALDKELKAKGKSALMFNLQEPYFTWPLIAADGGYAFKYENGKYDIKDVG
VDNAGAKAGLTFLVDLIKNKHMNADTDYSIAEAAFNKGETAMTINGPWAWSNIDTSKVNYGVTVLPTFKGQPSKPFVGVLSAGINAASPNKELAKEFL
ENYLLTDEGLEAVNKDKPLGAVALKSYEEELAKDPRIAATMENAQKGEIMPNIPQMSAFWYAVRTAVINAASGRQTVDEALKDAQTNSSSNNNNNNNN
NNLGIENLYFQSHMMRDKTIPLFTVEEHHEAFFVWKHALLHKLIRGRDHALLHVDEHSDMGAPTLNNSIHLLNGNLKRVQQFTHQELTIANFIIPAIY
EGLFKKVYWVKQRHNKTNSRAMHLYVRSSNGEGKKLVTGKMSVLEEHGKDPEALGDGAVIFKFYKQHVEQLTTIKDVMLDIDLDYFSCIQNPLKRELR
IEITREEYTAFLNTPYHRLRFFDFGRVEARKIGQRYYYYLNSFKEQYDSPLKVSEALIQSRVDDFIQALAVNKIKPLLVTVCRSRYSGYTPVDQWNFI
EQSLLRGLHSLYGKMSVQHVGGIYNN

> His_6-FlmA1 (WP_106143368)

MGSSHHHHHHSSGENLYFQGHMGKRKKLKSLPKELIKPEKTDTFFTTQMVEFMCRENDHATTCRVNYGMNEENDILF

> His_6-FlmA2

MGSSHHHHHHSSGENLYFQGHMKNKKLKALPKELVKPTEIDLNTSGDSVEFLCREFGSGTTCRINGAIDEESDILF

> His_6-FlmA3

MGSSHHHHHHSSGENLYFQGHMKKERQIPKLPKELIKPSEIDTEAKGGAVEFLCNEFGNGTTCRINRGIDEADDILF

> His_6-OhkA1 (WP_079688958)

MGSSHHHHHHSSGENLYFQGHMKNKEASKTKQLLEPSNKAPKTIDGVEVSLEQLESLCTEFDKRPRGCNSETEPADGAGFSSTGDNDDVLF

> His_6-OhkA2 (WP_079688957)

MGSSHHHHHHSSGENLYFQGHMEKNEKFPKQILEPSTRSKKTLEGKDVSLEELESLCSNFDKRPRGCNSETEPADGAGFSSTGDNDDVLF

> His_6-FlmA1-D53A-I54A-L55A-F56A

MGSSHHHHHHSSGENLYFQGHMGKRKKLKSLPKELIKPEKTDTFFTTQMVEFMCRENDHATTCRVNYGMNEENAAAA

> His_6-OhkA1-D66A-D67A-V68A-L69A-F70A

MGSSHHHHHHSSGENLYFQGHMKNKEASKTKQLLEPSNKAPKTIDGVEVSLEQLESLCTEFDKRPRGCNSETEPADGAGFSSTGDNAAAAA

> His_6-OhkA1$^{16-70}$

MGSSHHHHHHSSGENLYFQGHSNKAPKTIDGVEVSLEQLESLCTEFDKRPRGCNSETEPADGAGFSSTGDNDDVLF

> His_6-OhkA2$^{18-69}$

MGSSHHHHHHSSGENLYFQGHSKKTLEGKDVSLEELESLCSNFDKRPRGCNSETEPADGAGFSSTGDNDDVLF

(Co-)Expression, Precursor and Arginase Purification, and Peptide Digests

E. coli BL21(DE3) was used as protein production strain. The production of Flm precursors alone was performed in *E. coli* BL21(DE3) pLysS due to higher yields. Electrocompetent cells were prepared in-house and transformed with the expression constructs described above. For overnight cultures 5 to 10 mL LB medium, supplemented with the appropriate antibiotics, was inoculated with a single colony and grown at 37°C for ~16 h. For small scale production, 100 mL LB medium containing the appropriate antibiotic(s) was inoculated 1:100 with overnight culture and grown at 37°C and 200 rpm until an OD_{600} ~0.5-0.8 was reached. Large scale production was performed similarly with 1 L TB medium, incubation at 37°C and 160 rpm until OD_{600} ~1.5-2.0. Cultures were cooled down and induced with 1 mM IPTG (final concentration). Expression was performed at 18°C, 160-200 rpm for 12-24 h. Cells were harvested by centrifugation at 4°C, 4.000 rpm for 20 min and stored at –70°C until further use or directly resuspended in lysis buffer.

For protein purification, cells were resuspended in lysis buffer (50 mM sodium phosphate pH 8.0, 300 mM NaCl, 10% glycerol, 20 mM imidazole). Per 4 g cell pellet 5 mL lysis buffer was added. Cell lysis was either performed by sonication on ice (small scale: 30% amplitude, 2 min, 10 sec on/10 sec off; large scale: 40% amplitude, 8 min, 10 sec on/10 sec off) or by using a French Press. The cleared lysate was passed twice over a Ni^{2+}-affinity chromatography (Ni-NTA Agarose, Qiagen GmbH) gravity column. After washing steps, the His-tagged proteins were eluted by a high imidazole concentration buffer (50 mM sodium phosphate pH 8.0, 300 mM NaCl, 10% glycerol, 250 mM imidazole). Proteins were subsequently desalted by buffer exchange (50 mM TRIS pH 8.0, 300 mM NaCl, 10% glycerol) and concentrated by Amicon centrifugal filters (MWCO 3/10/30/50 kDa; Merck KGaA). Purity of purified protein samples was checked by SDS-PAGE. Protein concentration was determined by Nanodrop using the sample type "E1%". The theoretical value of the mass extinction coefficient was entered for each peptide or protein. Average protein yields are listed in Table S7.

For endoproteinase digestion, ~5 µg of precursor was mixed with 10 µL reaction buffer (according to manufacturer´s protocol), 1 mM DTT, and 0.01 µg LysC or 0.1 µg GluC, and adjusted to 20 µL total reaction volume with MilliQ water. After incubation at 37°C for up to 24 h, the digests were diluted with 20 µL of 0.1% formic acid, centrifuged at 13.200 rpm for 1 h and analysed by HPLC-MS/MS^2.

For the formic acid digest, ~5-10 µg of precursor was mixed with 1 mM DTT and 10 µL 4%/10% aqueous formic acid solution and adjusted to 20 µL total reaction volume with MilliQ water. After incubation at 100°C for 1h, the digest samples were centrifuged at 13.200 rpm for 1 h and analysed by HPLC-MS/MS^2.

HPLC-MS/MS Analysis

Liquid chromatography–mass spectrometry analyses were performed using a Vanquish UHPLC system coupled to a Q Exactive HF quadrupole-orbitrap mass spectrometer (Thermo Fisher Scientific, Bremen, Germany), equipped with a heated electrospray ionization (HESI) source. Chromatographic separation was achieved using a Kinetex EVO C18 reversed-phase column (50 × 2.1 mm, 1.7 µm particle size, 100 Å pore size; Phenomenex, Torrance, USA). The mobile phases consisted of water + 0.1% formic acid (solvent A) and acetonitrile + 0.1% formic acid (solvent B), both Optima™ LC-MS grade (Fisher Scientific, San Diego, USA). The flow rate was set to 0.5 mL/min. The gradient elution program was as follows: 0–0.5 min, 5% B; 0.5–8 min, linear increase to 50% B; 8–10 min, linear increase to 99% B; followed by a 2 min washout phase at 99% B and a 3 min re-equilibration phase at 5% B. An injection volume of 5 µL was used for all samples.

For untargeted analysis, data-dependent acquisition (DDA) was performed in positive ion mode, selecting the top 5 most intense precursor ions in each MS1 scan for fragmentation. Full MS scans (MS1) were acquired at a resolution of 30,000, and MS/MS scans (MS^2) at a resolution of 15,000. A stepped normalized collision energy (NCE) of 20, 25, and 30 was applied.

For targeted analysis, parallel reaction monitoring (PRM) was performed using predefined inclusion lists containing specific precursor masses. Collision energies (CE) were adjusted as needed, ranging from 10 to 35 eV. The same chromatographic conditions described above were applied for PRM runs.

Enzyme Activity Assays/Kinetic Analyses

In vitro peptide arginase activity was measured with a spectrophotometric assay using the QuantiChrom™ Urea Assay Kit from BioAssay Systems in accordance with the manufacturer's protocol. This kit necessitates an endpoint assay, hence ethylenediaminetetraacetate (EDTA) was utilised to stop the enzymatic reaction (50 mM EDTA final concentration).

The kinetic parameters of FlmR and OhkR were determined in 450 µL reaction mixtures containing 50 mM TRIS pH 8.5, 1 mM $MnCl_2$, 1 mM DTT, and 0.5–250 µM substrate for FlmA1 and 0.1–250 µM for OhkA1. The reactions were initiated by addition of the peptide arginase at a concentration of 10 µM (FlmR or OhkR) At selected time points, 50 µL aliquots were withdrawn, and the reaction was quenched by addition of EDTA. Subsequent to this, samples were briefly centrifuged and stored at –20 °C until spectrophotometric analysis. Prior to analysis, precipitated protein was removed by centrifugation (12,044 × g, 5 min) and the supernatant was transferred to a 96-well plate. After the addition of the QuantiChrom™ reagents, samples were incubated at 25 °C for 50 min. The absorbances were measured with a Tecan Infinite 200 Pro (Plex) spectrophotometer at 430 nm and 25 °C after 15 s of orbital shaking to determine the concentration of the coproduct, urea. All assays were performed in triplicate. Relative rates were measured accordingly, with substrate concentrations of 70 µM substrate (for FlmR) and 1 µM substrate (for OhkR).

The time course of urea formation was fitted to a linear function to give the initial rates of reaction, which were then used to calculate specific activity and k_{cat} values. The analysis was performed with Origin(Pro) using "non-linear fit" analysis with "MichaelisMenten" function.[3]

Statistical Analysis

For determination of FlmR and OhkR kinetics and relative rates, biological triplicates and technical duplicates were measured. The urea concentration of the samples was calculated according to the manufacturer's protocol. A urea standard provided by the manufacturer was included in each measurement and used to normalize the data. Outliers were detected using the sorting method. The velocity at each substrate concentration is given as mean ± standard deviation. Michaelis-Menten curves were generated with Origin(Pro) using "non-linear fit" analysis with "MichaelisMenten" function.[3]

AlphaFold models were predicted five times independently with default settings, resulting in at least three highly similar models. The model with the highest confidence values was used for further analysis. The pLDDT value gives a per-atom confidence, indicating a higher confidence at higher values.[4] To determine the pLDDT value of the entire model, the mean of the "atom_plddts" values given by AlphaFold was calculated.

Results and Discussion

Bioinformatic Analysis of Genetic Region Surrounding the *flm* and *ohk* Peptide Arginases

Table S2. Genes of the postulated genetic locus harbouring the *flm* BGC as displayed in Figure 1 (main text), including the respective protein products, the proposed function, and the closest BLAST homologue. AA = amino acids.

Gene	NCBI annotation [*Flagellimonas meridianipacifica*]	Accession	AA	Protein homologue (BLAST)	Identity [%]	Proposed function
orf1	hypothetical protein	WP_106143363.1	76	-	-	unknown function
orf2	ABC transporter permease	WP_146129809.1	787	FtsX-like permease family protein [*Flagellimonas sediminis*]	48.70	ABC transporter permease
flmB	UpxY family transcription antiterminator	WP_106143365.1	176	UpxY family transcription antiterminator [*Flavobacterium humi*]	37.11	regulation
flmC	2OG-Fe(II) oxygenase	WP_106143366.1	196	2OG-Fe(II) oxygenase [*Sinomicrobium oceani*]	58.47	hydroxylase
flmD	AraC-like DNA-binding protein	WP_106143367.1	335	helix-turn-helix domain-containing protein [*Cytophagales bacterium*]	34.35	regulator
flmA1	hypothetical protein	WP_158259055.1	56	hypothetical protein (*flmA2*) [*Flagellimonas meridianipacifica*]	55.56	precursor peptide
flmA2	hypothetical protein	WP_158259056.1	55	hypothetical protein (*flmA3*) [*Flagellimonas meridianipacifica*]	62.96	precursor peptide
flmA3	hypothetical protein	WP_158259057.1	56	hypothetical protein (*flmA2*) [*Flagellimonas meridianipacifica*]	62.96	precursor peptide
flmE	radical SAM/SPASM domain-containing protein	WP_106143369.1	486	radical SAM/SPASM domain-containing protein [*Chitinophaga hostae*]	53.70	radical SAM/SPASM domain-containing protein
flmR	UPF0489 family protein	WP_106143370.1	310	UPF0489 family protein [*Pedobacter lusitanus*]	48.32	peptide arginase
flmF	S41 family peptidase	WP_106143371.1	653	S41 family peptidase [*Cytophagales bacterium*]	30.09	peptidase
flmG	hypothetical protein	WP_106143372.1	293	hypothetical protein [uncultured *Chitinophaga* sp.]	34.26	unknown function
flmH	S8 family serine peptidase	WP_106143373.1	638	S8 family peptidase [*Maribacter flavus*]	41.04	serine peptidase
flmI	cupin-like domain-containing protein	WP_106143374.1	282	cupin-like domain-containing protein [*Maribacter flavus*]	48.64	cupin domain-containing protein

Gene	NCBI annotation [*Flagellimonas meridianipacifica*]	Accession	AA	Protein homologue (BLAST)	Identity [%]	Proposed function
flmJ	hypothetical protein	WP_106143375.1	87	acyl carrier protein [*Bacteroidales bacterium*]	56.00	unknown function
flmK	holo-ACP synthase	WP_106143376.1	122	holo-ACP synthase [Candidatus *Acidiferrum* sp.]	48.76	holo-ACP synthase
flmL	ABC transporter ATP-binding protein	WP_158259058.1	535	ABC transporter ATP-binding protein [*Sinomicrobium oceani*]	48.83	ABC transporter permease
orf3	condensation domain-containing protein	WP_106143378.1	446	condensation domain-containing protein [*Puia* sp.]	27.73	unknown function

Table S3. Genes of the postulated genetic locus harboring the *ohk* BGC as displayed in Figure 1 (main text), including the respective protein products, the proposed function, and the closest BLAST homologue. AA = amino acids.

Gene	NCBI annotation [*Ohtaekwangia koreensis*]	Accession	AA	Protein homolog (BLAST)	Identity [%]	Proposed function
orf1	hypothetical protein	WP_079688964.1	143	-	-	unknown function
orf2	ParB N-terminal domain-containing protein	WP_079688963.1	304	ParB N-terminal domain-containing protein [*Chryseosolibacter histidini*]	60.00	unknown function
orf3	alpha/beta hydrolase family protein	WP_079688962.1	776	S9 family peptidase [*Bacteroidota bacterium*]	42.64	alpha/beta hydrolase family protein / peptidase
ohkB1	cupin domain-containing protein	WP_079688961.1	321	JmjC domain-containing protein [*Chryseotalea sanaruensis*]	58.67	cupin domain-containing protein
ohkB2	JmjC domain-containing protein	WP_079688960.1	311	JmjC domain-containing protein [*Chryseotalea sanaruensis*]	26.86	JmjC domain-containing protein / cupin-like protein
ohkB3	JmjC domain-containing protein	WP_079688959.1	315	JmjC domain-containing protein [*Chryseotalea sanaruensis*]	45.39	JmjC domain-containing protein / cupin-like protein
ohkA1	hypothetical protein	WP_079688958.1	70	hypothetical protein [*Chryseotalea sanaruensis*]	80.33	precursor peptide
ohkA2	hypothetical protein	WP_079688957.1	69	hypothetical protein [*Chryseotalea sanaruensis*]	80.33	precursor peptide
ohkC	hypothetical protein	WP_079688956.1	124	hypothetical protein [*Chryseotalea sanaruensis*]	56.56	unknown function
ohkD	carboxypeptidase-like regulatory domain-containing protein	WP_159453774.1	497	carboxypeptidase-like regulatory domain-containing protein [*Chryseotalea sanaruensis*]	39.80	carboxypeptidase-like regulatory domain-containing protein
ohkE	hypothetical protein	WP_079688954.1	357	hypothetical protein [*Chryseotalea sanaruensis*]	55.49	lysophospholipid acyltransferase (LPLATs)
ohkR	UPF0489 family protein	WP_159453773.1	307	UPF0489 family protein [*Chryseotalea sanaruensis*]	51.51	peptide arginase

Gene	NCBI annotation [*Ohtaekwangia koreensis*]	Accession	AA	Protein homolog (BLAST)	Identity [%]	Proposed function
ohkF	S41 family peptidase	WP_159453772.1	636	S41 family peptidase [*Chryseotalea sanaruensis*]	40.77	peptidase
ohkG	radical SAM/SPASM domain-containing protein	WP_079688951.1	486	radical SAM/SPASM domain-containing protein [*Chryseotalea sanaruensis*]	70.16	radical SAM/SPASM domain-containing protein
ohkH	hypothetical protein	WP_079688950.1	284	hypothetical protein [*Chryseotalea sanaruensis*]	45.71	unknown function
ohkI	S8 family serine peptidase	WP_079688949.1	371	S8 family serine peptidase [*Chryseotalea sanaruensis*]	43.89	serine peptidase
ohkJ	ABC transporter permease	WP_079688948.1	777	ABC transporter permease [*Chryseotalea sanaruensis*]	51.48	ABC transporter permease
ohkK	hypothetical protein	WP_079688947.1	90	hypothetical protein [*Chitinophaga sancti*]	52.86	unknown function
ohkL	cupin-like domain-containing protein	WP_079688946.1	277	cupin-like domain-containing protein [*Chryseotalea sanaruensis*]	53.88	cupin domain-containing protein
ohkM	ABC transporter ATP-binding protein	WP_079688945.1	586	ABC transporter ATP-binding protein [*Chryseotalea sanaruensis*]	54.08	ABC transporter permease
orf4	tetratricopeptide repeat protein	WP_079688944.1	964	tetratricopeptide repeat protein [*Chryseolinea lacunae*]/ Signal transduction histidine kinase [*Ohtaekwangia koreensis*]	65.37	histidine kinase

Table S4. Identity and similarity between protein products of the postulated genetic locus harboring the *flm* and the *ohk* BGCs as displayed in Figure 1 (main text).

NCBI annotation [*Flagellimonas meridianipacifica*]	Gene [*F. m.*]	Accession	NCBI annotation [*Ohtaekwangia koreensis*]	Gene [*O. k.*]	Accession	Identity [%]	Similarity [%]
radical SAM/SPASM domain-containing protein	*flmE*	WP_106143369.1	radical SAM/SPASM domain-containing protein	*ohkG*	WP_079688951.1	43.99	58.45
UPF0489 family protein	*flmR*	WP_106143370.1	UPF0489 family protein	*ohkR*	WP_159453773.1	40.26	56.23
S41 family peptidase	*flmF*	WP_106143371.1	S41 family peptidase	*ohkF*	WP_159453772.1	21.48	37.88
hypothetical protein	*flmG*	WP_106143372.1	hypothetical protein	*ohkH*	WP_079688950.1	20.00	37.42
S8 family serine peptidase	*flmH*	WP_106143373.1	S8 family serine peptidase	*ohkI*	WP_079688949.1	15.36	25.35
cupin-like domain-containing protein	*flmI*	WP_106143374.1	cupin-like domain-containing protein	*ohkL*	WP_079688946.1	31.06	45.73
ABC transporter ATP-binding protein	*flmL*	WP_158259058.1	ABC transporter ATP-binding protein	*ohkM*	WP_079688945.1	28.06	50.51

Sequence Analysis of Peptide Arginases

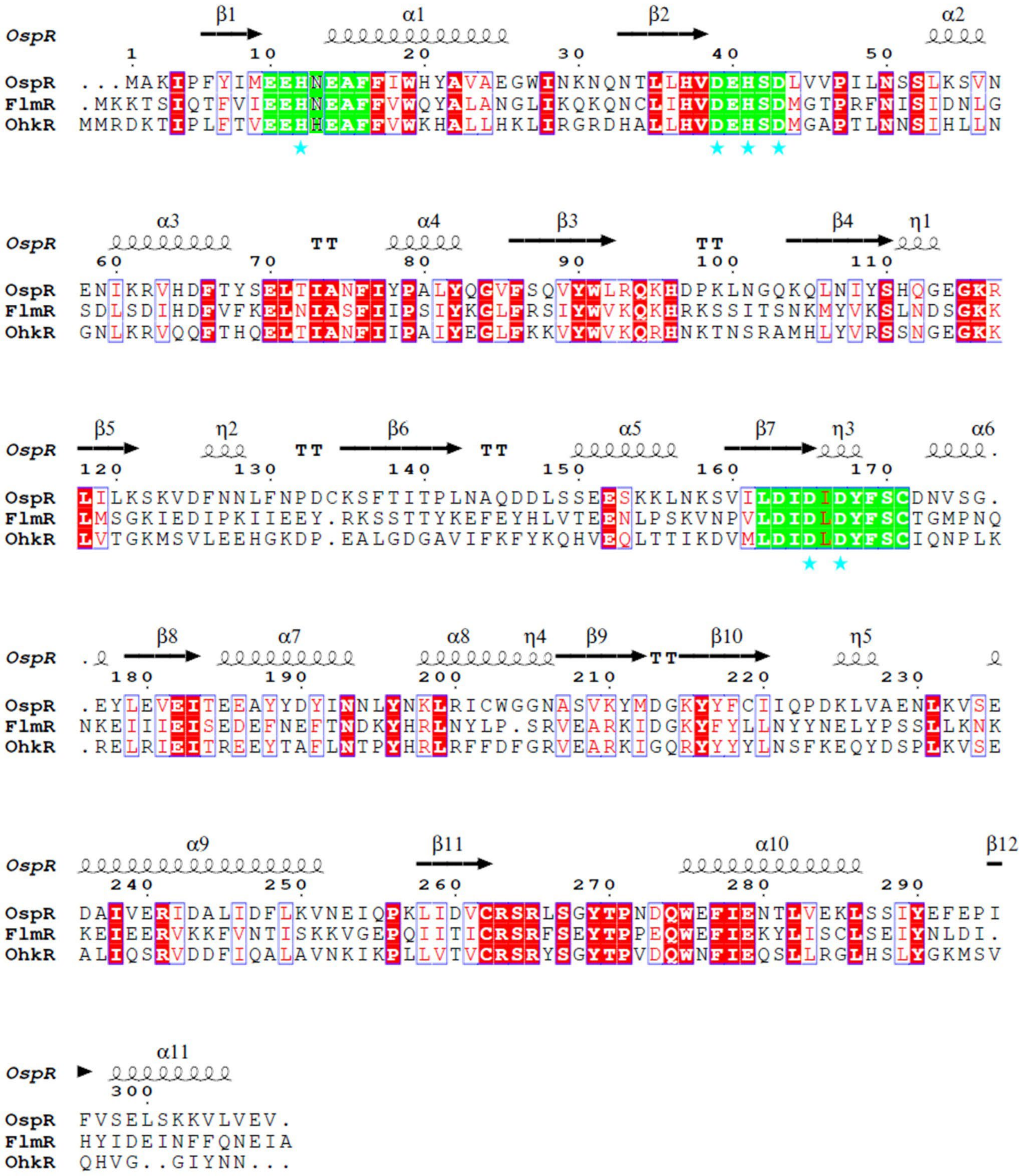

Figure S1. Multiple sequence alignment of OspR, FlmR, and OhkR. Protein sequences were aligned with Clustal Omega[5] using default settings. ESPript 3.0[6] was used for graphic creation. The secondary structure depiction is based on the OspR PDB file (PDB ID 8BRP). Active site residues are marked with cyan asterisks and the three conserved sequence motifs are highlighted in green.

Sequence Analysis of 'DD(I/V)LF'-type Precursors

Table S5. Accession numbers for 'DD(I/V)LF'-type precursors used in the WebLogo sequence in Figure 1 (main text). n.a. = not available.

Accession	Abbreviation	Organism	Strain/Isolate number*	Phylum
WP_161598257		*Maribacter flavus*	KCTC 42508	Bacteroidetes
WP_154920446		*Maribacter flavus*	KCTC 42508	Bacteroidetes
WP_154920448		*Maribacter flavus*	KCTC 42508	Bacteroidetes
WP_127123895.1		*Chryseotalea sanaruensis*	strain Ys	Bacteroidetes
WP_127123894.1		*Chryseotalea sanaruensis*	strain Ys	Bacteroidetes
WP_079688958.1	OhkA1	*Ohtaekwangia koreensis*	DSM 25262	Bacteroidetes
WP_079688957.1	OhkA2	*Ohtaekwangia koreensis*	DSM 25262	Bacteroidetes
WP_160292160.1		*Pedobacter lusitanus*	NL 19	Bacteroidetes
WP_041886396.1		*Pedobacter lusitanus*	NL 19	Bacteroidetes
WP_157752932.1		*Chitinophaga* sp.	MD30	Bacteroidetes
WP_177318517.1		*Chitinophaga sancti*	DSM 784	Bacteroidetes
WP_158259055.1	FlmA1	*Muricauda pacifica* (now *Flagellimonas meridianipacifica*)	DSM 25027	Bacteroidetes
WP_158259056.1	FlmA2	*Muricauda pacifica* (now *Flagellimonas meridianipacifica*)	DSM 25027	Bacteroidetes
WP_158259057.1	FlmA3	*Muricauda pacifica* (now *Flagellimonas meridianipacifica*)	DSM 25027	Bacteroidetes
WP_162618564.1		*Pedobacter yulinensis*	YL28-9	Bacteroidetes
WP_162618565.1		*Pedobacter yulinensis*	YL28-9	Bacteroidetes
WP_157287991.1		*Pedobacter cryoconitis*	PAMC 27485	Bacteroidetes
HET6256336.1		*Puia* sp.	SMAG_U5183*	Bacteroidetes
WP_264732610.1		*Chitinophaga nivalis*	PC14	Bacteroidetes
WP_211977249.1		*Chitinophaga hostae*	2R12	Bacteroidetes
WP_236857428.1		*Chryseobacterium* sp.	MEBOG06	Bacteroidetes
WP_313002668.1		*Chryseobacterium gleum*	CTOTU48830*	Bacteroidetes
WP_268225309.1		*Sinomicrobium oceani*	PAP.21	Bacteroidetes
WP_268225310.1		*Sinomicrobium oceani*	PAP.21	Bacteroidetes
WP_268225311.1		*Sinomicrobium oceani*	PAP.21	Bacteroidetes
MBX2968298.1		Cyclobacteriaceae bacterium	ACE_BAC25*	Bacteroidetes
WP_220251327.1		*Chitinophaga rhizophila*	B61	Bacteroidetes
WP_157752932.1		*Chitinophaga pendula*	MD30B	Bacteroidetes
WP_184623764.1		*Pedobacter cryoconitis*	M2T3	Bacteroidetes
WP_184623766.1		*Pedobacter cryoconitis*	M2T3	Bacteroidetes
WP_325527324.1		*Chitinophaga* sp.	SMAG_U13719*	Bacteroidetes
WP_325527326.1		*Chitinophaga* sp.	SMAG_U13719*	Bacteroidetes
WP_325527328.1		*Chitinophaga* sp.	SMAG_U13719*	Bacteroidetes
WP_325527330.1		*Chitinophaga* sp.	SMAG_U13719*	Bacteroidetes
WP_325527332.1		*Chitinophaga* sp.	SMAG_U13719*	Bacteroidetes
WP_325527334.1		*Chitinophaga* sp.	SMAG_U13719*	Bacteroidetes
WP_372976348.1		*Mucilaginibacter* sp.	SO_2017_GW1_139*	Bacteroidetes
WP_298733939.1		*Chitinophaga* sp. (uncultured)		Bacteroidetes
WP_298733940.1		*Chitinophaga* sp. (uncultured)		Bacteroidetes
WP_298733942.1		*Chitinophaga* sp. (uncultured)		Bacteroidetes
WP_265819070.1		*Pedobacter* sp.	GR22-10	Bacteroidetes
WP_274210576.1		*Mucilaginibacter* sp.	KACC 22773	Bacteroidetes

Accession	Abbreviation	Organism	Strain/Isolate number*	Phylum
WP_274210583.1		*Mucilaginibacter* sp.	KACC 22773	Bacteroidetes
WP_188834828.1		*Mucilaginibacter* sp.	OAE612	Bacteroidetes
WP_188833791.1		*Mucilaginibacter* sp.	OAE612	Bacteroidetes
WP_188834828.1		*Mucilaginibacter rubeus*	2025	Bacteroidetes
WP_188833791.1		*Mucilaginibacter rubeus*	2025	Bacteroidetes
MBS1663072.1		Bacteroidota bacterium	SZAS-47*	Bacteroidetes
MBS1663074.1		Bacteroidota bacterium	SZAS-47*	Bacteroidetes
WP_212004685.1		*Chitinophaga* sp.	HK235	Bacteroidetes
WP_212004686.1		*Chitinophaga* sp.	HK235	Bacteroidetes
MDW3191850.1		Cytophagales bacterium	Padam50*	Bacteroidetes
MDW3191851.1		Cytophagales bacterium	Padam50*	Bacteroidetes
MDW3191852.1		Cytophagales bacterium	Padam50*	Bacteroidetes
MDW3191853.1		Cytophagales bacterium	Padam50*	Bacteroidetes
NOU18122.1		Bacteroidales bacterium	P-RSF-IL-12*	Bacteroidetes
n.a.		Bacteroidales bacterium	P-RSF-IL-12*	Bacteroidetes

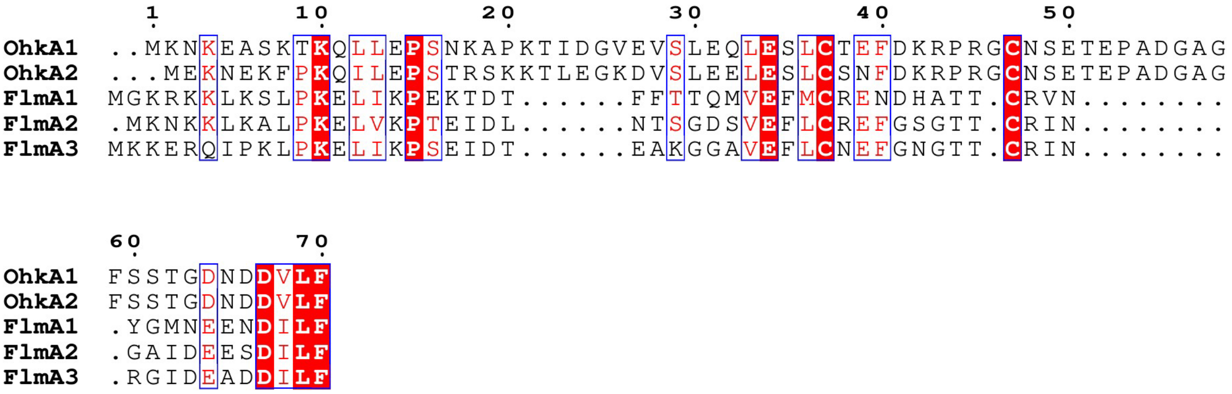

Figure S2. Multiple sequence alignment of OhkA1-2 and FlmA1-3 precursors. Protein sequences were aligned with Clustal Omega using default settings.[5] ESPript 3.0 was used for graphic creation.[6]

Table S6. Sequence identities and sequence similarities (in parentheses) in percentage of Flm and Ohk precursors calculated with EMBOSS Needle default settings.[5]

	FlmA1	FlmA2	FlmA3	OhkA1	OhkA2
**FlmA1**	100				
**FlmA2**	53.6 (69.6)	100			
**FlmA3**	50.0 (67.9)	60.7 (73.2)	100		
**OhkA1**	22.1 (35.1)	26.3 (44.7)	26.0 (37.7)	100	
**OhkA2**	19.0 (34.2)	22.1 (39.0)	23.4 (37.7)	71.4 (82.9)	100

Protein Expression Yield

Table S7. Average yields of His$_6$-tagged precursor proteins (FlmA1-A3, OhkA1-A2), His$_6$-tagged FlmR and His$_6$-MBP-tagged OhkR peptide arginases produced in *E. coli*. Protein concentrations were determined as described above in the “Experimental Procedures” section (subsection (Co-)Expression, Precursor and Arginase Purification, and Peptide Digests). Molecular weights and E 1% were calculated based on the tagged amino acid sequence with Protparam.[7] E 1 % is the theoretical mass extinction coefficient for a 10 mg/L solution of the measured protein.

Protein	Yield for 1 L culture	Molecular weight [kDa]	E 1%
FlmA1	3.7 ± 0.7 mg	9.07	3.29
FlmA2	1.5 ± 0.1 mg	8.51	1.75
FlmA3	5.1 ± 2.4 mg	8.69	1.72
OhkA1	13.1 ± 5.1 mg	10.01	1.61
OhkA2	3.4 ± 2.4mg	10.06	1.61
FlmR	1.4 ± 0.4 mg	38.91	11.90
MBP-OhkR	0.5 ± 0.2 mg	80.57	13.44

SDS-PAGE Analysis of (Modified) Precursor Peptides and Peptide Arginases

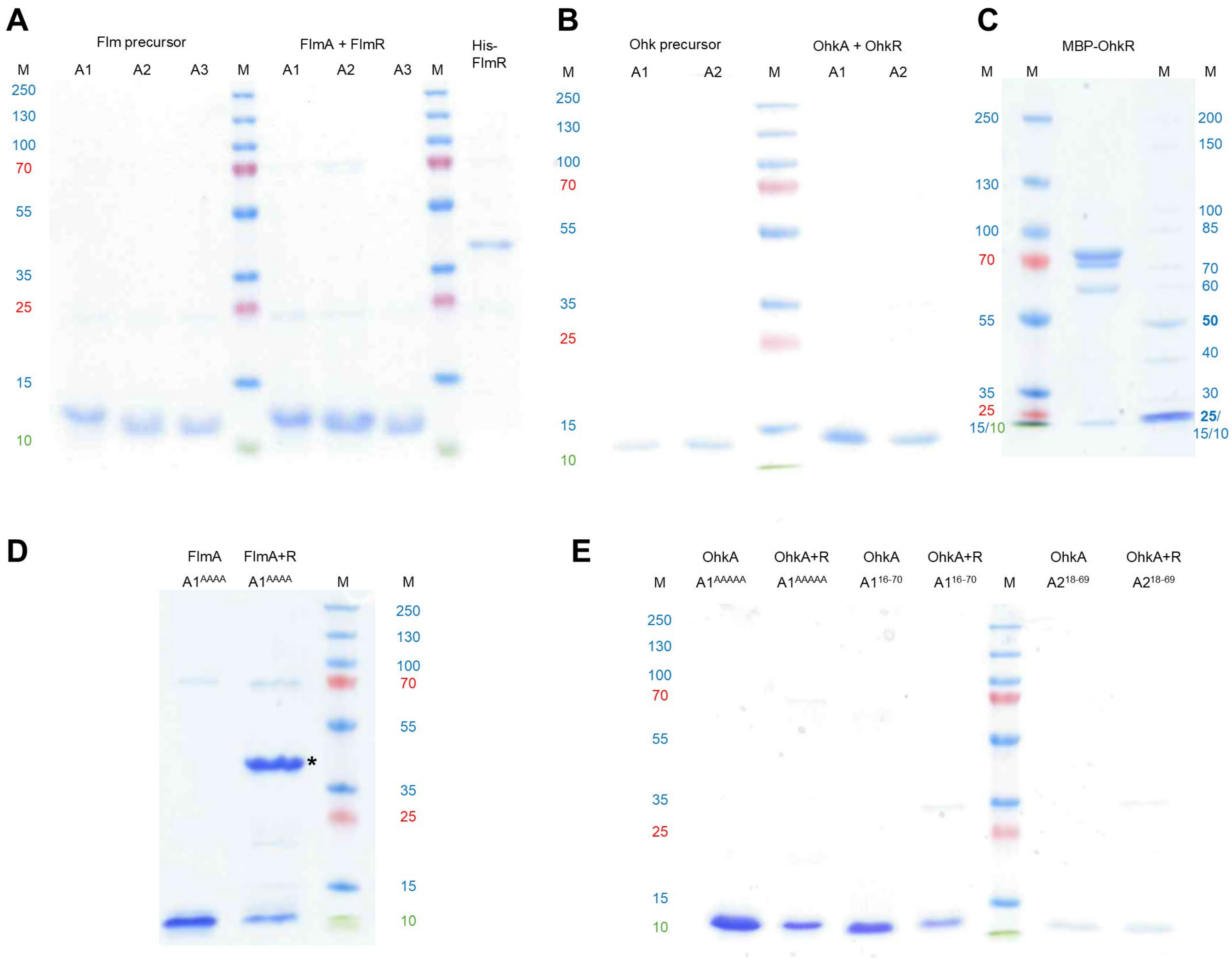

Figure S3. SDS-PAGE analysis of (**A**) Flm precursor peptides and peptide arginase, (**B**) Ohk precursor peptides, (**C**) MBP-tagged Ohk peptide arginase, and (**D**) and (**E**) precursor variants. Co-eluted FlmR in (**D**) is marked with an asterisk. 5 µg of protein was loaded onto 15% SDS-PAGE gels; however, MBP-OhkR in panel C was separated on a 10% SDS-PAGE gel. Abbreviations: FlmA1^{AAAA} = FlmA1-D53A-I54A-L55A-F56A, OhkA1^{AAAAA} = OhkA1-D66A-D67A-V68A-L69A-F70A.

Mass Spectrometry Data

Co-expression of peptide arginase with native precursor substrates

Table S8. Calculated and observed masses of modified and unmodified precursor peptides fragments. The unmodified peptide fragment was utilised as reference for calculating mass differences between species. Abbreviations: FA = formic acid, Orn = ornithine, R_t = retention time, n.d. = not detected, n.a. = not applicable.

Precursor	Peptide fragment	Digest	Species	R_t / min	Charge state	Calculated mass	Observed mass	Error Δppm
FlmA1	FlmA1$^{22\text{-}37}$ TFFTTQMVEFMC**R**END	FA	unmodified	4.39	+3	666.9513	666.9508	0.795
		FA	1-Orn	4.30	+3	652.9440	652.9435	0.858
	FlmA1$^{38\text{-}53}$ HATTC**R**VNYGMNEEND	FA	unmodified	1.39	+3	618.5895	618.5888	1.229
		FA	1-Orn	1.24	+3	604.5822	604.5818	0.810
FlmA2	FlmA2$^{27\text{-}48}$ SVEFLC**R**EFGSGTTC**R**INGAID	FA	unmodified	3.32	+2	1188.0595	1188.0511	7.121
		FA	1-Orn	n.d.	+2	1167.0486	n.d.	n.a.
		FA	2-Orn	3.24	+2	1146.0377	1146.0332	3.979
	FlmA2$^{27\text{-}48}$ SVEFLC**R**EFGSGTTC**R**INGAID	FA	unmodified	3.32	+3	792.3754	792.3711	5.515
		FA	1-Orn	n.d.	+3	778.3682	n.d.	n.a.
		FA	2-Orn	3.24	+3	764.3609	764.3565	5.796
	FlmA2$^{27\text{-}52}$ (incomplete cleavage) SVEFLC**R**EFGSGTTC**R**INGAIDEESD	FA	unmodified	3.32	+3	945.7568	945.7524	4.705
		FA	1-Orn	n.d.	+3	931.7495	n.d.	n.a.
		FA	2-Orn	3.24	+3	917.7423	917.7379	4.816
FlmA3	FlmA3$^{22\text{-}49}$ TEAKGGAVEFLCNEFGNGTTC**R**IN**R**GID	FA	unmodified	3.48	+4	743.8552	743.8546	0.793
		FA	1-Orn	3.38	+4	733.3502	733.3508	0.871
		FA	2-Orn	3.34	+4	722.8447	722.8448	0.123
OhkA1	OhkA1$^{22\text{-}70}$ TIDGVEVSLEQLESLCTEFDK**R**P**R**GCNSETEPADGAGFSSTGDNDDVLF	LysC	unmodified	4.50	+4	1313.3464	1313.3382	6.245
		LysC	1-Orn	n.d.	+4	1302.8410	n.d.	n.a.
		LysC	2-Orn	4.54	+4	1292.3355	1292.3338	1.315
	OhkA1$^{42\text{-}55}$ K**R**P**R**GCNSETEPAD	FA	unmodified	0.41	+3	520.5792	520.5781	2.113
		FA	1-Orn	0.30	+3	506.5719	506.5716	0.592
		FA	2-Orn	0.30	+3	492.5647	492.5643	0.812
OhkA2	OhkA2$^{26\text{-}69}$ DVSLEELESLCSNFDK**R**P**R**GCNSETEPADGAGFSSTGDNDDVLF	LysC	unmodified	4.05	+4	1181.5225	1181.5205	1.693
		LysC	1-Orn	4.03	+4	1171.0171	1171.0135	3.074
		LysC	2-Orn	4.03	+4	1160.5116	1160.5100	1.379
	OhkA2$^{41\text{-}54}$ K**R**P**R**GCNSETEPAD	FA	unmodified	0.31	+3	520.5792	520.5791	0.192
		FA	1-Orn	0.29	+3	506.5719	506.5718	0.197
		FA	2-Orn	0.29	+3	492.5647	492.5645	0.406

Co-expression of peptide arginase with precursor substrates from the other BGC (precursor swap)

Table S9. Calculated and observed masses of modified and unmodified precursor peptides fragments from precursor swap experiments. The unmodified peptide fragment was utilised as reference for calculating mass differences between species, shown in black. Co-expression data is coloured blue for co-expression with OhkR and green for co-expression with FlmR. Abbreviations: Orn = ornithine, R_t = retention time, n.d. = not determined, n.a. = not applicable.

Precursor	Peptide fragment	Digest	Species	R_t / min	Charge state	Calculated mass	Observed mass	Error Δppm
FlmA1	TDTFFTTQMVEFMC**R**ENDHATTC**R**VNYGMNEENDILF	LysC	unmodified	4.59	+4	1106.47718	1106.4755	1.518
	TDTFFTTQMVEFMC**R**ENDHATTC**R**VNYGMNEENDILF	LysC	unmodified	4.56	+4	1106.47718	1106.4761	0.976
		LysC	1-Orn	4.56	+4	1095.96718	1095.9709	3.394
		LysC	2-Orn	4.56	+4	1085.45719	1085.4655	7.656
FlmA2	ELVKPTEIDLNTSGDSVEFLC**R**EFGSGTTC**R**INGAIDEESDILF	LysC	unmodified	4.73	+4	1205.82839	1205.8259	2.065
		LysC	unmodified	4.73	+5	964.86417	964.8621	2.145
	ELVKPTEIDLNTSGDSVEFLC**R**EFGSGTTC**R**INGAIDEESDILF	LysC	unmodified	4.74	+4	1205.8284	1205.8255	2.405
		LysC	1-Orn	4.74	+4	1195.31238	1195.3206	6.877
		LysC	2-Orn	4.74	+4	1184.8055	1184.8158	8.693
		LysC	unmodified	4.74	+5	964.86417	964.8617	2.560
		LysC	1-Orn	4.74	+5	956.456169	956.4578	1.705
		LysC	2-Orn	4.74	+5	948.048173	948.0530	5.091
FlmA3	GGAVEFLCNEFGNGTTC**R**IN**R**GIDEADDILF	LysC	unmodified	4.50	+3	1116.18709	1116.1811	5.366
		LysC	unmodified	4.50	+4	837.39213	837.3885	4.335
	GGAVEFLCNEFGNGTTC**R**IN**R**GIDEADDILF	LysC	unmodified	4.60	+3	1116.18709	1116.1816	4.919
		LysC	1-Orn	4.60	+3	1102.1633	1102.1733	5.916
		LysC	2-Orn	4.60	+3	1088.14997	1088.1653	6.663
		LysC	unmodified	4.60	+4	837.39213	837.3889	3.857
		LysC	1-Orn	4.60	+4	826.882137	826.8818	5.902
		LysC	2-Orn	4.60	+4	816.3689	816.3748	7.227
OhkA1	K**R**P**R**GCNSETEPAD	FA	unmodified	0.30	+2	780.36518	780.3631	2.665
		FA	unmodified	0.30	+3	520.57921	520.5781	2.132
	K**R**P**R**GCNSETEPAD	FA	unmodified	0.30	+2	780.36518	780.3648	0.487
		FA	1-Orn	0.30	+2	759.35428	759.3546	0.421
		FA	2-Orn	0.30	+2	738.34338	n. a.	n. a.
		FA	unmodified	0.30	+3	520.57921	520.5792	0.019
		FA	1-Orn	0.30	+3	506.57194	506.5722	0.513
		FA	2-Orn	0.30	+3	492.56468	n. a.	n. a.
OhkA2	K**R**P**R**GCNSETEPAD	FA	unmodified	0.30	+2	780.36518	780.3633	2.409
		FA	unmodified	0.30	+3	520.57921	520.5785	1.364
	K**R**P**R**GCNSETEPAD	FA	unmodified	0.30	+2	780.36518	780.3646	0.743
		FA	1-Orn	0.30	+2	759.35428	759.3542	0.105
		FA	2-Orn	0.30	+2	738.34338	n. a.	n. a.
		FA	unmodified	0.30	+3	520.57921	520.5790	0.403
		FA	1-Orn	0.30	+3	506.57194	506.5722	0.513
		FA	2-Orn	0.30	+3	492.56468	492.5663	3.289

Co-expression of precursor variants: Investigation of the 'DD(I/V)LF' motif

Table S10. Calculated and observed masses of modified and unmodified precursor variant peptide fragments. The unmodified peptide variant fragment was utilised as reference for calculating mass differences between species. Abbreviations: FA = formic acid, Orn = ornithine, R_t = retention time, n.d. = not detected, n.a. = not applicable.

Precursor	Peptide fragment	Digest	Species	R_t / min	Charge state	Calculated mass	Observed mass	Error Δppm
FlmA1-D53A-I54A-L55A-F56A	FlmA1 TFFTTQMVEFMC**R**END	FA	unmodified	4.52	+2	999.9234	999.9251	1.700
		FA	1-Orn	4.45	+2	978.9125	978.9171	4.699
	FlmA1 HATTC**R**VNYGMNEENAAAA	FA	unmodified	1.61	+2	1011.9415	1011.9435	1.976
		FA	1-Orn	1.60	+2	990.9306	990.9315	0.908
OhkA1-D66A-D67A-V68A-L69A-F70A	OhkA1 K**R**P**R**GCNSETEPAD	FA	unmodified	0.32	+3	520.5792	520.5782	1.921
		FA	1-Orn	0.33	+3	506.5719	506.5713	1.184
		FA	2-Orn	0.33	+3	492.5647	492.5642	1.015

Co-expression of precursor variants: Deletion of OhkA1 and OhkA2 N-termini

Table S11. Calculated and observed masses of modified and unmodified precursor variant peptide fragments. The unmodified peptide variant fragment was utilised as reference for calculating mass differences between species. Abbreviations: FA = formic acid, Orn = ornithine, R_t = retention time, n.d. = not detected, n.a. = not applicable.

Precursor	Peptide fragment	Digest	Species	R_t / min	Charge state	Calculated mass	Observed mass	Error Δppm
$OhkA1^{16\text{-}70}$	OhkA1 K**R**P**R**GCNSETEPAD	FA	unmodified	0.32	+3	520.5792	520.5786	1.153
		FA	1-Orn	0.33	+3	506.5719	506.5718	0.197
		FA	2-Orn	0.33	+3	492.5647	492.5641	1.218
$OhkA2^{18\text{-}69}$	OhkA2 K**R**P**R**GCNSETEPAD	FA	unmodified	0.35	+3	520.5792	520.5792	0.000
		FA	1-Orn	0.34	+3	506.5719	n.d.	n.a.
		FA	2-Orn	0.34	+3	492.5647	492.5641	1.218

Activity of FlmR on its Native Substrates FlmA1, FlmA2, and FlmA3 (*in vivo*)

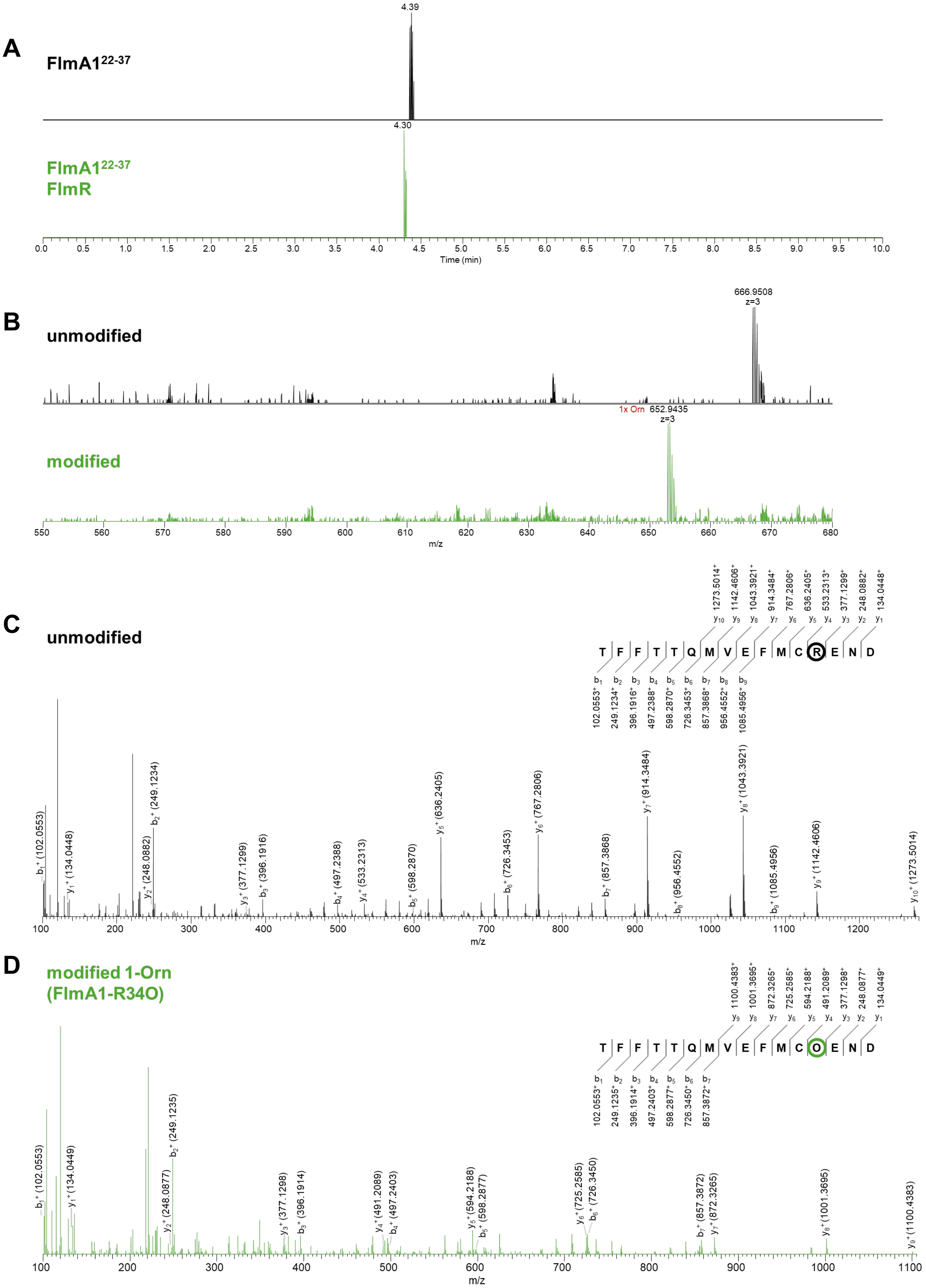

Figure S4. HPLC-MS/MS^2 analysis of FlmR arginase with its native substrate FlmA1: Formic acid digested precursor of *flmA1* and *flmR* (co-)expression. (**A**) EICs for the $[M+3H]^{3+}$ *m/z* 666.9508 for the unmodified and 652.9435 for the 1-Orn species. (**B**) MS^1 of the unmodified (substrate) and modified (product) peaks. (**C**) MS^2 spectrum of the unmodified product (*m/z* 666.9508, $[M+3H]^{3+}$; peptide sequence and calculated monoisotopic mass of detected fragment ions are shown on the right). (**D**) MS^2 spectrum of the modified product (1-Orn, *m/z* 652.9435, $[M+3H]^{3+}$; peptide sequence and calculated monoisotopic mass of detected fragment ions are shown on the right). O / Orn = ornithine.

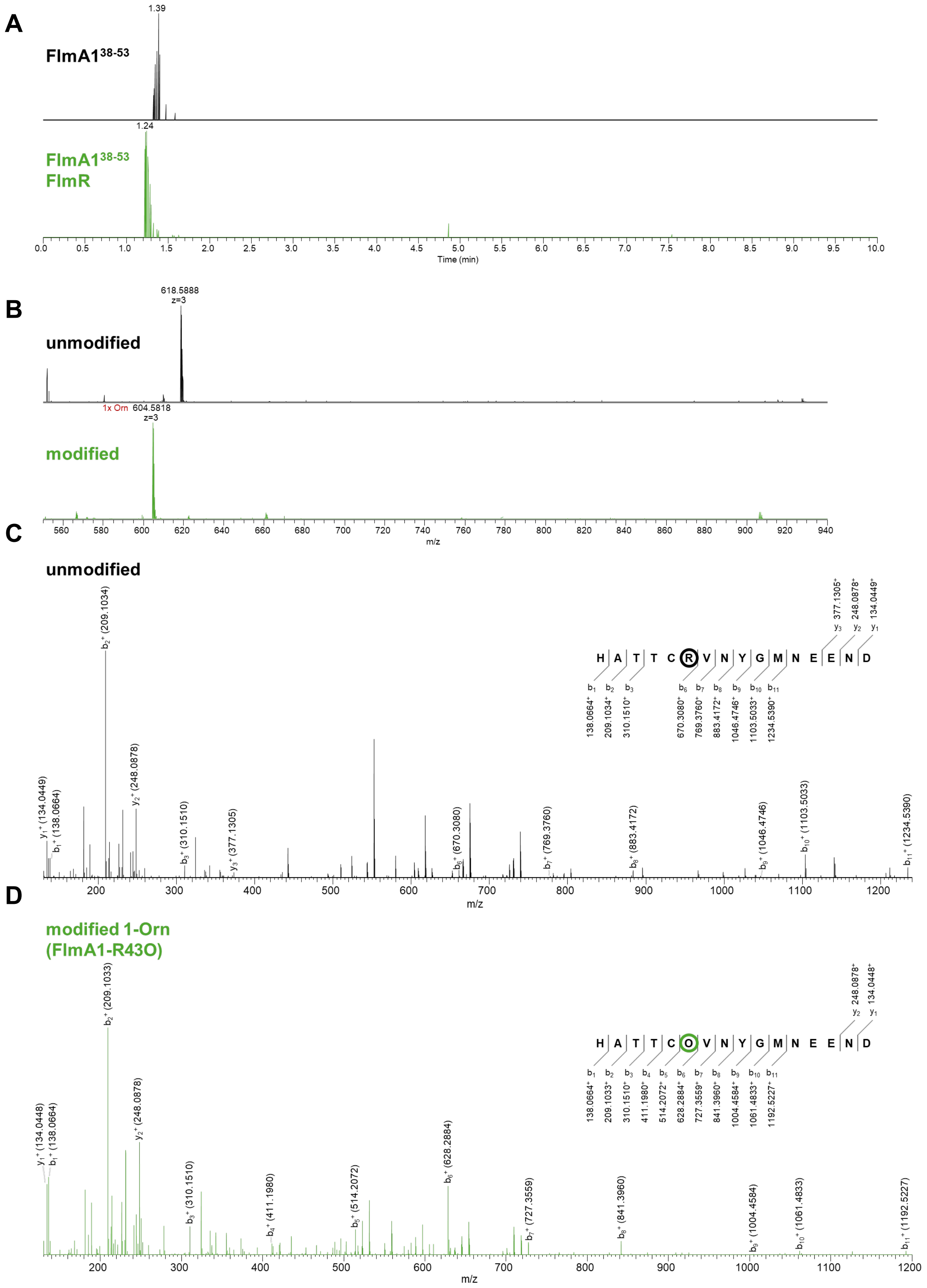

Figure S5. HPLC-MS/MS^2 analysis of FlmR arginase with its native substrate FlmA1: Formic acid digested precursor of *flmA1* and *flmR* (co-)expression. (**A**) EICs for the $[M+3H]^{3+}$ *m/z* 618.5888 for the unmodified and 604.5818 for the 1-Orn species. (**B**) MS^1 of the unmodified (substrate) and modified (product) peaks. (**C**) MS^2 spectrum of the unmodified product (*m/z* 618.5888, $[M+3H]^{3+}$; peptide sequence and calculated monoisotopic mass of detected fragment ions are shown on the right). (**D**) MS^2 spectrum of the modified product (1-Orn, *m/z* 604.5818, $[M+3H]^{3+}$; peptide sequence and calculated monoisotopic mass of detected fragment ions are shown on the right). O / Orn = ornithine.

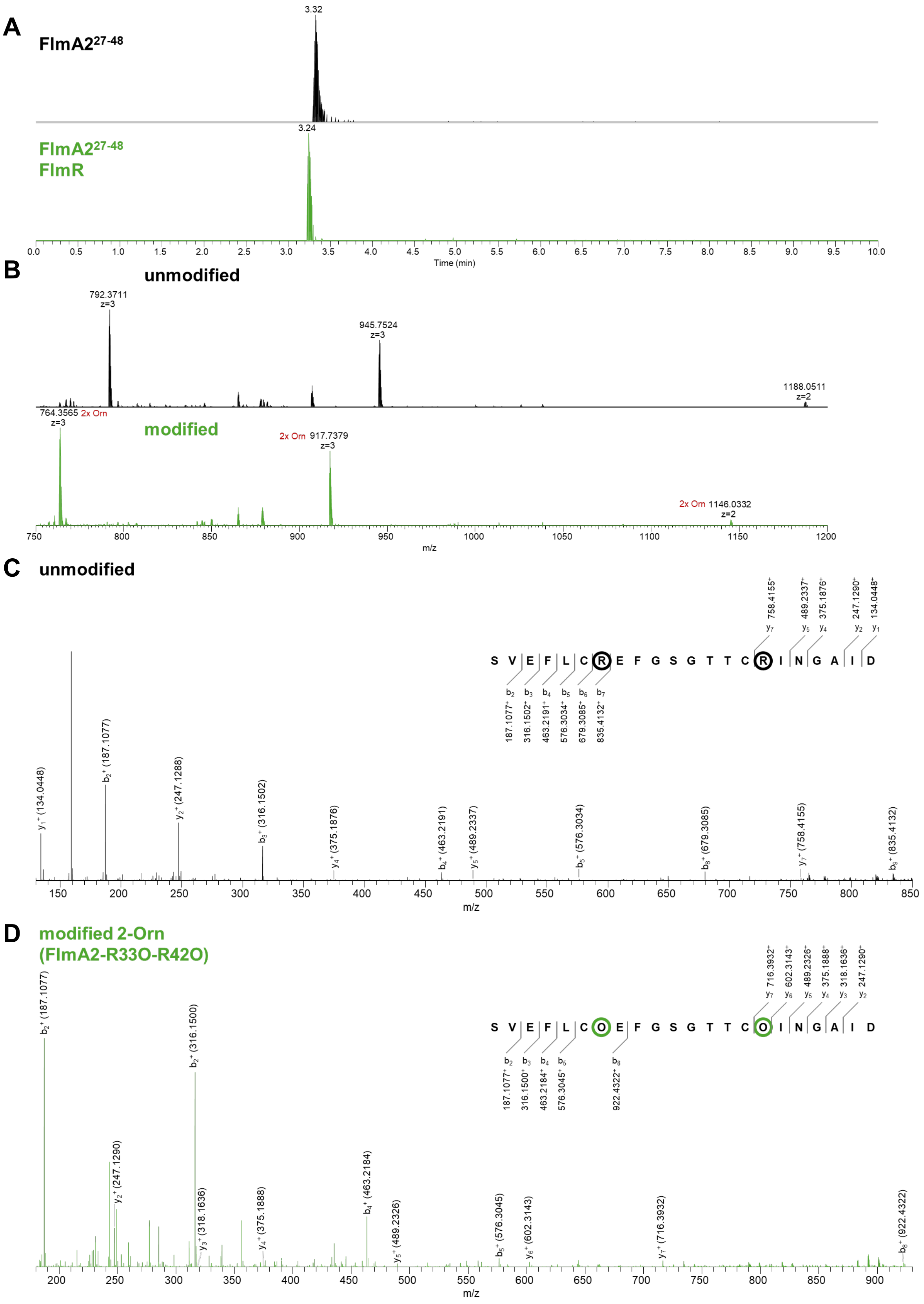

Figure S6. HPLC-MS/MS[2] analysis of FlmR arginase with its native substrate FlmA2: Formic acid digested precursor of *flmA2* and *flmR* (co-)expression. (**A**) EICs for the $[M+2H]^{2+}$ *m/z* 1188.0511, $[M+3H]^{3+}$ *m/z* 792.3711 for the unmodified and 1146.0332, 764.3565 for the 2-Orn species, respectively. $[M+3H]^{3+}$ *m/z* 945.7524 for the unmodified and 917.7379 for the 2-Orn species originate from incomplete digestion (fragment FlmA2$^{27-52}$). (**B**) MS[1] of the unmodified (substrate) and modified (product) peaks. (**C**) MS[2] spectrum of the unmodified product (*m/z* 1188.0511, $[M+2H]^{2+}$; peptide sequence and calculated monoisotopic mass of detected fragment ions are shown on the right). (**D**) MS[2] spectrum of the modified product (2-Orn, *m/z* 1146.0332, $[M+3H]^{3+}$; peptide sequence and calculated monoisotopic mass of detected fragment ions are shown on the right). O / Orn = ornithine.

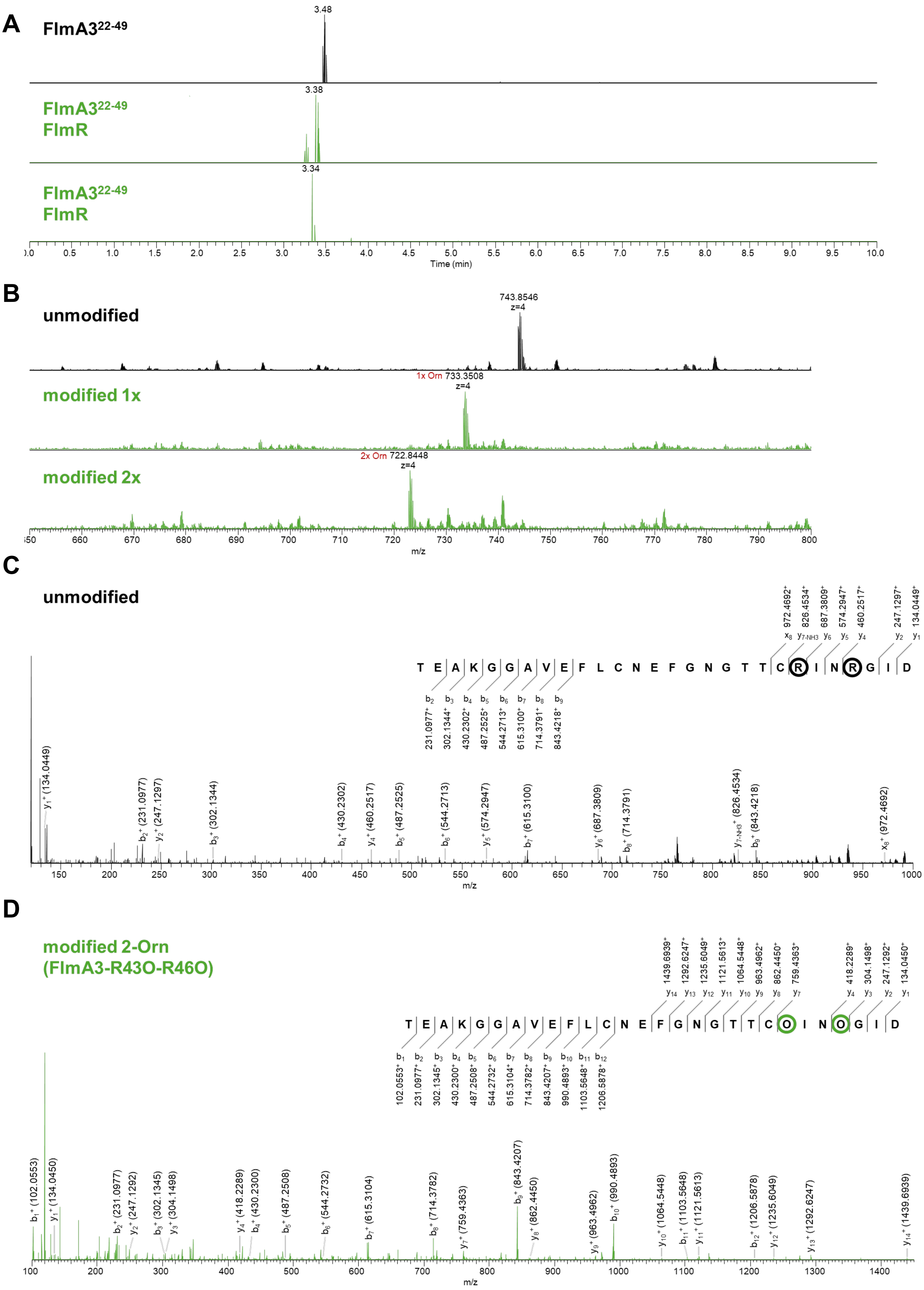

Figure S7. HPLC-MS/MS[2] analysis of FlmR arginase with its native substrate FlmA3: Formic acid digested precursor of *flmA3* and *flmR* (co-)expression. (**A**) EICs for the $[M+4H]^{4+}$ *m/z* 743.8546 for the unmodified, 733.3508 and 722.8448 for the 1-Orn and 2-Orn species, respectively. (**B**) MS[1] of the unmodified (substrate) and 1x and 2x modified (product) peaks. No unmodified peak could be detected in the co-expression sample. (**C**) MS[2] spectrum of the unmodified product (*m/z* 743.8546, $[M+4H]^{4+}$; peptide sequence and calculated monoisotopic mass of detected fragment ions are shown on the right). (**D**) MS[2] spectrum of the modified product (2-Orn, *m/z* 722.8448, $[M+4H]^{4+}$; peptide sequence and calculated monoisotopic mass of detected fragment ions are shown on the right). O / Orn = ornithine.

Directionality of arginine-to-ornithine conversion for FlmR

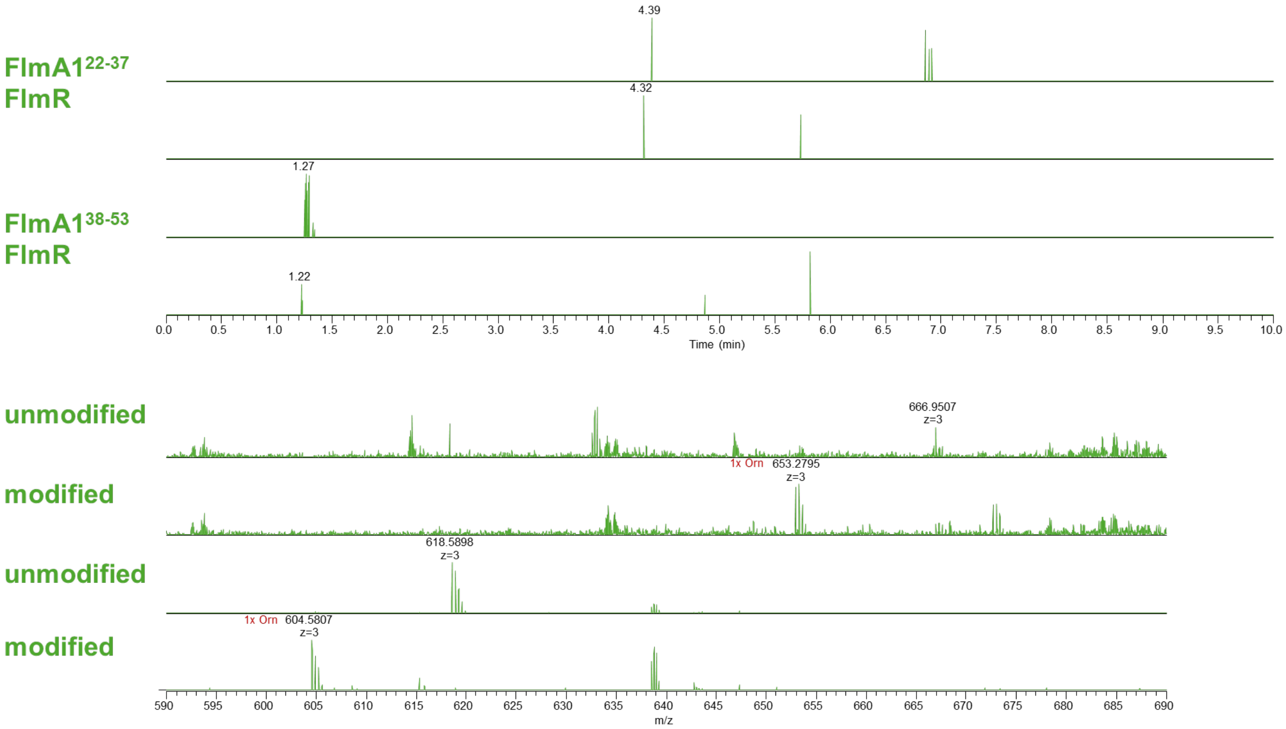

Figure S8. HPLC-MS analysis of FlmR arginase with its native substrate FlmA1. Formic acid digested precursor of *flmA1* and *flmR* (co-)expression. EICs for the $[M+3H]^{3+}$ *m/z* 666.9507 and 618.5898 for the unmodified, 653.2795 and 604.5807 for the 1-Orn species. MS^1 of the unmodified (substrate) and modified (product) peaks. For each fragment unmodified and modified peaks could be detected in the co-expression sample, thus no sequence of modification is determinable. O / Orn = ornithine.

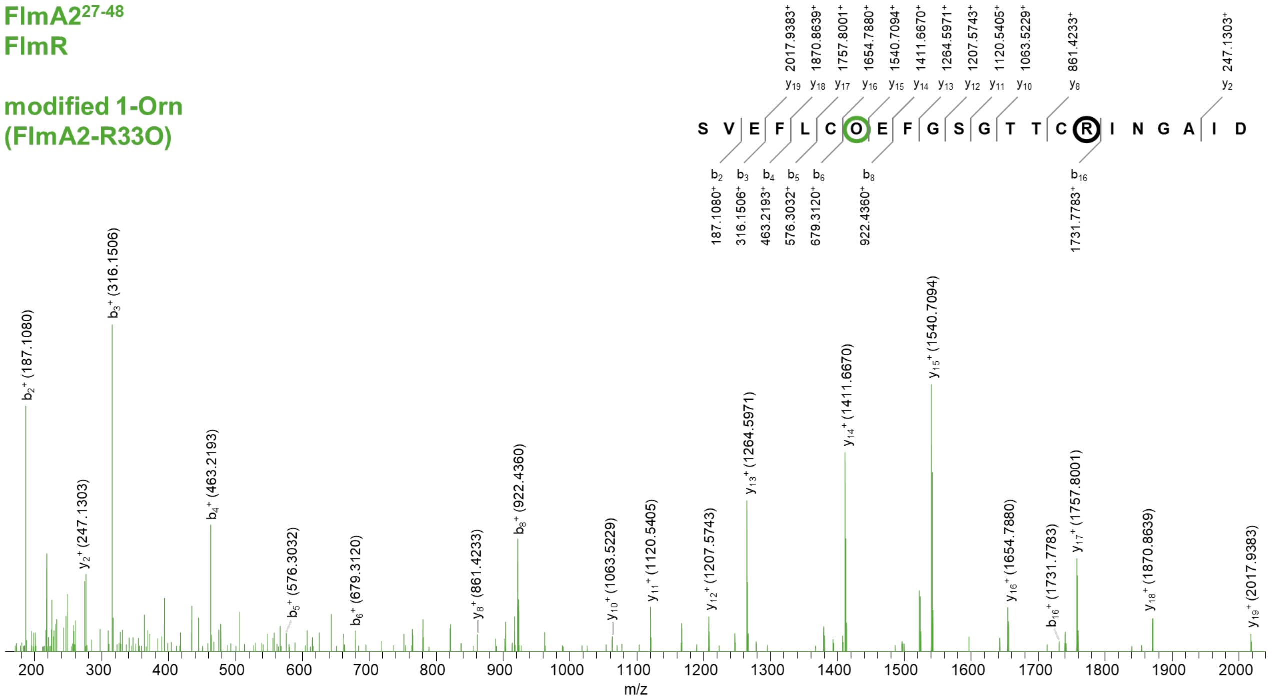

Figure S9. HPLC-MS^2 analysis of FlmR arginase with its native substrate FlmA2: Formic acid digested precursor of *flmA2* and *flmR* co-expression. MS^2 spectrum of the modified product (1-Orn, *m/z* 1167,0486, $[M+2H]^{2+}$; peptide sequence and calculated monoisotopic mass of detected fragment ions are shown on the right). O / Orn = ornithine.

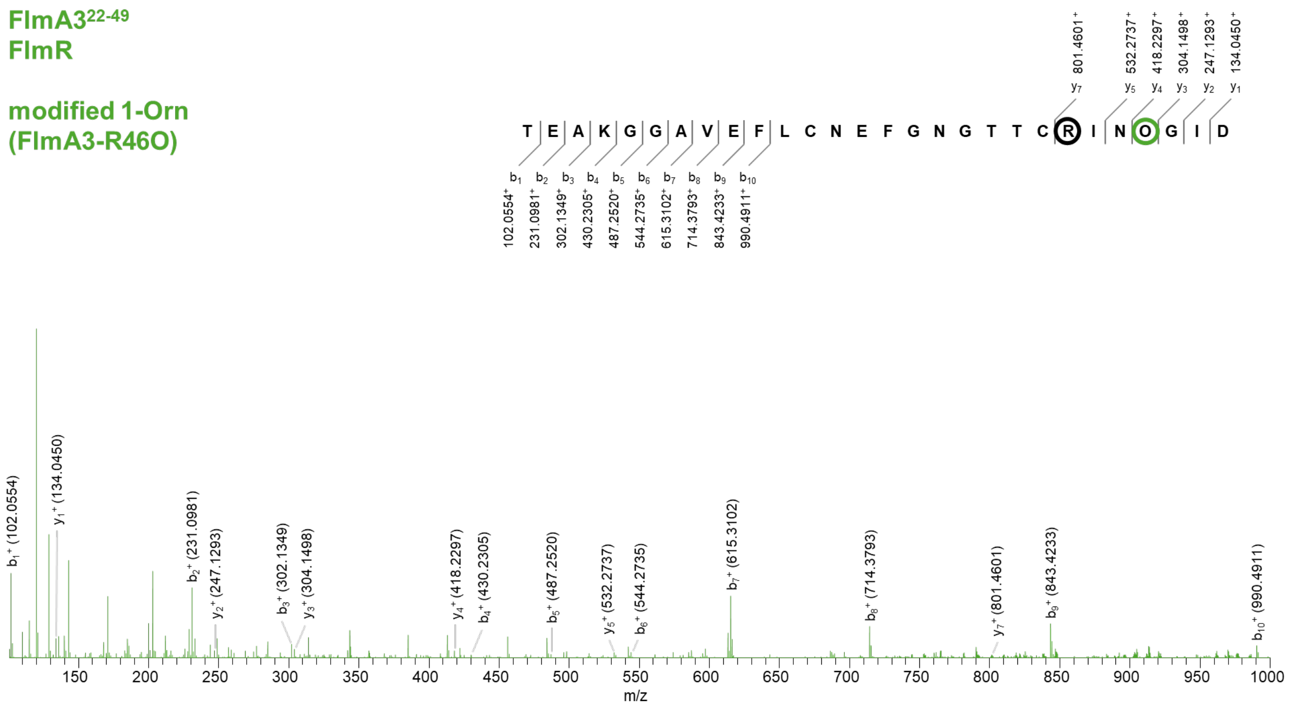

Figure S10. HPLC-MS^2 analysis of FlmR arginase with its native substrate FlmA3: Formic acid digested precursor of *flmA3* and *flmR* co-expression. MS^2 spectrum of the modified product (1-Orn, *m/z* 733.3497, $[M+4H]^{4+}$; peptide sequence and calculated monoisotopic mass of detected fragment ions are shown on the right). O / Orn = ornithine.

Activity of OhkR on its Native Substrates OhkA1 and OhkA2 (*in vivo*)

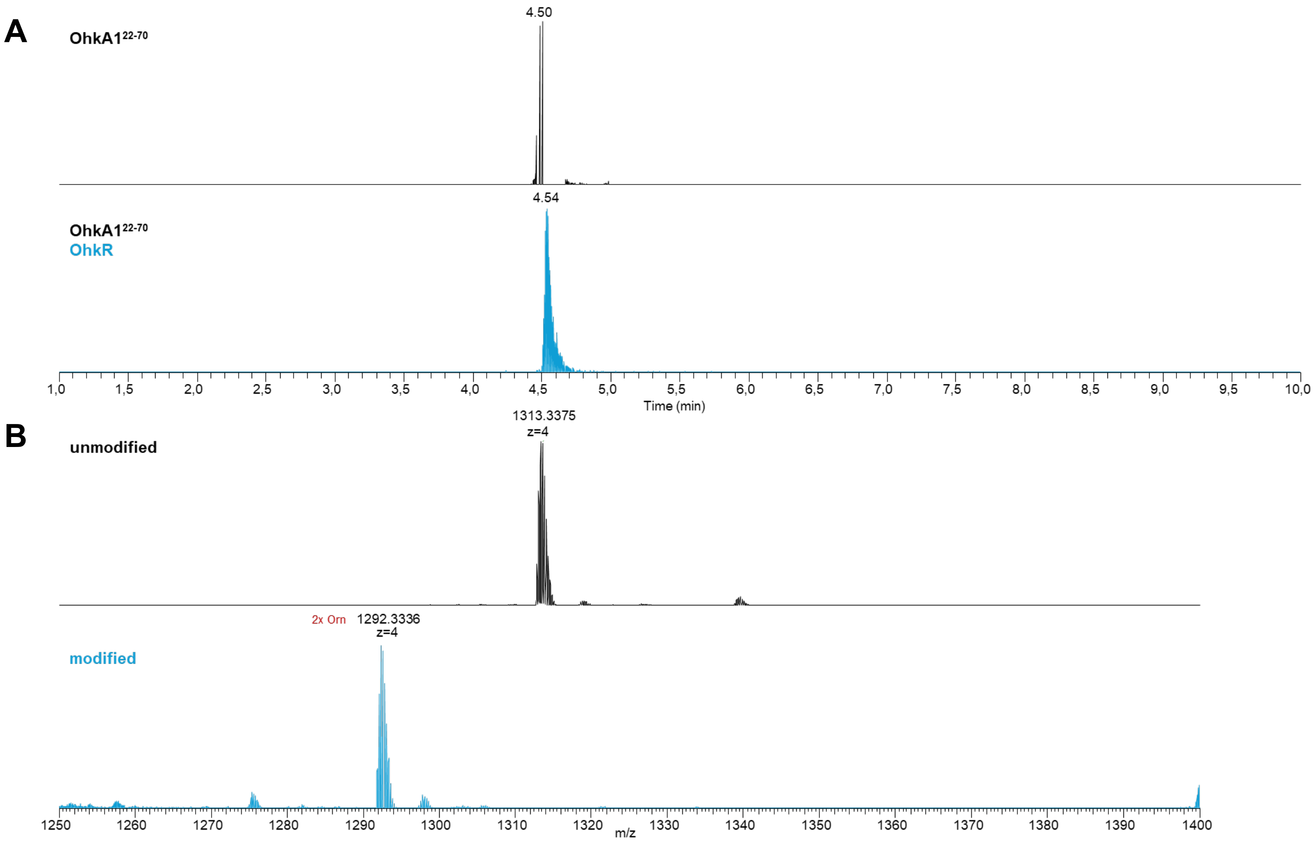

Figure S11. HPLC-MS analysis of OhkR arginase (blue) with native substrate OhkA1: LysC digested precursor of *ohkA1* and *ohkR* (co-)expression. (**A**) EICs for the $[M+4H]^{4+}$ *m/z* 1313.3464 for the unmodified and $[M+4H]^{4+}$ *m/z* 1292.3355 for the 2-Orn species, respectively. (**B**) MS^1 of the unmodified (substrate) and modified (product) peaks. Orn = ornithine. MS^2 analysis was not possible because the resulting fragment ($OhkA1^{22-70}$) was too large for MS^2 fragmentation. Therefore, a formic acid digest leading to smaller peptide fragments was employed in the following experiments.

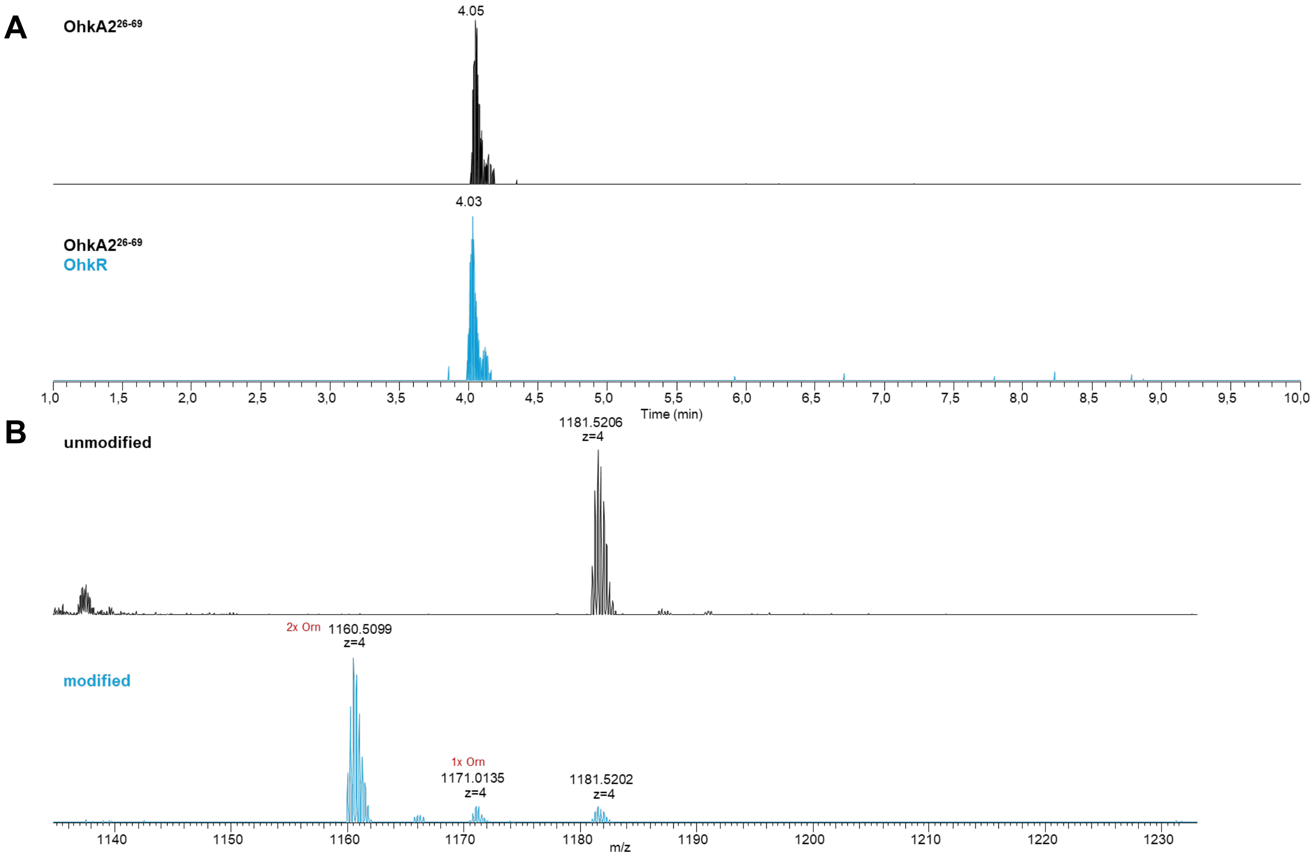

Figure S12. HPLC-MS analysis of OhkR arginase (blue) with native substrate OhkA2: LysC digested precursor of *ohkA2* and *ohkR* (co-)expression. (**A**) EICs for the $[M+4H]^{4+}$ *m/z* 1181.5525 for the unmodified and $[M+4H]^{4+}$ *m/z* 1160.5116 for the 2-Orn species, respectively. (**B**) MS^1 of the unmodified (substrate) and modified (product) peaks. Orn = ornithine. MS^2 analysis was not possible because the resulting fragment ($OhkA2^{26-69}$) was too large for MS^2 fragmentation. Therefore, a formic acid digest was employed in the following experiments.

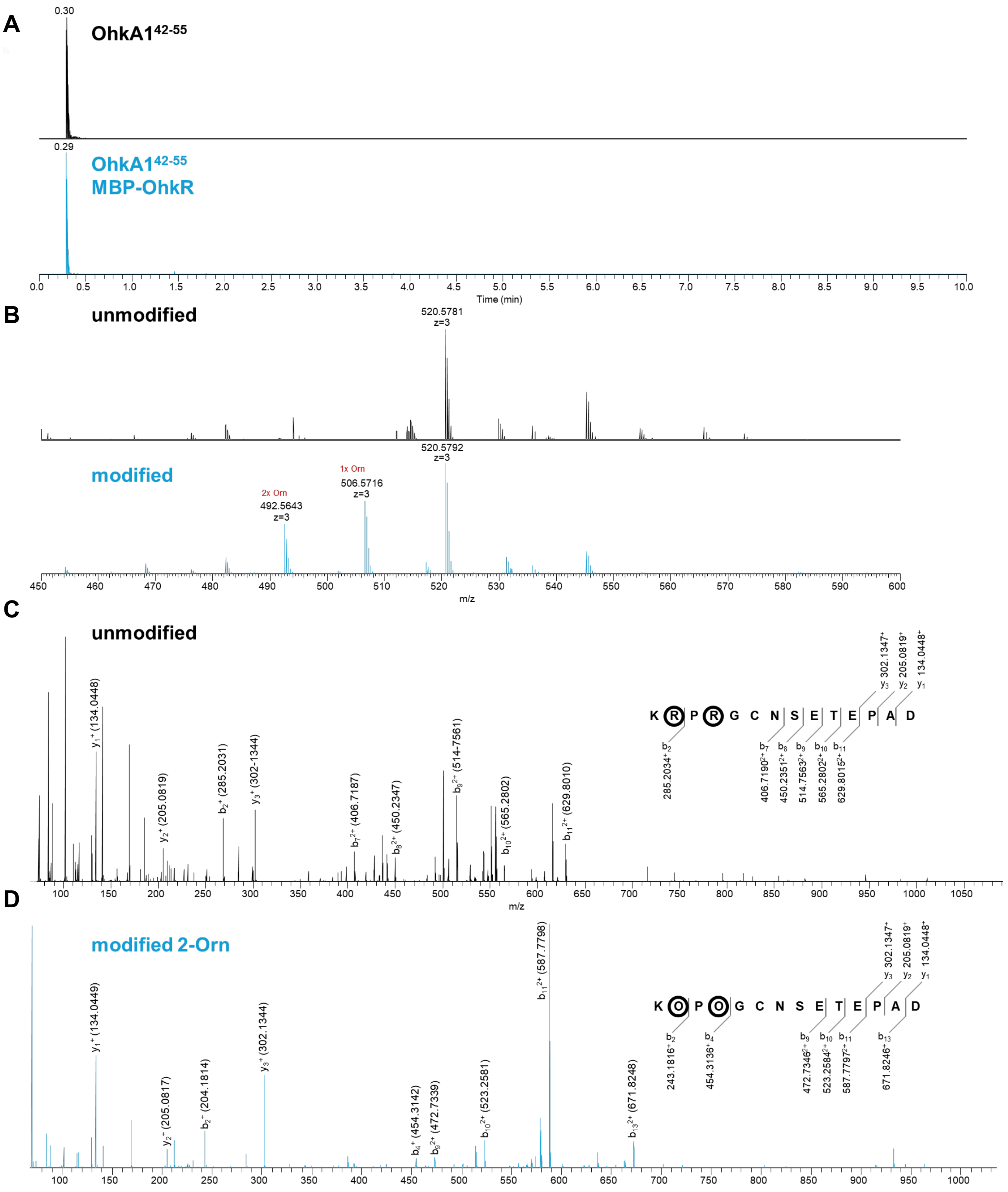

Figure S13. HPLC-MS/MS^2 analysis of MBP-tagged OhkR arginase with its native substrate OhkA1: Formic acid digested precursor of *ohkA1* and *MBP-ohkR* (co-)expression. (**A**) EICs for the $[M+3H]^{3+}$ *m/z* 520.5792 for the unmodified and 492.5647 for the 2-Orn species. (**B**) MS^1 of the unmodified (substrate) and modified (product) peaks. (**C**) MS^2 spectrum of the unmodified product (*m/z* 520.5792, $[M+3H]^{3+}$; peptide sequence and calculated monoisotopic mass of detected fragment ions are shown on the right). (**D**) MS^2 spectrum of the modified product (2-Orn, *m/z* 492.567), $[M+3H]^{3+}$; peptide sequence and calculated monoisotopic mass of detected fragment ions are shown on the right. O / Orn = ornithine.

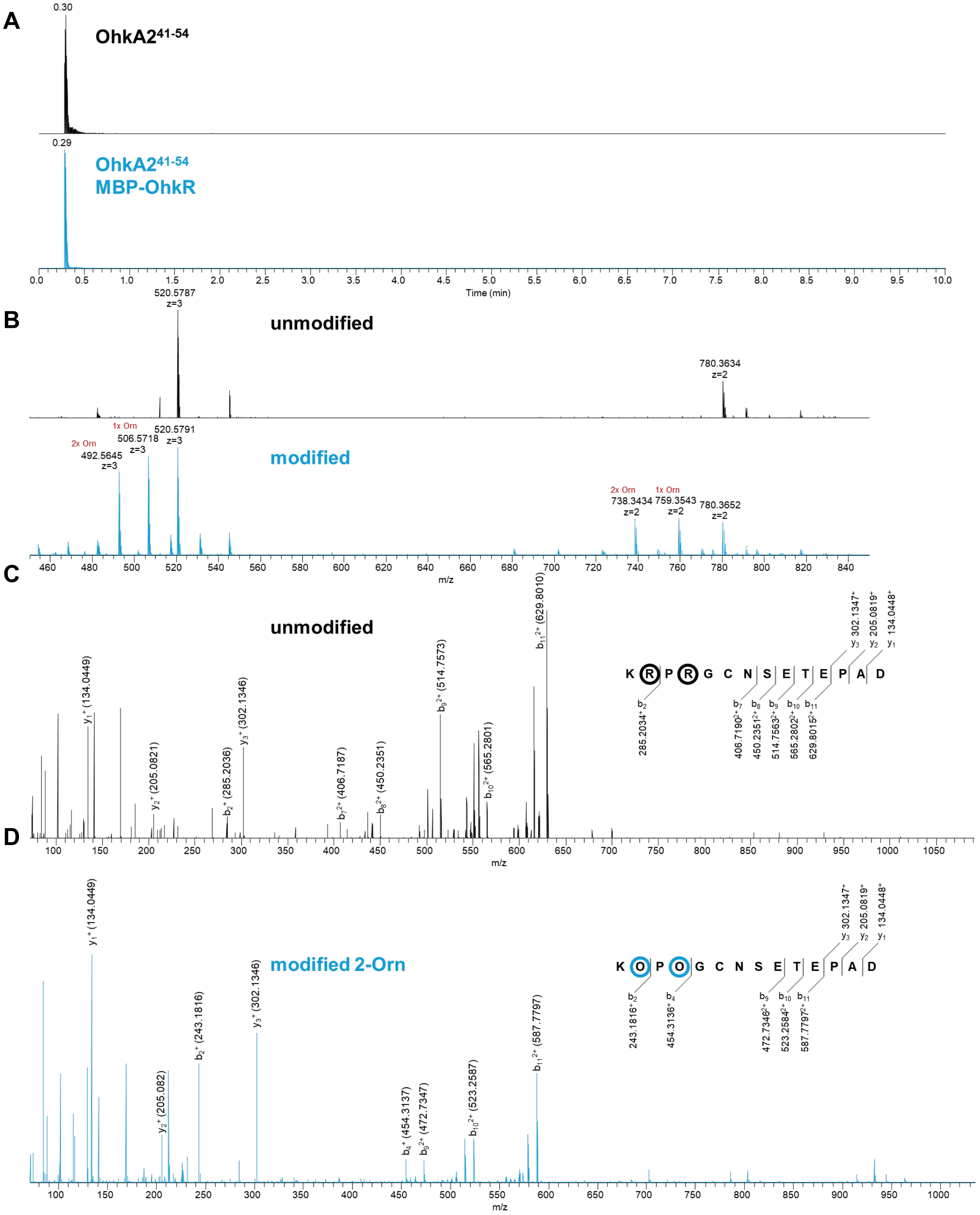

Figure S14. HPLC-MS/MS2 analysis of MBP-tagged OhkR arginase with its native substrate OhkA1: Formic acid digested precursor of *ohkA1* and *MBP-ohkR* (co-)expression **(A)** EICs for the $[M+3H]^{3+}$ m/z 520.5792 for the unmodified and 492.5647 for the 2-Orn species. **(B)** MS1 of the unmodified (substrate) and modified (product) peaks. **(C)** MS2 spectrum of the unmodified product (*m/z* 520.5792, $[M+3H]^{3+}$; peptide sequence and calculated monoisotopic mass of detected fragment ions are shown on the right. **(D)** MS2 spectrum of the modified product (2-Orn, *m/z* 492.567), $[M+3H]^{3+;}$ peptide sequence and calculated monoisotopic mass of detected fragment ions are shown on the right). O / Orn = ornithine.

Directionality of arginine-to-ornithine conversion for OhkR

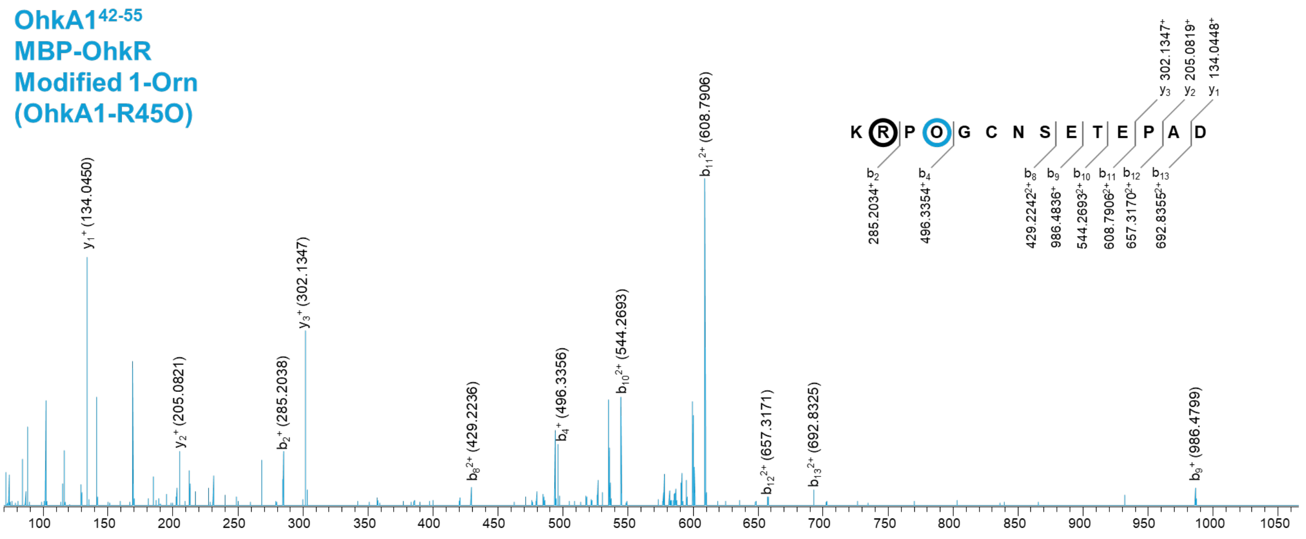

Figure S15. HPLC-MS^2 analysis of OhkR arginase with its native substrate OhkA1: Formic acid digested precursor of *ohkA1* and *MBP-OhkR* co-expression. MS^2 spectrum of the modified product (1-Orn, *m/z* 506.5719 $[M+3H]^{3+}$; peptide sequence and calculated monoisotopic mass of detected fragment ions are shown on the right. O / Orn = ornithine.

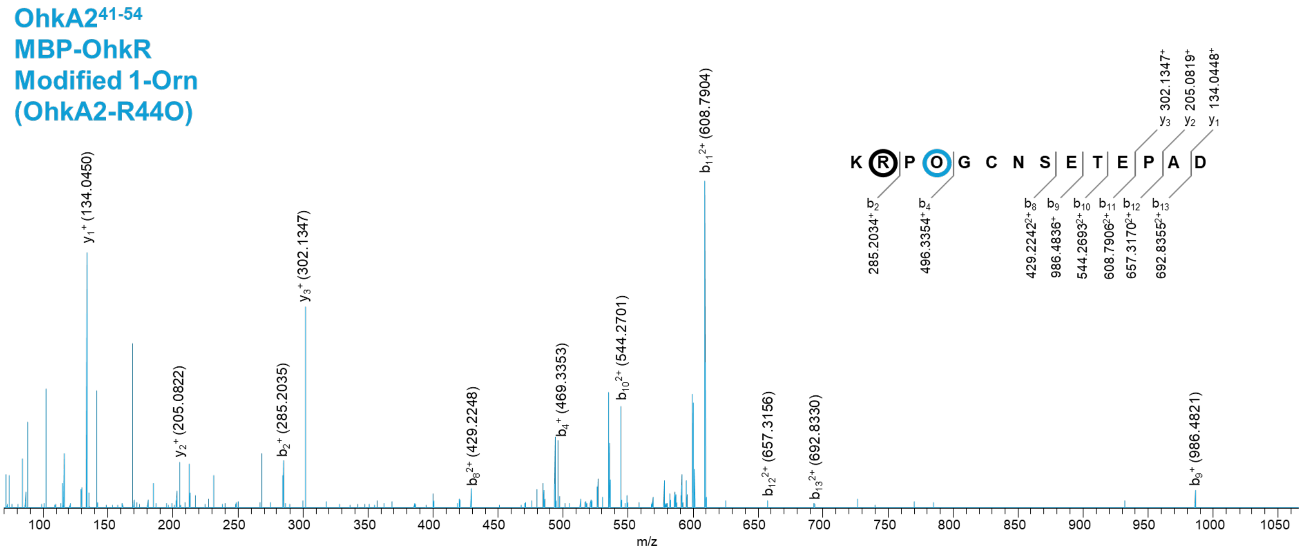

Figure S16. HPLC-MS^2 analysis of OhkR arginase with its native substrate OhkA2: Formic acid digested precursor of *ohkA2* and *MBP-ohkR* co-expression. MS^2 spectrum of the modified product (1-Orn, *m/z* 506.5719 $[M+3H]^{3+}$; peptide sequence and calculated monoisotopic mass of detected fragment ions are shown on the right. O / Orn = ornithine.

Precursor Swapping: Activity of OhkR on the FlmR Substrates FlmA1, FlmA2, and FlmA3 (*in vivo*)

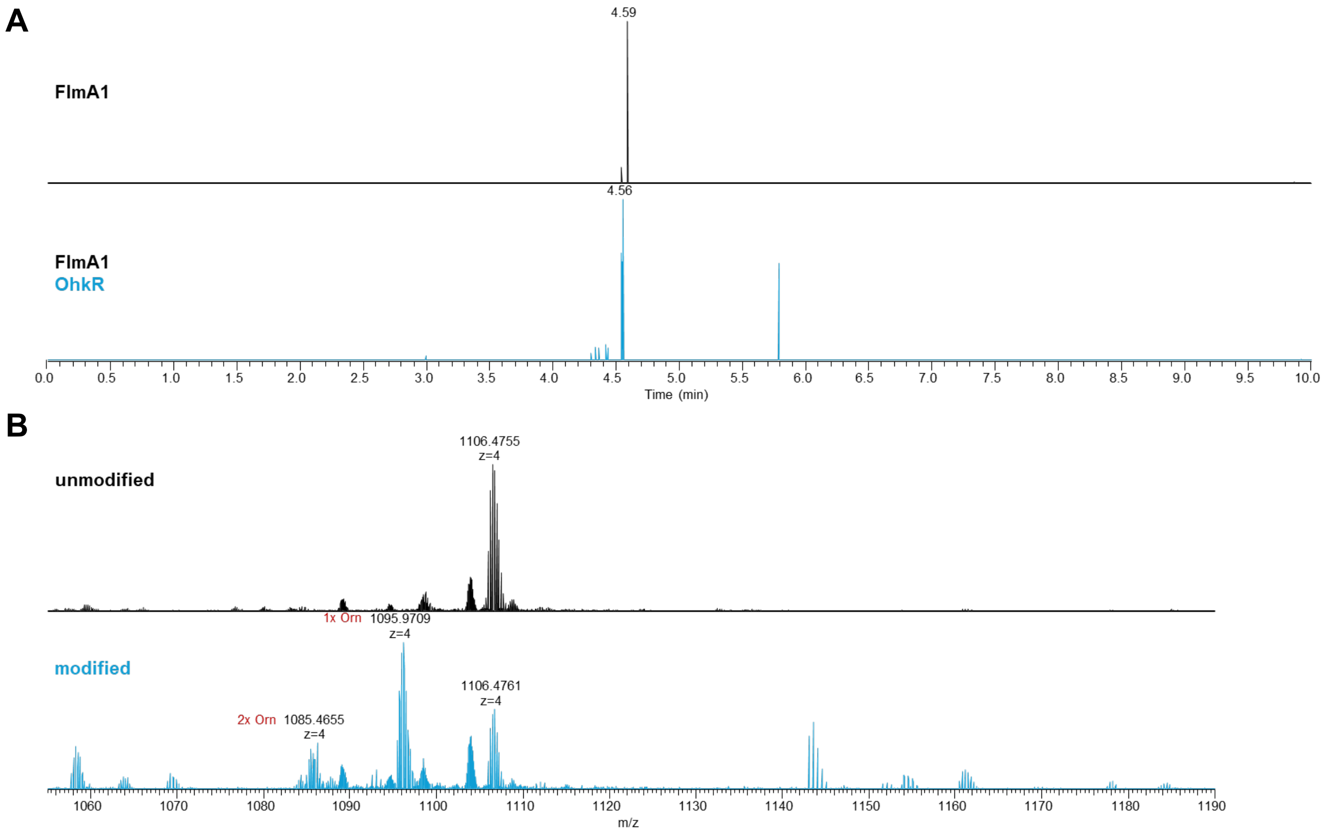

Figure S17. HPLC-MS analysis of OhkR arginase (blue) with non-native substrate FlmA1: LysC digested precursor of *flmA1* and *ohkR* (co-)expression. (**A**) EICs for the $[M+4H]^{4+}$ *m/z* 1106.4755, 1106.4761 for the unmodified and $[M+4H]^{4+}$ *m/z* 1095.9709, 1085.4655 for the 1-Orn, 2-Orn species, respectively. (**B**) MS^1 of the unmodified (substrate) and modified (product) peaks. Orn = ornithine.

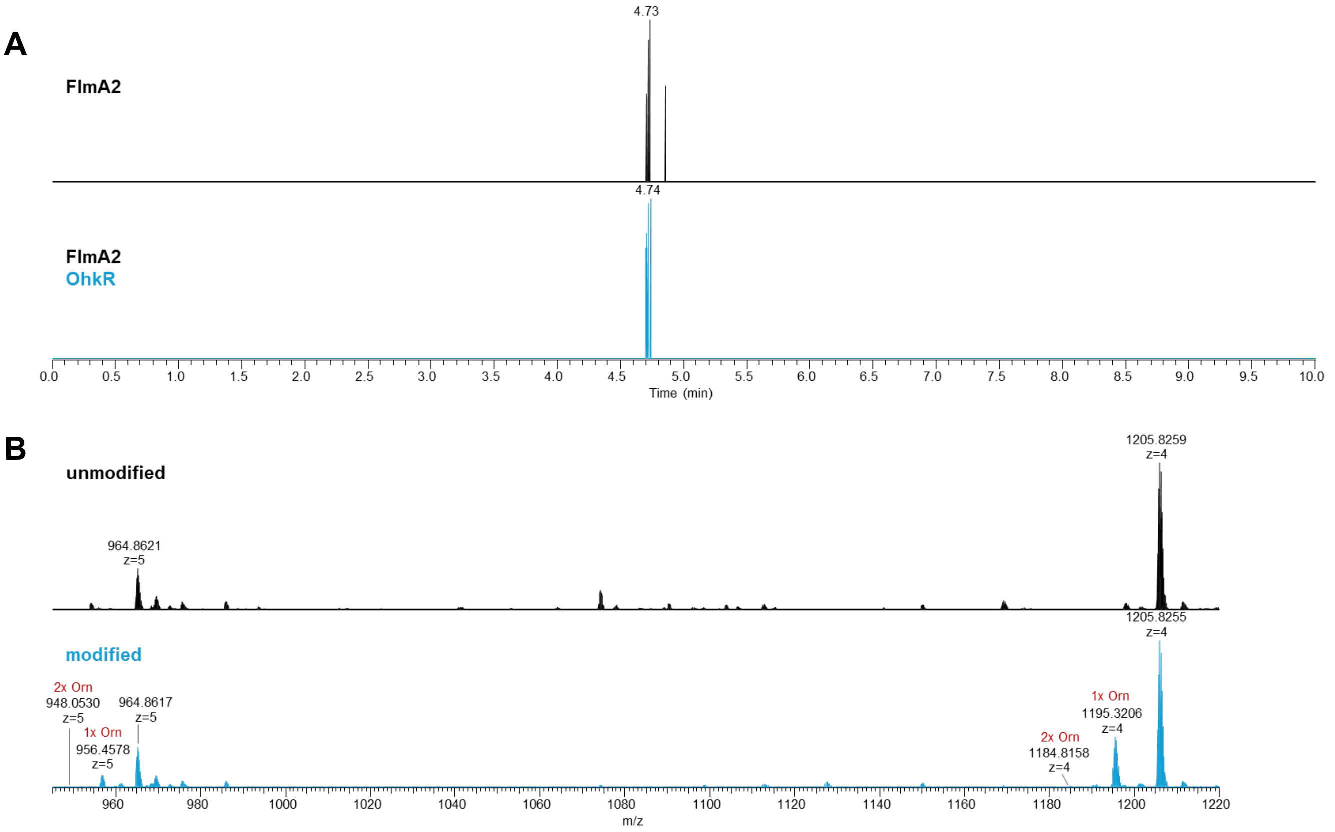

Figure S18. HPLC-MS analysis of OhkR arginase (blue) with non-native substrate FlmA2: LysC digested precursor of *flmA2* and *ohkR* (co-)expression. (**A**) EICs for the $[M+4H]^{4+}$ *m/z* 1205.8259, 1205.8255; $[M+5H]^{5+}$ *m/z* 964.8621, 964.8617 for the unmodified; $[M+4H]^{4+}$ *m/z* 1195.3206; $[M+5H]^{5+}$ *m/z* 956.4578 for the 1-Orn species and $[M+4H]^{4+}$ *m/z* 1184.8158; $[M+5H]^{5+}$ *m/z* 948.0530 for the 2-Orn species. (**B**) MS^1 of the unmodified (substrate) and modified (product) peaks. Orn = ornithine.

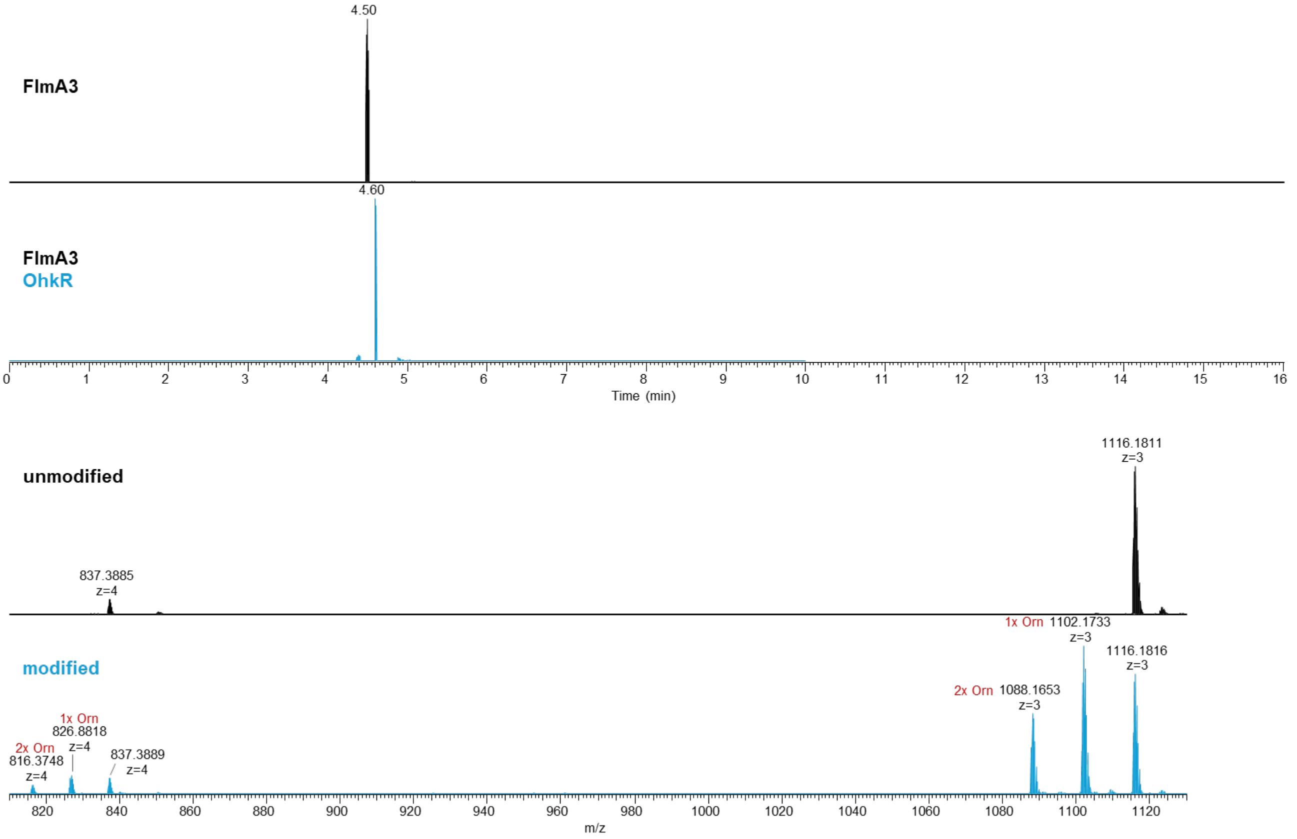

Figure S19. HPLC-MS analysis of OhkR arginase (blue) with non-native substrate FlmA3: LysC digested precursor of *flmA3* and *ohkR* (co-)expression. (**A**) EICs for the $[M+3H]^{3+}$ *m/z* 1116.1811, 1116.1816; $[M+4H]^{4+}$ *m/z* 837.3885, 837.3889 for the unmodified; $[M+3H]^{3+}$ *m/z* 1102.1733; $[M+4H]^{4+}$ *m/z* 826.8818 for the 1-Orn species and $[M+3H]^{3+}$ *m/z* 1088.1653; $[M+4H]^{4+}$ *m/z* 816.3748 for the 2-Orn species. (**B**) MS^1 of the unmodified (substrate) and modified (product) peaks. Orn = ornithine.

Precursor Swapping: Activity of FlmR on the OhkR Substrates OhkA1 and OhkA2 (*in vivo*)

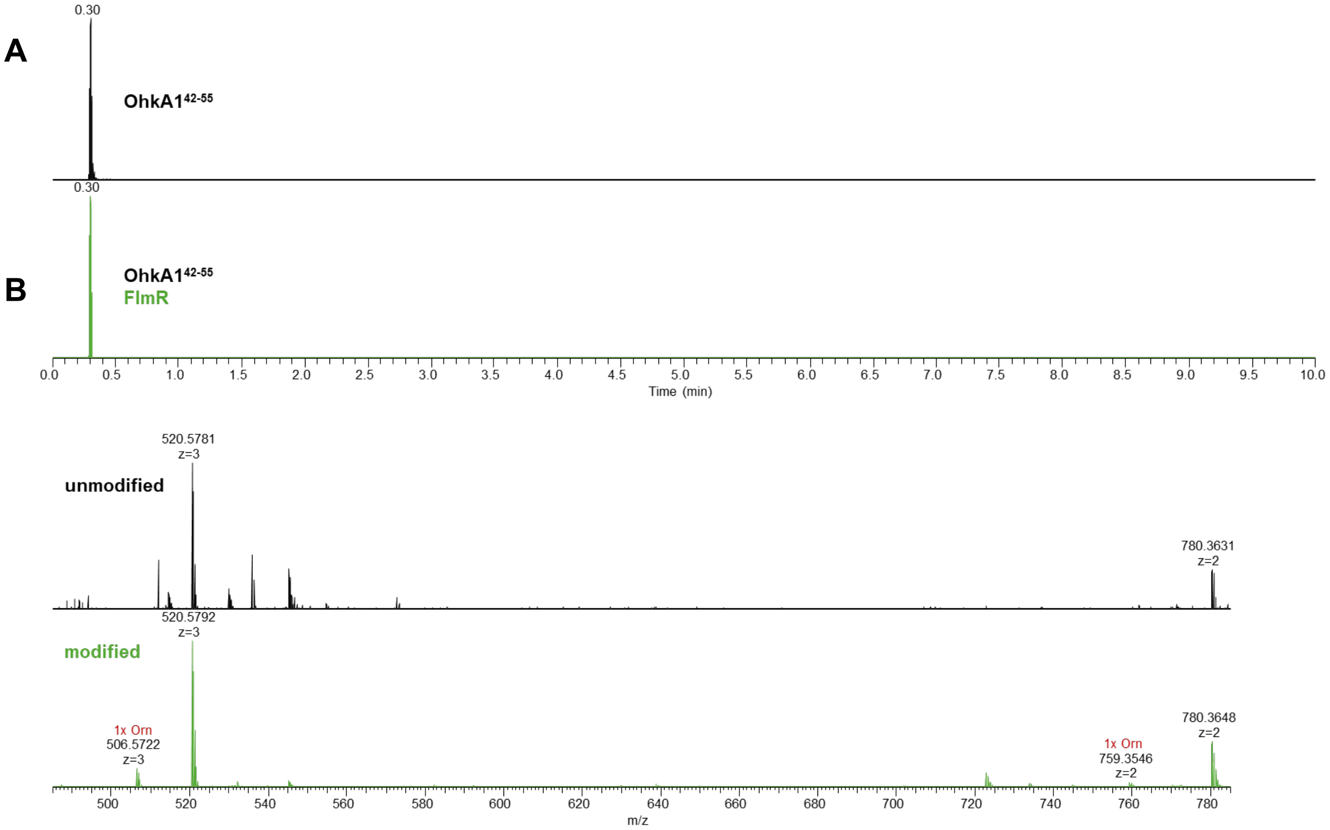

Figure S20. HPLC-MS analysis of FlmR arginase (green) with non-native substrate OhkA1: Formic acid digested precursor of *ohkA1* and His-*flmR* (co-)expression. (**A**) EICs for the $[M+2H]^{2+}$ *m/z* 780.3631; $[M+3H]^{3+}$ *m/z* 520.5781 for the unmodified; $[M+2H]^{2+}$ *m/z* 759.3546; $[M+3H]^{3+}$ *m/z* 506.5722 for the 1-Orn species. (**B**) MS^1 of the unmodified (substrate) and modified (product) peaks. Orn = ornithine.

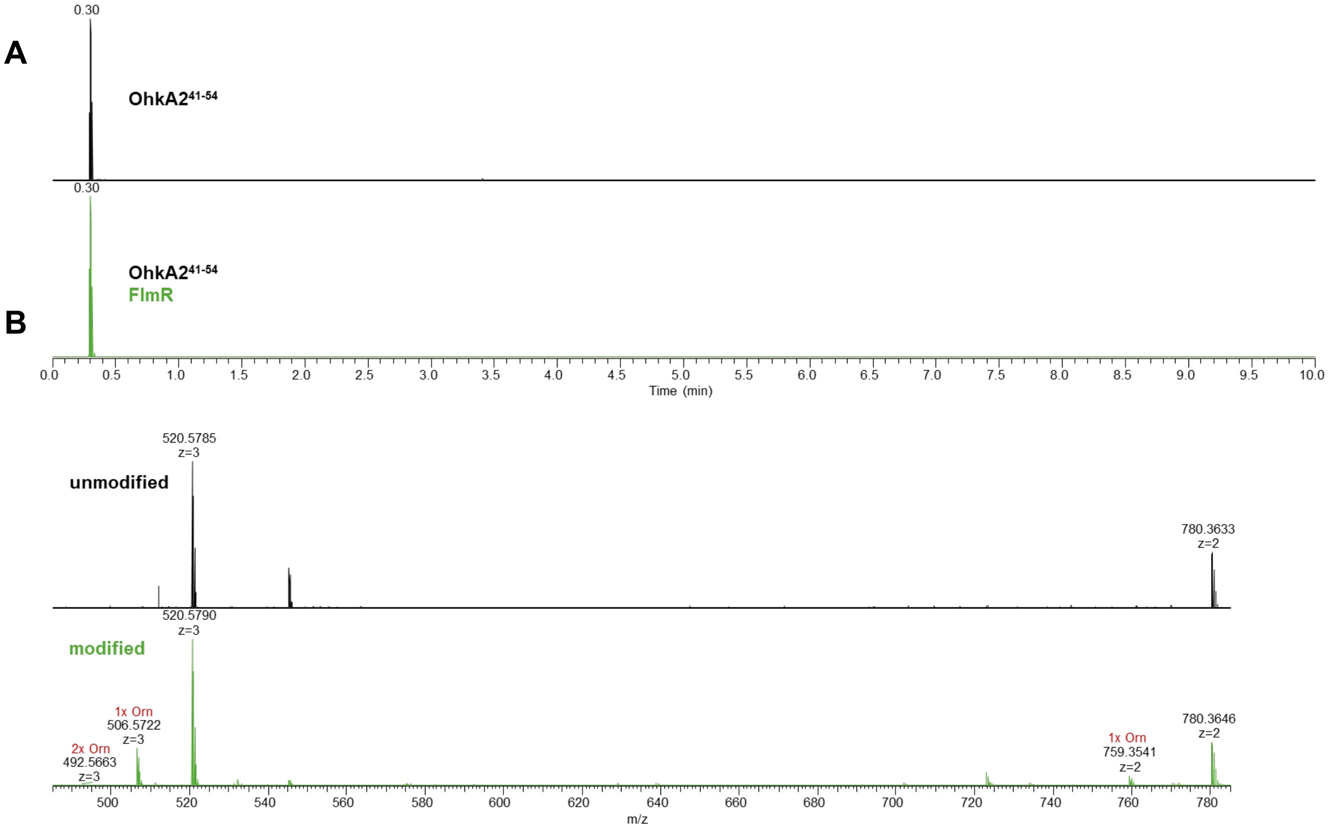

Figure S21. HPLC-MS analysis of FlmR arginase (green) with non-native substrate OhkA2: Formic acid digested precursor of *ohkA1* and His-*flmR* (co-)expression. (**A**) EICs for the $[M+2H]^{2+}$ *m/z* 780.3633; $[M+3H]^{3+}$ *m/z* 520.5785 for the unmodified; $[M+2H]^{2+}$ *m/z* 759.3541; $[M+3H]^{3+}$ *m/z* 506.5722 for the 1-Orn species and $[M+3H]^{3+}$ *m/z* 492.5663 for the 2-Orn species (**B**) MS^1 of the unmodified (substrate) and modified (product) peaks. Orn = ornithine.

Alanine replacement of the conserved 'DD(I/V)LF' motif of FlmA1 and OhkA1 precursors

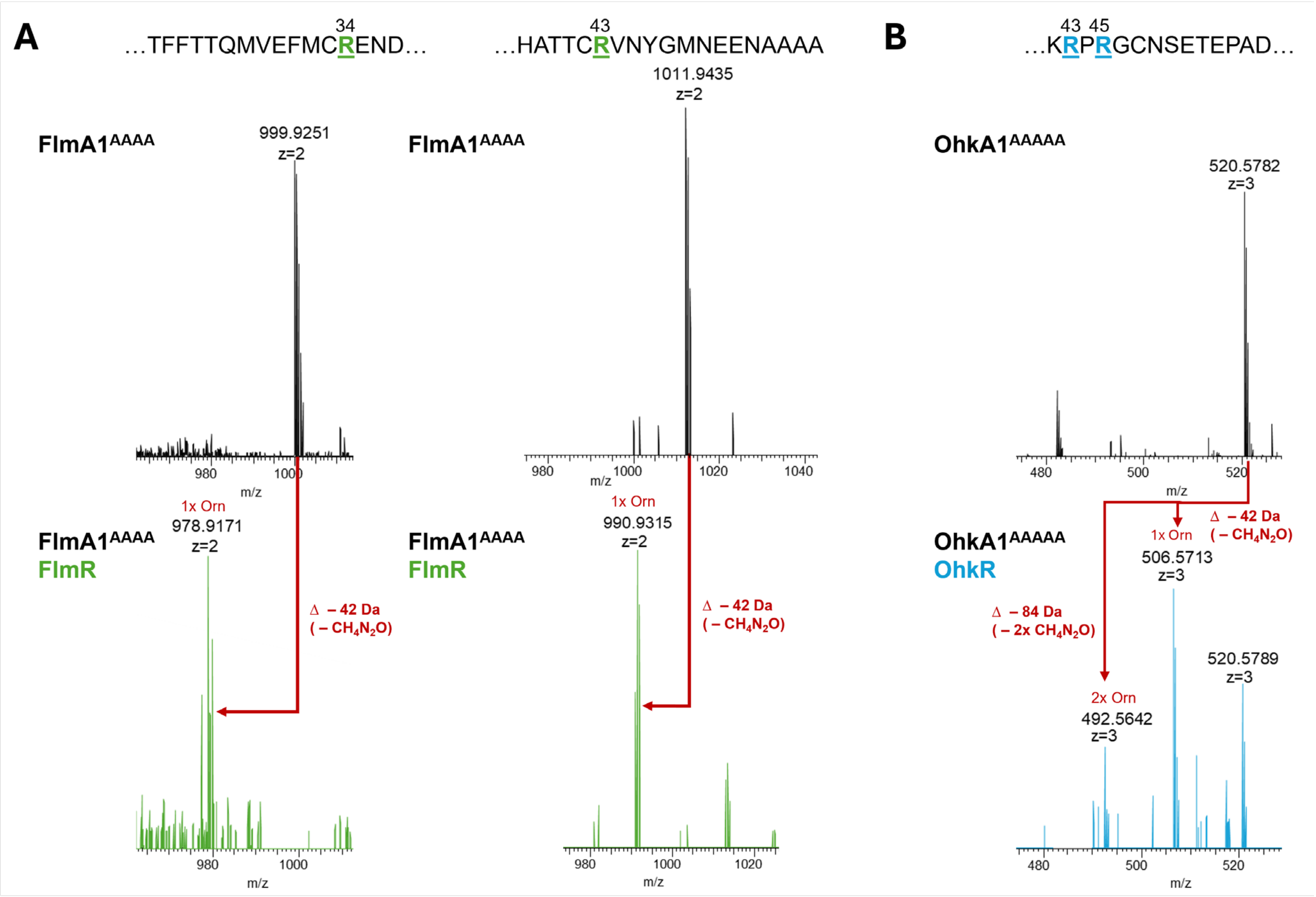

Figure S22. *In vivo* co-expression experiments of precursor variants with alanine residues replacing the 'DD(I/V)LF' motif with FlmR or OhkR. HPLC-MS analysis of modified precursor peptides compared to the control (black traces) of (**A**) FlmA1-D53A-I54A-L55A-F56A (FlmA1^{AAAA}) co-expressed with FlmR (green traces) and (**B**) OhkA1-D66A-D67A-V68A-L69A-F70A (OhkA1^{AAAAA}) co-expressed with OhkR (blue traces). Full scan spectra of MS^1 data are depicted. A mass loss of 42.02 Da per arginine hydrolysed to ornithine and urea is observed.

Michaelis-Menten kinetics for FlmR and OhkR

Table S12. Kinetic data of FlmR and OhkR with their native substrates FlmA1 and OhkA1, respectively, in comparison with data of OspR (grey). The maximum velocity, the Michaelis-Menten constant (K_M), the catalytic constant/turnover number (k_{cat}), and the catalytic efficiency (k_{cat}/K_M) are listed. SD = standard deviation.

	FlmR (for FlmA1)	OhkR (for OhkA1)	OspR (for OspA)[1]
V_{max} (mean +/- SD)	$9.13 \times 10^{-4} \pm 1.20 \times 10^{-4}$ s^{-1}	$10.3 \times 10^{-4} \pm 4.08 \times 10^{-4}$ s^{-1}	25.39×10^{-3} s^{-1}
K_M (mean +/- SD)	8.42 ± 2.08 µM	2.34 ± 1.01 µM	9.01 ± 1.38 µM
k_{cat}	9.13×10^{-5} s^{-1}	10.3×10^{-5} s^{-1}	5.078×10^{-3} s^{-1}
k_{cat}/K_M	10.84 $M^{-1}s^{-1}$	43.98 $M^{-1}s^{-1}$	564 M^{-1} s^{-1}

Relative Rates for FlmA1, FlmA2, FlmA3, OhkA1, and OhkA2 and Testing of L-Arginine as a Substrate

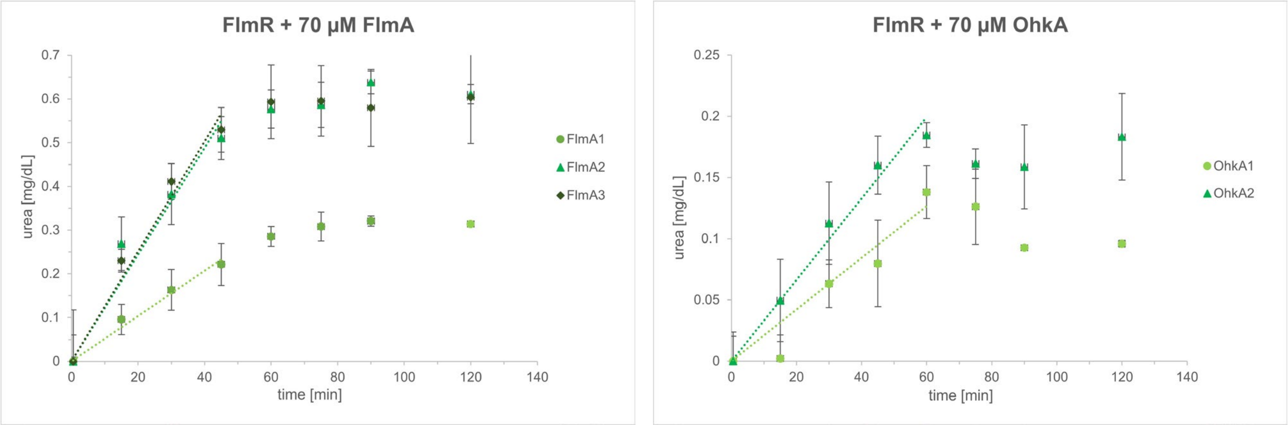

Figure S24. Relative rates of FlmR with FlmA1-A3 (left) and OhkA1-A2 (right) substrates.

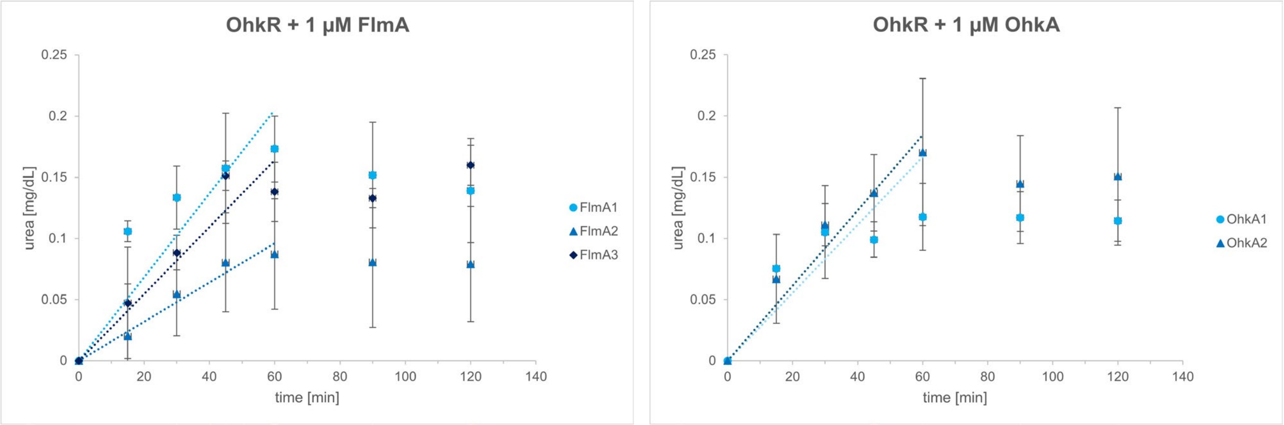

Figure S23. Relative rates of OhkR with FlmA1-A3 (left) and OhkA1-A2 (right) substrates.

Table S13. Relative rates of arginine-to-ornithine conversion in FlmA and OhkA precursors catalysed by FlmR or MBP-OhkR.

	FlmR	OhkR
FlmA1	41.3 %	100 %
FlmA2	96.8 %	47.1 %
FlmA3	100 %	79.4 %
OhkA1	16.7 %	82.4 %
OhkA2	26.2 %	91.2 %

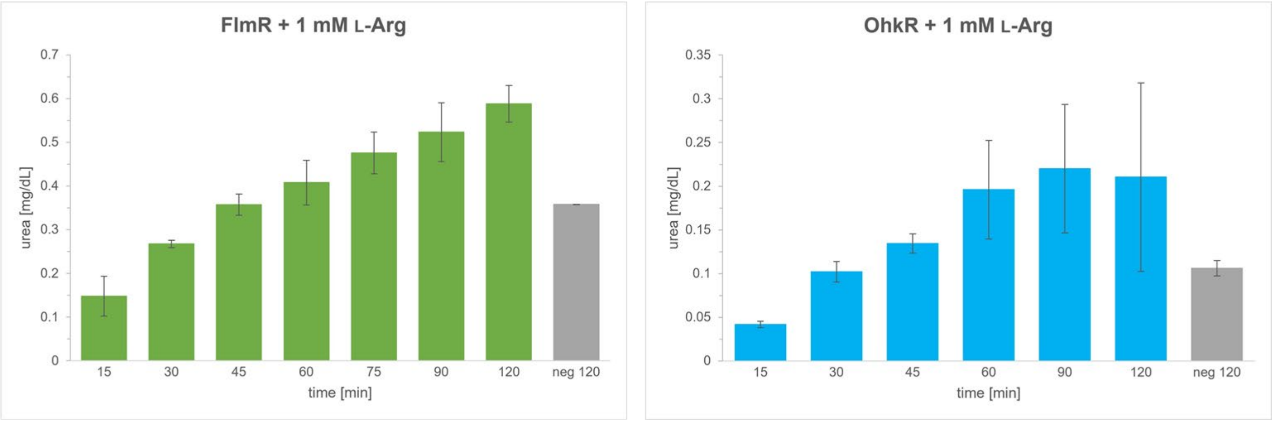

Figure S25. Urea concentration of arginine-to-ornithine conversion of L-Arg by FlmR (left) and by MBP-OhkR (right). Urea concentration of negative control (incubation without FlmR or OhkR) after 120 min is depicted in grey.

In silico Structural Analysis of Precursors, Arginases, and Precursor-Arginase Binding

Table S14. pTM, ipTM and calculated pLDDT values of AF3 models of Flm (**A**) and Ohk (**B**). The predicted template modelling (pTM) score measures the accuracy of the entire predicted structure, the interface predicted template modelling (ipTM) score the accuracy of the predicted subunit positions within the complex. Models with pTM and ipTM scores above 0.8 are predictions with high confidence.[8] The predicted local distance difference test (pLDDT) score gives a per-atom confidence. The calculated pLDDT represents the average of the overall predicted structure.

A

Entities	pTM	ipTM	pLDDT
2x FlmR	0.89	0.9	90.0899
2x FlmA1	0.32	0.23	47.2467
2x FlmA2	0.22	0.1	39.8745
2x FlmA3	0.39	0.33	57.7669
2x FlmA1 (2x FlmR)	-	-	38.2814
2x FlmA2 (2x FlmR)	-	-	39.4088
2x FlmA3 (2x FlmR)	-	-	53.2745
1x FlmA1 2x FlmR	0.85	0.82	83.7610
1x FlmA2 2x FlmR	0.85	0.82	84.5171
1x FlmA3 2x FlmR	0.86	0.85	86.6099
2x FlmA1 2x FlmR	0.84	0.80	79.5402
2x FlmA2 2x FlmR	0.84	0.80	79.8548
2x FlmA3 2x FlmR	0.85	0.83	83.4364
2x FlmA1 (R34K) 2x FlmR	0.84	0.80	83.7560
2x FlmA1 (R43K) 2x FlmR	0.88	0.85	83.7487
2x FlmA2 (R33K) 2x FlmR	0.85	0.83	82.3944
2x FlmA2 (R42K) 2x FlmR	0.83	0.79	79.7213
2x FlmA3 (R43K) 2x FlmR	0.84	0.80	81.3788
2x FlmA3 (R46K) 2x FlmR	0.85	0.81	81.4901

B

Entities	pTM	ipTM	pLDDT
2x OhkR	0.89	0.89	90.0234
2x OhkA1	0.29	0.26	51.0108
2x OhkA2	0.40	0.38	52.2698
2x OhkA1 (2x OhkR)	-	-	38.5926
2x OhkA2 (2x OhkR)	-	-	34.5087
1x OhkA1 2x OhkR	0.83	0.77	83.7635
1x OhkA2 2x OhkR	0.84	0.78	84.7605
2x OhkA1 2x OhkR	0.82	0.74	81.1139
2x OhkA2 2x OhkR	0.81	0.73	80.6402
2x OhkA1 (R43K) 2x OhkR	0.81	0.73	81.1575
2x OhkA1 (R45K) 2x OhkR	0.83	0.76	81.7534
2x OhkA2 (R42K) 2x OhkR	0.82	0.74	81.2709
2x OhkA2 (R44K) 2x OhkR	0.81	0.73	80.7229

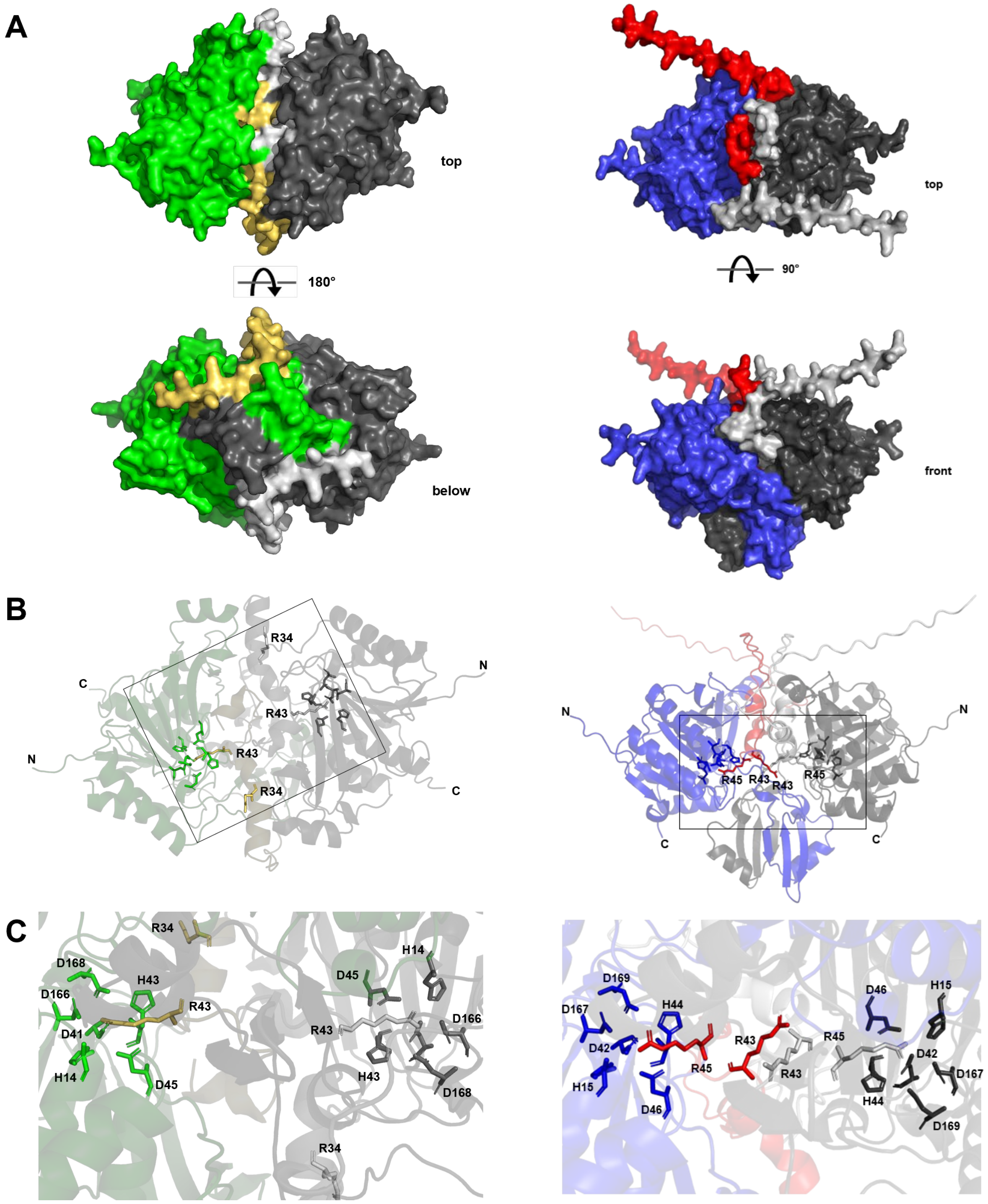

Figure S26. AlphaFold3-modelled complexes of FlmR with FlmA1 (left) and OhkR with OhkA1 (right). One arginase monomer is depicted in green (FlmR) or blue (OhkR), and one precursors monomer in yellow/red. The second monomer is coloured in grey. (**A**) Surface-representation of the FlmR/OhkR homodimer from top and below. (**B**) Cartoon-representation of FlmR homodimer from top and OhkR homodimer from front. The arginase active side residues and precursor arginine residues are shown as sticks. (**C**) Zoomed-in section of substrate binding with active site residues (green/blue) and arginine substrate (yellow/red) shown as sticks and labelled by the one-letter-code (180° turned compared to **B**).

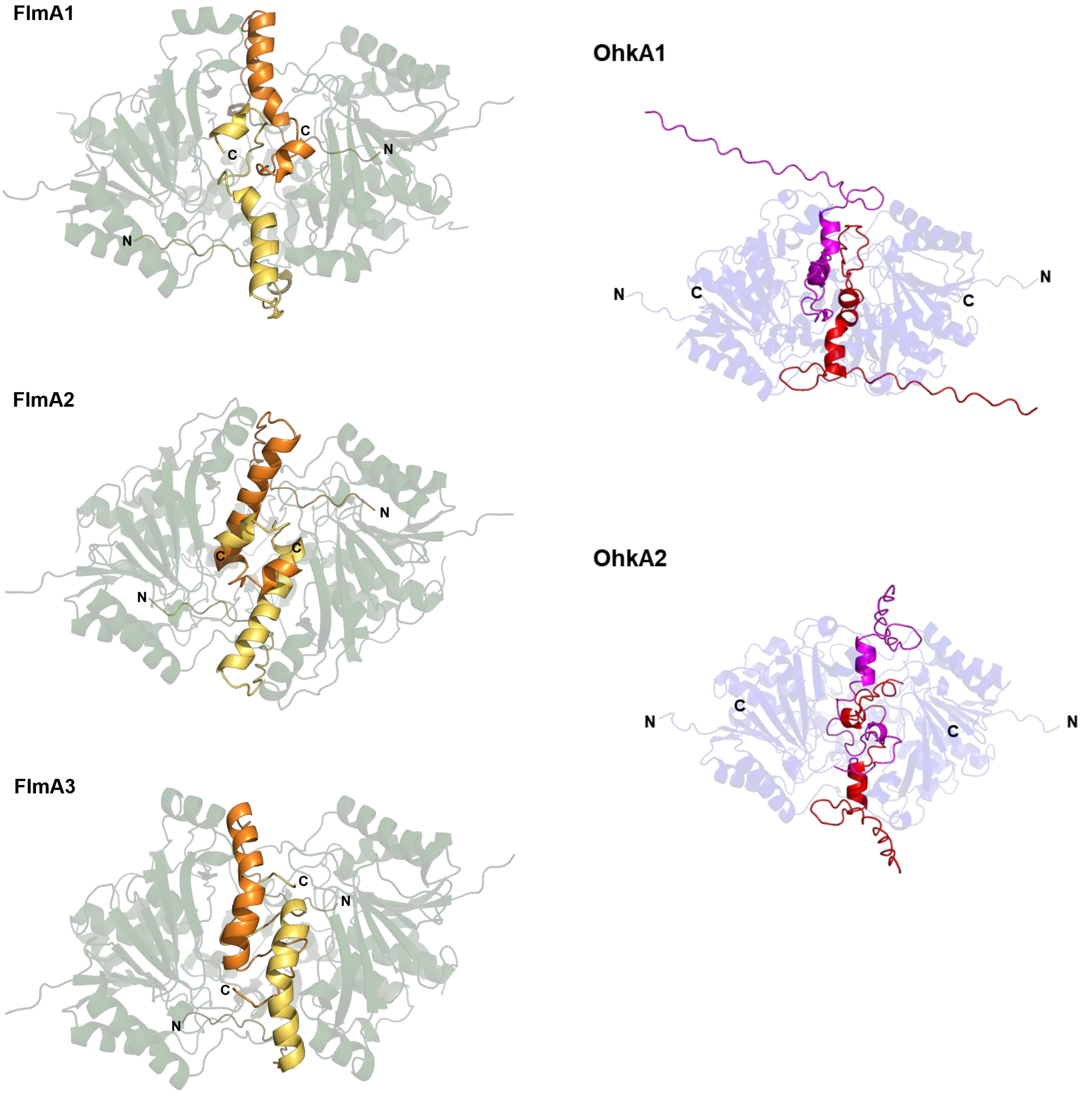

Figure S27. Left: AlphaFold3-modelled homodimer complexes of FlmR with precursors FlmA1, FlmA2, and FlmA3. Position of the precursor monomers (depicted in yellow and orange) in complex with FlmR arginase (transparent green) is shown. Right: AlphaFold-modelled homodimer complexes of OhkR with precursors OhkA1 and OhkA2. Position of the precursor monomers (depicted in red and magenta) in complex with Ohk arginase (transparent blue) is shown from the top.

PISA (Proteins, Interfaces, Structures and Assemblies) analysis

Table S15. PISA (Proteins, Interfaces, Structures and Assemblies) analysis[9] of Flm and Ohk homodimer arginase-precursor AlphaFold3 models. Each homodimer consists of the monomers A and B. The interface area Å^2, areas of highly abundant interfacing residues in the homodimer, the percentage of interface area in the entire chain and the ΔiG P-value are shown. The Δ^{i}G P-value is a measurement for the interface specificity and implies at Δ^{i}G P<0.5 interaction specificity.

	Monomers	Interface area [Å^2]	Dimer interfacing residues	Percentage of interface area in the entire chain		Δ^{i}G P-value
				Chain 1	Chain 2	
**FlmR**	R^A–R^B	2515.0	Pro^{176} – Ser^{232}	14.7	14.7	0.565
**FlmR FlmA1**	R^A–R^B	2428.2	Pro^{176} – Tyr^{229}	14.1	14.0	0.621
	A1^A–A1^B	642.5	Arg^{34} – Phe^{56}	9.3	9.2	0.838
	R^A–A1^A	1836.7		9.9	28.4	0.230
	R^A–A1^B	996.7		5.7	14.6	0.459
**FlmR FlmA2**	R^A–R^B	2380.9	Pro^{176} – Tyr^{229}	14.2	14.2	0.595
	A2^A–A2^B	813.3	Cys^{32} – Phe^{55}	12.4	12.6	0.745
	R^A–A2^A	1624.7		9.4	25.6	0.328
	R^A–A2^B	1364.8		7.4	23.1	0.489
**FlmR FlmA3**	R^A–R^B	2440.7	Pro^{176} – Tyr^{229}	14.4	14.4	0.724
	A3^A–A3^B	1108.7	Lys^{25} – Phe^{56}	17.0	17.0	0.463
	R^A–A3^A	1472.2		7.9	24.6	0.166
	R^A–A3^B	1125.1		6.1	18.6	0.502
**OhkR**	R^A–R^B	2310.5	Pro^{177} – Pro^{231}	13.4	13.5	0.112
**OhkR OhkA1**	R^A–R^B	2379.0	Pro^{177} – Pro^{233}	13.8	13.8	0.199
	A1^A–A1^B	1053.2	Ser^{35} – Phe^{70}	13.1	12.8	0.879
	R^A–A1^A	1593.6		9.0	21.6	0.542
	R^A–A1^B	581.5		3.6	6.8	0.690
**OhkR OhkA2**	R^A–R^B	2367.4	Pro^{177} – Pro^{233}	13.8	13.8	0.164
	A2^A–A2^B	1198.4	Leu^{32} – Phe^{69}	14.4	14.4	0.679
	R^A–A2^A	1427.7		7.4	19.0	0.532
	R^A–A2^B	912.9		5.5	10.6	0.560

Analysis of precursor variants: Deletion of OhkA1 and OhkA2 N-termini

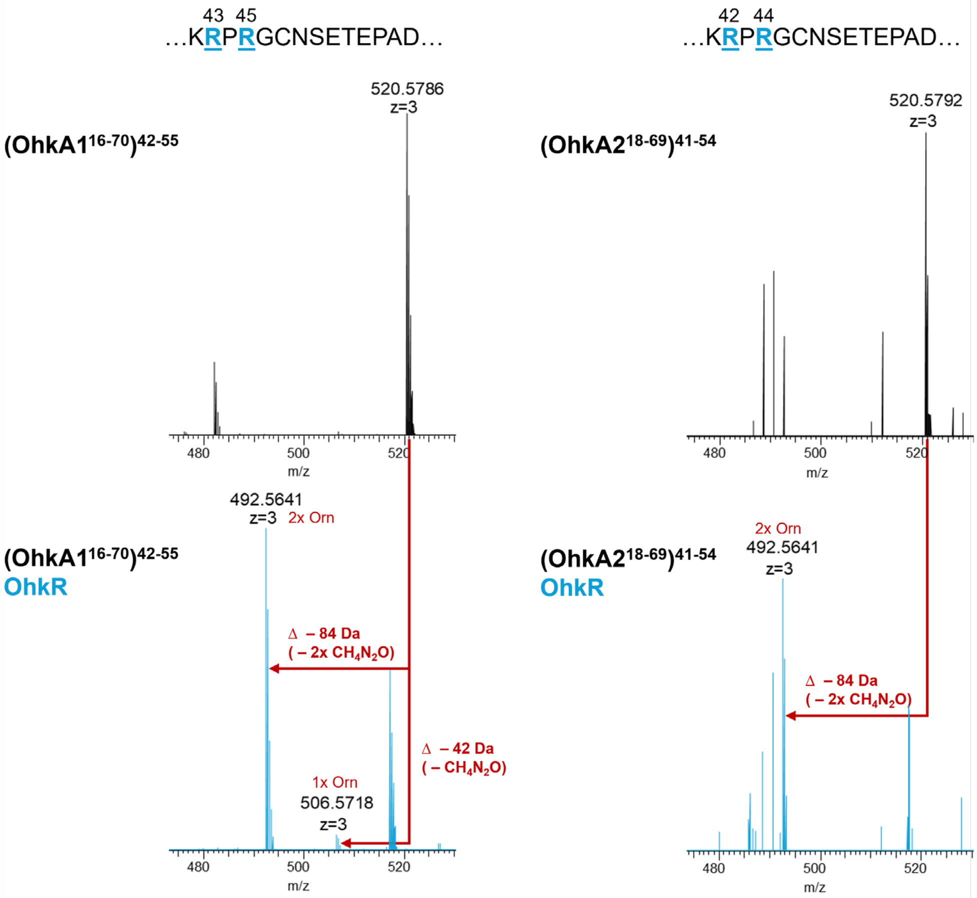

Figure S28. *In vivo* co-expression experiments of Ohk precursor variants with OhkR. HPLC-MS analysis of modified precursor peptides compared to the control (black traces) of OhkA1$^{16\text{-}70}$ and OhkA2$^{18\text{-}69}$ co-expressed with OhkR. Full scan spectra of MS^1 data are depicted. A mass loss of 42.02 Da per arginine hydrolysed to ornithine and urea is observed.

References

[1] S. Mordhorst, T. Badmann, N. M. Bösch, B. I. Morinaka, H. Rauch, J. Piel, M. Groll, A. L. Vagstad, "Structural and Biochemical Insights into Post-Translational Arginine-to-Ornithine Peptide Modifications by an Atypical Arginase" *ACS Chem. Biol.* **2023**, *18*, 528–536.

[2] R. B. Kapust, J. Tözsér, T. D. Copeland, D. S. Waugh, "The P1′ specificity of tobacco etch virus protease" *Biochem. Biophys. Res. Commun.* **2002**, *294*, 949–955.

[3] Origin(Pro), Version 2025b. OriginLab Corporation, Northampton, MA, USA.

[4] J. Abramson, J. Adler, J. Dunger, R. Evans, T. Green, A. Pritzel, O. Ronneberger, L. Willmore, A. J. Ballard, J. Bambrick, S. W. Bodenstein, D. A. Evans, C.-C. Hung, M. O'Neill, D. Reiman, K. Tunyasuvunakool, Z. Wu, A. Žemgulytė, E. Arvaniti, C. Beattie, O. Bertolli, A. Bridgland, A. Cherepanov, M. Congreve, A. I. Cowen-Rivers, A. Cowie, M. Figurnov, F. B. Fuchs, H. Gladman, R. Jain, Y. A. Khan, C. M. R. Low, K. Perlin, A. Potapenko, P. Savy, S. Singh, A. Stecula, A. Thillaisundaram, C. Tong, S. Yakneen, E. D. Zhong, M. Zielinski, A. Žídek, V. Bapst, P. Kohli, M. Jaderberg, D. Hassabis, J. M. Jumper, "Accurate structure prediction of biomolecular interactions with AlphaFold 3" *Nature* **2024**, *630*, 493–500.

[5] F. Madeira, N. Madhusoodanan, J. Lee, A. Eusebi, A. Niewielska, A. R. N. Tivey, R. Lopez, S. Butcher, "The EMBL-EBI Job Dispatcher sequence analysis tools framework in 2024" *Nucleic Acids Res.* **2024**, *52*, W521–W525.

[6] X. Robert, P. Gouet, "Deciphering key features in protein structures with the new ENDscript server" *Nucleic Acids Res.* **2014**, *42*, W320–W324.

[7] E. Gasteiger, C. Hoogland, A. Gattiker, S. Duvaud, M. R. Wilkins, R. D. Appel, A. Bairoch in *Protein Identification and Analysis Tools on the Expasy Server*, Vol. 1 (Ed.: John M. Walker: The Proteomics Protocols Handbook), Humana Press, Totowa, NJ, **2005**, pp. 571–607.

[8] Y. Zhang, J. Skolnick, "Scoring function for automated assessment of protein structure template quality" *Proteins* **2004**, *57*, 702–710.

[9] E. Krissinel, K. Henrick, "Inference of Macromolecular Assemblies from Crystalline State" *J. Mol. Biol.* **2007**, *372*, 774–797.
